# β-sitosterol inhibits trimethylamine production by regulating the gut microbiota and attenuates atherosclerosis in ApoE^–/–^ mice

**DOI:** 10.3389/fcvm.2022.986905

**Published:** 2022-11-01

**Authors:** Weiping Wu, Wugao Liu, Huafu Wang, Wei Wang, Weihua Chu, Jing Jin

**Affiliations:** ^1^Department of Clinical Laboratory, People’s Hospital of Lishui, The Sixth Affiliated Hospital of Wenzhou Medical University, Lishui, Zhejiang, China; ^2^Department of Clinical Pharmacy, People’s Hospital of Lishui, The Sixth Affiliated Hospital of Wenzhou Medical University, Lishui, Zhejiang, China; ^3^Department of Microbiology, School of Life Sciences and Technology, China Pharmaceutical University, Nanjing, China

**Keywords:** β-sitosterol, gut microbiota, TMA, TMAO, atherosclerosis

## Abstract

The intestinal microbial metabolite trimethylamine (TMA), which is activated by flavin monooxygenase (FMO) to produce trimethylamine-*N*-oxide (TMAO), has been implicated in the pathogenesis of atherosclerosis (AS), leading to the development of therapeutic strategies for AS. This study aimed to investigate whether β-sitosterol can inhibit TMA production in ApoE^–/–^ mice by reshaping the gut microbial structure. 16S rRNA sequencing of the gut microbiota showed that β-sitosterol has beneficial effects on intestinal flora function, especially the inhibition of bacteria genera that contain the gene cholintrimethylamine lyase, which is responsible for the major pathway for TMA production. In parallel, β-sitosterol effectively reduced the TMA, FMO3, and TMAO levels while ameliorating the atherosclerotic plaques of AS mice. Moreover, β-sitosterol could alleviate cholesterol metabolism and the inflammatory response, and improve the antioxidant defense capacity. These studies offer new insights into the mechanisms responsible for the antiatherosclerotic effects of β-sitosterol, which targets the microbiota-metabolism-immunity axis as a possible therapy for AS.

## Introduction

Atherosclerosis (AS) is characterized by the accumulation of lipids in large and medium-sized arteries and the formation of immune cell-rich plaques. Advanced plaque may rupture or erode, resulting in thrombosis blocking arteries and obstructing blood flow, leading to a series of life-threatening clinical manifestations known as major adverse cardiovascular events (MACE) (1). Current therapies for AS, such as statins or serine protease PCSK9 inhibitors, are designed to control the levels of low-density lipoprotein (LDL) (2). However, although these therapies can effectively reduce LDL to the level recommended by the guidelines, the incidence of MACE is still higher than 50% (3). It is an urgent problem to find economical, safe and effective alternative drugs that can inhibit the occurrence and development of AS.

In recent years, it has been found that the composition of the gut microbiota plays an important role in the occurrence and development of AS, providing a new target for the prevention and treatment of AS (4, 5). Studies have confirmed that through the metabolism of dietary choline, the gut microbiota produce trimethylamine (TMA) (6), which is activated by flavin monooxygenase (FMO) to produce trimethylamine-*N*-oxide (TMAO). Healthy individuals have gut microbiota with high TMA and TMAO production, including genera from *Firmicutes*, *Proteobacteria*, and *Actinobacteria* (7). Some of these bacteria contain the cholintrimethylamine lyase (CutC) gene, which is a key target protein for phosphatidylcholine conversion to TMA (8). Therefore, some scholars have proposed that probiotics can be used to inhibit or block specific microbial metabolic pathways and reduce TMA-producing bacteria. For example, probiotics were administered to mice to regulate the metabolism of bile acids by changing the gut microbiota and reducing the risk of AS in the host (9). However, their specific immune and physiological effects on health and disease remain questionable and require further research (10).

Herbal medicines can prevent the phenotypic conversion of vascular smooth muscle cells (VSMCs) and inhibit endothelial dysfunction, platelet activation, lipid peroxidation, ROS production and macrophage-induced AS (11–13). β-Sitosterol is a natural active substance that widely exists in a variety of herbal medicines, and its chemical structure is similar to that of cholesterol, with an additional ethyl on C-24 (14). β-Sitosterol has been reported to be clinically effective in the treatment of diabetes, cardiovascular diseases (anti-thrombosis) and liver diseases (15–17). In terms of lipid regulation, β-sitosterol not only competes with cholesterol for binding sites, but also binds with cholecystokinin to induce gallbladder contraction to promote digestion and regulate gastric emptying (18). It could also reduce the absorption and reabsorption of cholesterol, bile acids and dietary lipids in non-alcoholic fatty liver mice induced by a high-fat diet and slow down the weight gain and accumulation of liver lipids (19). Gogoi et al. (16) found that β-sitosterol could exert anticoagulant effects through non-competitive inhibition of thrombin activity to cause thrombolysis and prevent stroke and venous thrombosis in mice. Recent studies have shown that β-sitosterol has the potential to inhibit peptidoglycan biosynthesis and prevent bacterial cell wall formation by inhibiting MurA and SrtA activities and regulating the oral flora (20). However, the effect of β-sitosterol on inhibiting TMA production by reshaping the gut microbiota and its mechanism have not yet been unveiled. The goal of this study was to determine whether β-sitosterol can inhibit choline metabolism *in vitro* and *in vivo* and reveal the detailed mechanism.

## Materials and methods

### Animals and treatments

Apolipoprotein E knockout mice (ApoE^–/–^ mice) were purchased from Cavins Experimental Animal Technology Co., Ltd. (Changzhou, China) and were kept in standard polypropylene cages at 22 ± 2°C and 55 ± 5% relative humidity with a 12-h light/dark period. The use of mice in this experiment was approved by the China Pharmaceutical University Animal Care and Use Committee, and the whole process of the animal experiments followed the guidelines of the Institute Animal Care and Use Committee of China Pharmaceutical University.

Twenty-four healthy ApoE^–/–^ mice (6 weeks, male) were adaptively fed for 3 days before the experiment. After fasting for 24 h, the mice were weighed and randomly divided into three groups with eight mice in each group. Nom group: ApoE^–/–^ mice fed a standard chow diet with 0.9% NaCl solution by gavage. Con group: AS model mice given a customized high-choline diet with 0.9% NaCl solution by gavage. Exp group: experimental mice given a customized high-choline diet with 400 mg/kg/d β-sitosterol (Plant Origin Biological, Nanjing, China) by gavage. All groups were given gavage once a day, their water was changed every 2 days, and their body weight and food intake were weighed and recorded weekly. Eight weeks later, the mice were anesthetized with 50 mg/kg pentobarbital sodium, and serum and liver samples were collected and stored at −80°C for future use. The liver index of the mice was calculated (liver index = liver weight/mouse body weight). Samples of heart tissue and colon contents were taken for subsequent experiments.

### Atherosclerotic plaque analysis

Atherosclerotic plaques were analyzed in the aortas by Oil Red O (ORO) staining (21). Heart tissue samples were collected and stored in 4% paraformaldehyde. After dehydration, the heart with the ascending aorta was cut below the aortic root and placed in an embedding machine until it was completely solidified. The aortic sinus was sectioned into serial 10 μm slices, which were stained with ORO and observed under a microscope with an HS6 pathological section scanner to obtain pathological image information (the atherosclerotic plaques were stained red).

### Cholesterol and choline metabolism analysis

Enzyme-linked immunosorbent assay (ELISA) kits were used according to the manufacturer’s instructions (Jiancheng Bioengineering Institute, Nanjing, China) to detect the serum levels of TMA, TMAO, total cholesterol (TC), TG, low-density lipoprotein cholesterol (LDL-C), and high-density lipoprotein cholesterol (HDL-C) and the liver level of FMO3.

### Proinflammatory cytokines and antioxidant activity analysis

Proinflammatory cytokines in the serum were detected by real-time fluorescence quantitative PCR on a QuantStudio 3 Real-Time qPCR amplifier. The sequences of the primers used in this study was given in Table 1. β-actin was used as a reference gene. The specificity of the primers was verified by fusion curve analysis. The expression abundance of related genes was determined by the Ct value, and the relative gene expression was calculated by 2^–ΔΔ*Ct*^.

**TABLE 1:** Primers used in this study.

Gene	Primer sequence (5′-3′)
IL-1β	FOR: GGTCAAAGGTTTGGAAGCAG REV: TGTGAAATGCCACCTTTTGA
IL-6	FOR: AGGGTCTGGGCCATAGAACT REV: CCACCACGCTCTTCTGTCTAC
TNF-α	FOR: AGGGTCTGGGCCATAGAACT REV: CCACCACGCTCTTCTGTCTAC
β-actin	FOR: GCTGTGCTATGTTGCTCTAG REV: CGCTCGTTGCCAATAGTG

The glutathione peroxidase (GSH-Px), superoxide dismutase (SOD) activities and malondialdehyde (MDA) levels in the serum were assayed using the respective commercial kits according to the manufacturer’s instructions (Jiancheng Bioengineering Institute, Nanjing, China).

### 16S rRNA sequencing and microbial diversity analysis

Bacterial 16S rRNA gene sequencing assays were performed by Ling En Biotechnology Co., Ltd. (Shanghai, China). The amplicons were standardized, collected and sequenced. The UCHIME algorithm was used to remove all chimeric labels and obtain valid labels for further analysis. The effective marker clusters were counted as operational taxonomic units (OTUs), and the similarity threshold was ≥ 97%. The representative sequence of each OTU was selected and the classification information was annotated using the RDP classifier and SILVA database. Analysis was performed at each taxonomic level (phylum, class, order, family, genus). Alpha and beta diversity analyses were performed based on the output normalized data. Principal component analysis (PCA) and principal coordinate analysis (PCoA) were performed to compare the microbial composition between the groups. Finally, the significant differential abundant taxonomy between different groups was evaluated by the linear discriminant analysis effect size (LEfSe) method and Wilcoxon rank-sum test.

### The effect of β-sitosterol on the trimethylamine production of intestinal bacteria *in vitro*

The TMA-producing intestinal bacteria (KT-1) isolated in our previous research (22) were cultured in nutrient broth (NB) containing 20 mM choline under anaerobic conditions in a 37°C shaker incubator (150 rpm). According to the MIC in our previous research, appropriate concentrations (20 μg/ml, 2 μg/ml) were selected for use and subjected to sterilization by filtration. The TMA concentrations in the 12, 24, and 36 h cultures were measured by the picric acid-toluene method (23).

### Statistical analysis

All data are presented as the mean ± SD. Values of *p < 0.05, ^**^p < 0.01 and ^***^p < 0.001 indicate significant differences. Comparisons between two groups were analyzed by two-tailed Student’s t-test using GraphPad Prism 9 (GraphPad Software, La Jolla, CA, USA).

## Results

### β-sitosterol modulated cholesterol metabolism in atherosclerosis mice

**FIGURE 1:**
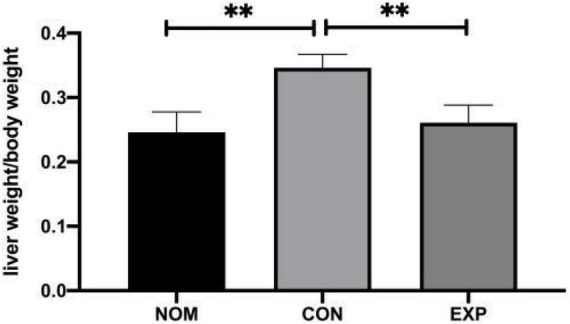
β-sitosterol lowers the liver index of atherosclerosis (AS) mice. Values are presented as the mean ± SD. ^**^p < 0.01.

**FIGURE 2:**
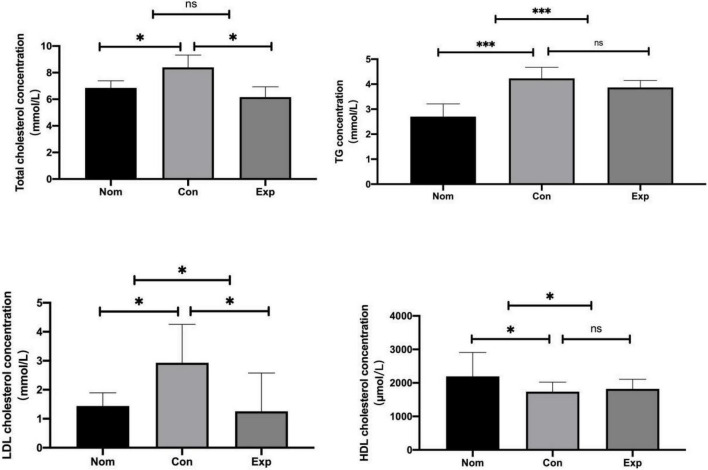
β-sitosterol modulated lipid metabolism in atherosclerosis (AS) mice. Values are presented as the mean ± SD. *p < 0.05; ^***^p < 0.001, ns = not significant.

There was no significant difference in feed intake among all groups. The liver index increased in the Con group compared with the Nom group, but decreased significantly in the Exp group with β-sitosterol treatment (Figure 1). Compared with the Nom group, TC, TG, and LDL-C in the Con group were significantly increased, while HDL was significantly decreased. After β-sitosterol treatment, TC and LDL-C in the Exp group were significantly decreased to normalcy (Figure 2). However, there were no significant difference in TG and HDL levels between Con and Exp groups.

### β-sitosterol protected ApoE^–/–^ mice from choline-induced atherosclerosis

**FIGURE 3:**
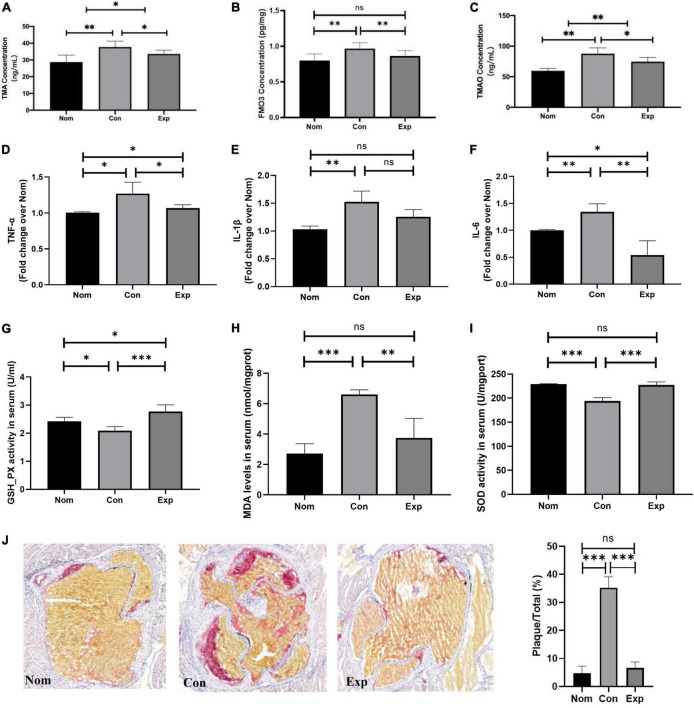
β-sitosterol protected ApoE^–/–^ mice from choline-induced atherosclerosis (AS). **(A–C)** β-Sitosterol modulated choline metabolism. **(D–I)** β-sitosterol reduced proinflammatory cytokines and antioxidant activity. **(J)** β-sitosterol reversed the atherosclerotic lesions. The aortic root was assessed by Oil Red O staining, and the plaques area were quantified by ImageJ. Values are presented as the mean ± SD. *p < 0.05; ^**^p < 0.01; ^***^p < 0.001, ns = not significant.

To further investigate whether β-sitosterol inhibits the excessive TMAO formation, we detected the FMO3 level in the liver and TMA and TMAO concentrations in the serum. As shown in Figures 3A–C, compared with the Con group, β-sitosterol significantly decreased the levels of TMA, FMO3 and TMAO in the Exp group. In addition, proinflammatory cytokines (TNF-α and IL-6) (Figures 3D,F), antioxidant activity (GSH-Px and SOD) (Figures 3G,I) and a marker of lipid peroxidation (MDA) (Figure 3H) were reversed by β-sitosterol treatment of AS mice. No significant difference in serum IL-β level was observed between the Con and Exp groups (Figure 3E).

In addition, we further investigated whether β-sitosterol relieved AS in ApoE^–/–^ mice. The atherosclerotic plaques were visualized by ORO staining. As shown in Figure 3J, choline markedly enhanced the atherosclerotic plaques in the Con group, and this effect was reversed by β-sitosterol treatment in the Exp group.

### β-sitosterol decreased trimethylamine production by remodeling the gut microbiota in ApoE^–/–^ mice

To investigate whether TMA inhibition by β-sitosterol under choline conditions is mediated by gut microbial remodeling, we first explored bacterial populations in the cecal content samples from the three groups of mice (Nom, Con, Exp) by 16S rRNA gene sequence analysis. The α-diversity indices displayed in Table 2 were used to evaluate the microbiota community in the three groups. The Shannon and Simpson indices reflect the diversity of the intestinal microbiota, while the Chao 1 index reflects the species richness. Our results showed that the abundance and diversity of intestinal bacteria in mice were not affected by a high-choline diet (Con group). Notably, β-sitosterol treatment significantly increased the abundance and diversity of intestinal bacteria in Exp group mice.

**TABLE 2:** Analysis of alpha diversity indices.

Index	Con	Nom	Exp
Chao1	640.207	660.887 (*p* = 0.679)	898.789 (*p* = 0.009)
Shannon	3.987	4.085 (*p* = 0.780)	5.064 (*p* = 0.003)
Simpson	0.165	0.182 (*p* = 0.640)	0.029 (*p* = 0.029)

**FIGURE 4:**
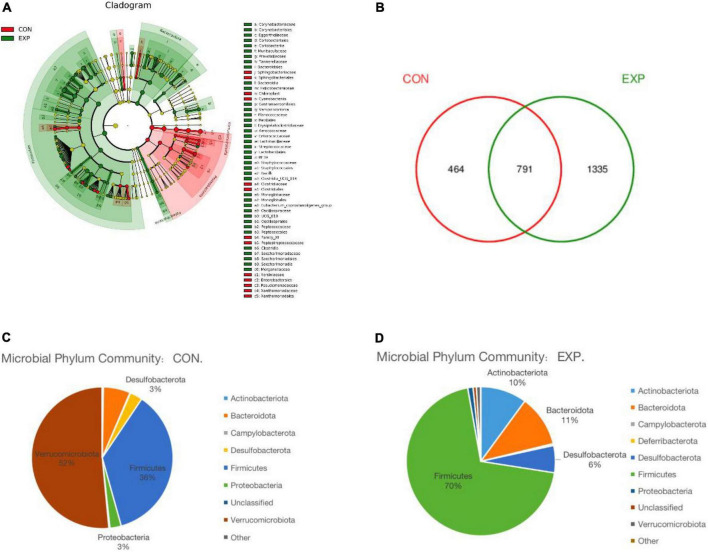
**(A)** Linear discriminant analysis effect size (LEfSe) comparison of gut microbiota between the Con and Exp groups. **(B)** Venn diagrams showing the shared operational taxonomic units (OTUs) between the different groups. **(C,D)** The taxonomic profile of the Con and Exp groups.

Linear discriminant analysis effect size analysis was performed to identify the distinct bacterial species between the Con and Exp groups (Figure 4A). The Venn diagram reflecting the difference between the two groups, as shown in Figure 4B, exhibited 464 and 1,335 in the Con and Exp groups, respectively. At the phylum level, compared with the Con group, the Exp group showed a higher abundance of *Actinobacteriota*, *Bacteroidota*, *Desulfobacterota*, and *Firmicutes* and a lower abundance of *Proteobacteria* and *Verrucomicrobiota* (Figures 4C,D). The Wilcoxon rank-sum test was used to highlight the OTU abundance differences between the two groups. The results showed that the intestinal microbes of the two groups were mainly different in *Firmicutes*, *Verrucomicrobiota*, *Patescibacteria*, *Proteobacteria*, and *Bacteroidota* (Table 3).

**TABLE 3:** The operational taxonomic unit (OTU) abundance differences at the phylum level.

Phylum	Con		Exp		*P*-value
	Mean	SD	Mean	SD	
*Firmicutes*	0.36305	0.13071	0.76941	0.12289	0.005
*Patescibacteria*	0.00003	0.00003	0.00030	0.00027	0.018
*Bacteroidota*	0.06172	0.04656	0.12282	0.05731	0.045
*Proteobacteria*	0.02601	0.00323	0.01265	0.00790	0.020
*Verrucomicrobiota*	0.51346	0.12845	0.00993	0.00348	0.005

**FIGURE 5:**
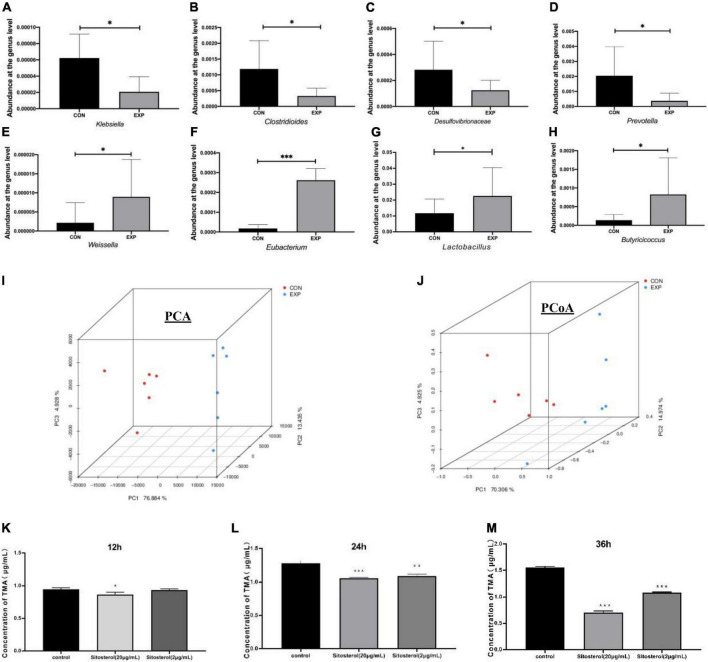
β-sitosterol decreased trimethylamine (TMA) production by remodeling the gut microbiota in ApoE^− /−^ mice **(A–H)** comparison of abundant components of the microbiome at the family level between the Con and Exp groups (*n* = 6 for each group). **(I)** Principal component analysis (PC1 76.88% vs. PC2 13.44%). **(J)** Principal coordinates analysis (PCoA1 70.31% vs. PCoA2 14.97%). **(K–M)** TMA concentrations at 12, 24, and 36 h of β-sitosterol treated groups in the TMA-producing strain. Values are presented as the mean ± SD. *p < 0.05; ^**^p < 0.01; ^***^p < 0.001.

Genus-level characterizations were identified between the Con and Exp groups (Figures 5A–H). Compared with the Con group, the abundance of *Klebsiella*, *Clostridioides*, *Desulfovibrionaceae*, and *Prevotella* were decreased, while *Weissella*, *Eubacterium*, *Lactobacillus*, and *Butyricioccus* were markedly enriched by β-sitosterol treatment. The PCA and PCoA analysis showed that the gut microbiome of the Con group clustered significantly separately from that of the Exp group (Figures 5I,J). Meanwhile, β-sitosterol can significantly inhibit gut flora generating TMA from choline *in vitro* (Figures 5K–M). These results showed that the intestinal flora played a key role in the inhibition of TMA production by β-sitosterol.

## Discussion

A number of studies have shown a causal correlation between TMAO and AS in mouse models (24–26), and small molecule inhibitors of gut flora choline-TMA lyase have been reported to be effective against choline diet-induced AS (27, 28). Here, this study provides the first evidence that β-sitosterol inhibited TMA production in ApoE^–/–^ mice by reshaping the gut microbial structure and attenuated atherosclerosis. We also found that β-sitosterol could alleviate cholesterol metabolism and the inflammatory response, and improve the antioxidant defense capacity.

It has been reported that Chinese yam extracts containing β-sitosterol could reduce plasma concentrations of total cholesterol in ApoE^–/–^ mice (29). Therefore, to study the involvement of β-sitosterol in the choline-TMA-TMAO pathway, mice were fed a choline supplemented diet to avoid the interference of cholesterol metabolism. Our results showed that β-sitosterol effectively reduced the TMA, FMO3, and TMAO levels while ameliorating the atherosclerotic plaques of AS mice. Meanwhile, β-sitosterol could significantly inhibit gut flora generating TMA from choline *in vitro* and modulate the structure of the gut microbiota *in vivo*. This is consistent with some previous reports of β-sitosterol in rumen acidosis of sheep or polycystic ovary syndrome-like mice (30, 31).

As predicted, 16S rRNA sequencing of the gut microbiota showed significant differences in both alpha and beta diversity between the Con and Exp groups. Moreover, the results of LEfSe analysis at the OTU level indicated that the abundance of some bacterial communities changed significantly in response to β-sitosterol treatment. In the β-sitosterol treatment group (Exp group), the relative abundance of *Actinobacteriota*, *Bacteroidota*, and *Firmicutes* in the intestinal tract was significantly increased. These bacteria have pathways associated with the metabolism of carbohydrates, bile acids and steroids (32). In addition, genera with decreased OTUs were assigned to *Klebsiella*, *Clostridioides*, *Desulfovibrionaceae*, and *Prevotella*, which contain the gene cutC, responsible for the major pathway for TMA production (7). These results suggested that β-sitosterol has beneficial effects on intestinal flora function, especially an inhibitory effect on TMA production. Similar to a previous report about the effect of berberine on intestinal flora construction (33), *Weissella*, *Eubacterium*, *Lactobacillus*, and *Butyricioccus* were markedly enriched by β-sitosterol treatment. Feng et al. (34) found that these bacteria produced short-chain fatty acids (SCFAs), such as acetate, n-propionate and n-butyrate. Whether changes of SCFAs are involved in the role of β-sitosterol in AS mice remains to be investigated in future studies.

At present, the mechanism by which TMAO affects AS is not completely clear, but it may involve the following pathways. (1) Cholesterol metabolism and TMAO. TMAO promotes the accumulation of cholesterol in macrophages depending on the gut microbiota (35, 36). In addition, TMAO is believed to participate in the uptake of oxidized LDL (37, 38), which induces the formation of foam cells and triggers AS. Consistent with these findings, the present data showed that TC and LDL-C were significantly decreased to normalcy by β-sitosterol treatment. (2) Inflammation and TMAO. Inflammation is closely related to the development of AS. TMAO may disrupt endothelial and VSMCs function and promote inflammation (14, 39). Among the various inflammatory factors and chemokines, TNF-α plays a powerful role in promoting AS by increasing the permeability of endothelial cells and stimulating the activation of macrophages (40). IL-1β and IL-6 are the major proinflammatory factors in AS that promote the formation of fatty streaks in blood vessel walls by aggravating the subsequent inflammatory cascade and mediating the acute phase reaction (41). Our data were in agreement with the study of Jayaraman et al. who reported that β-sitosterol restored the elevated serum levels of proinflammatory cytokines, including TNF-α, IL-6, and leptin, to normal levels in type 2 diabetic rats (15). The results of our study suggested that β-sitosterol could alleviate the inflammatory response in AS mice. (3) Oxidative stress and TMAO. Oxidative stress is a redox imbalance with increased ROS production and overloaded antioxidant defense capacity. Severe oxidative stress can lead to AS (42). Endogenous antioxidant enzymes, including SOD and GSH-Px, maintain the redox equilibrium by removing excess ROS (43). Previous studies showed that TMAO inhibited SOD and GSH-Px activation and increased the plasma level of MDA (24, 44, 45). Similarly, weakened antioxidant defenses and exacerbated oxidative stress were observed in Con group mice with TMAO accumulation. Together with the finding that β-sitosterol could improve the expression of SOD and GSH-Px and inhibit the formation of MDA in the Exp group, the present data clearly showed that β-sitosterol possessed antioxidant capacity and antiatherogenic activity in mice.

In summary, these results demonstrated that β-sitosterol could inhibit trimethylamine production, attenuate AS, alleviate the inflammatory response and improve antioxidant defense capacity. Besides, β-sitosterol has beneficial effects on gut microbial communities, which is responsible for the major pathway for TMA production. Therefore, it was concluded that the microbiota-metabolism-immunity axis might play a pivotal role in the therapeutic mechanism of β-sitosterol. Our work can provide new insights into understanding how β-sitosterol prevents and treats AS. Further investigation will be needed to clarify the effect of β-sitosterol on AS in humans.

## Data availability statement

The original research data presented in this study are included in the Supplementary material.

## Ethics statement

This animal study was reviewed and approved by China Pharmaceutical University Animal Care and Use Committee.

## Author contributions

JJ and WC designed the experiments and performed revisions of the manuscript. WPW conducted the animal experiments and sample analyses. WL and WW assisted the experiments. WPW and HW wrote the draft of the manuscript. All authors contributed to the article and approved the submitted version.
