# Supplementary figures and images for: β-sitosterol inhibits trimethylamine production by regulating the gut microbiota and attenuates atherosclerosis in ApoE–/– mice
Source: Front Cardiovasc Med. 2022 Nov 1;9:986905. doi: 10.3389/fcvm.2022.986905 (PMC9663806; doi:10.3389/fcvm.2022.986905)

## Sequence length distribution

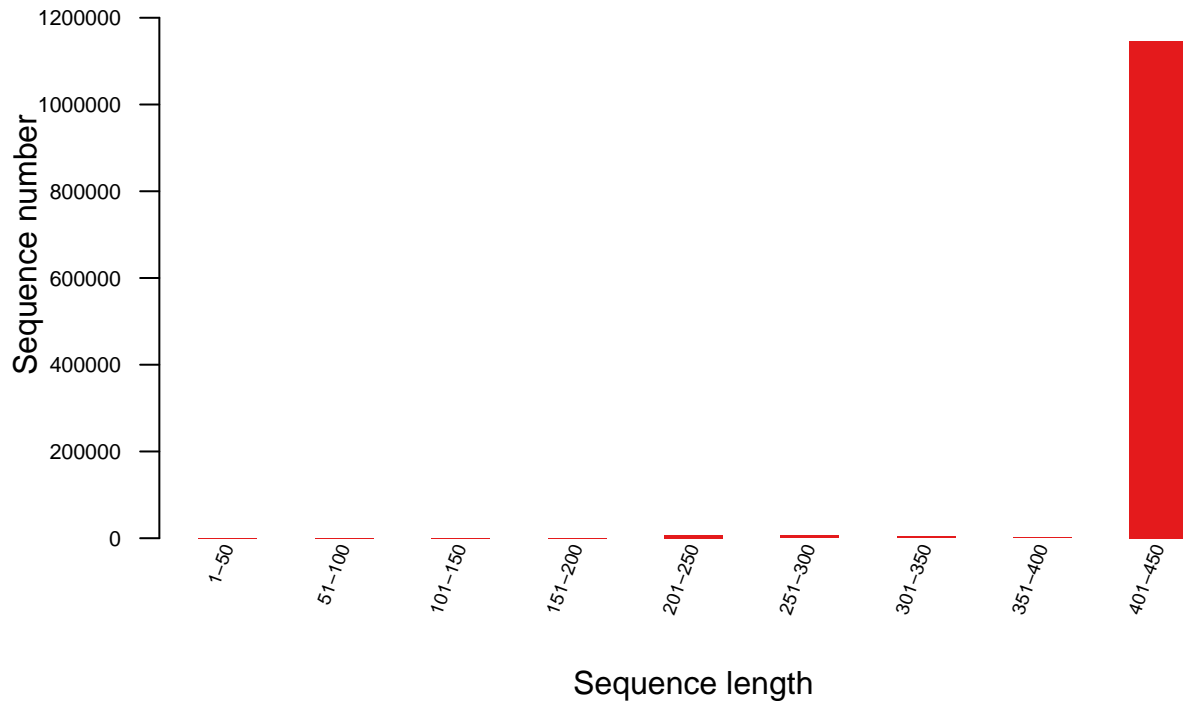

Supplement: Supplementary file 1 [file Data_Sheet_1.ZIP › Source data/gut microbiota diversity analysis/CON-EXP/00.Data/clean.data.len.pdf]

ACE(p=0.01631)

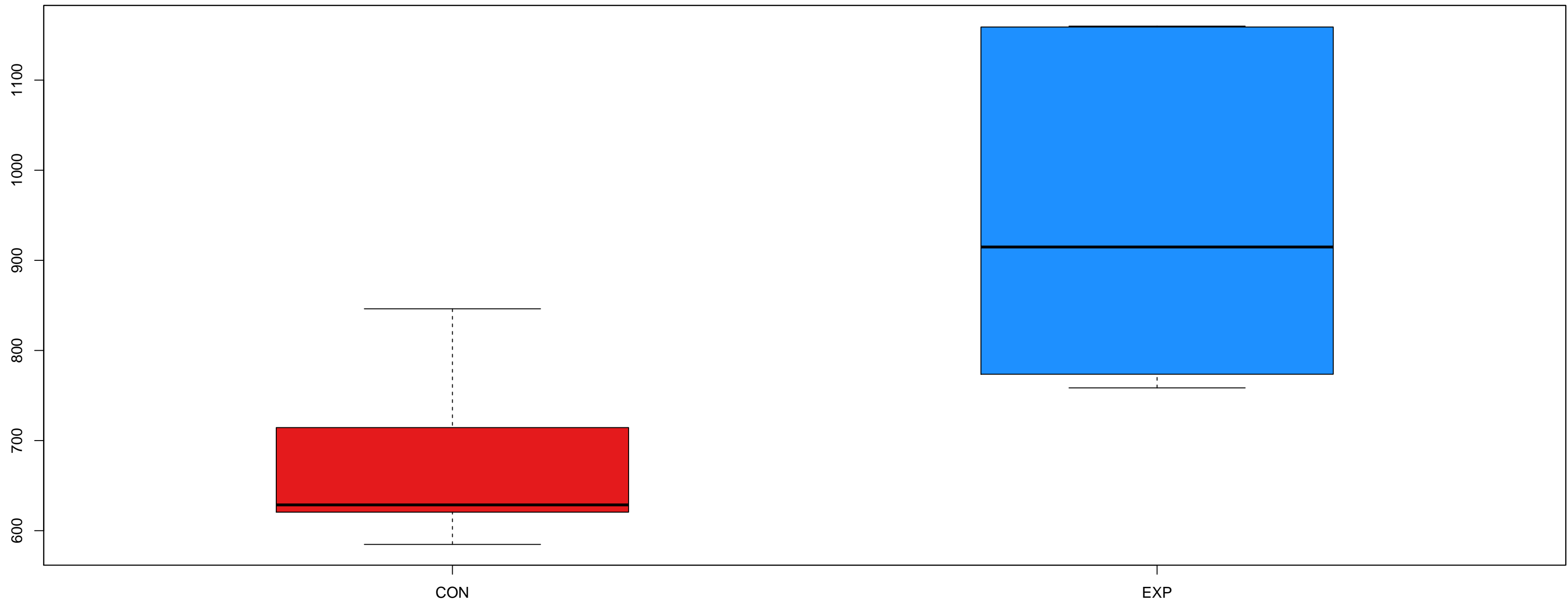

Supplement: Supplementary file 1 [file Data_Sheet_1.ZIP › Source data/gut microbiota diversity analysis/CON-EXP/02.Alpha/01.Estimators/ACE.group.boxplot.pdf]

Chao(p=0.01631)

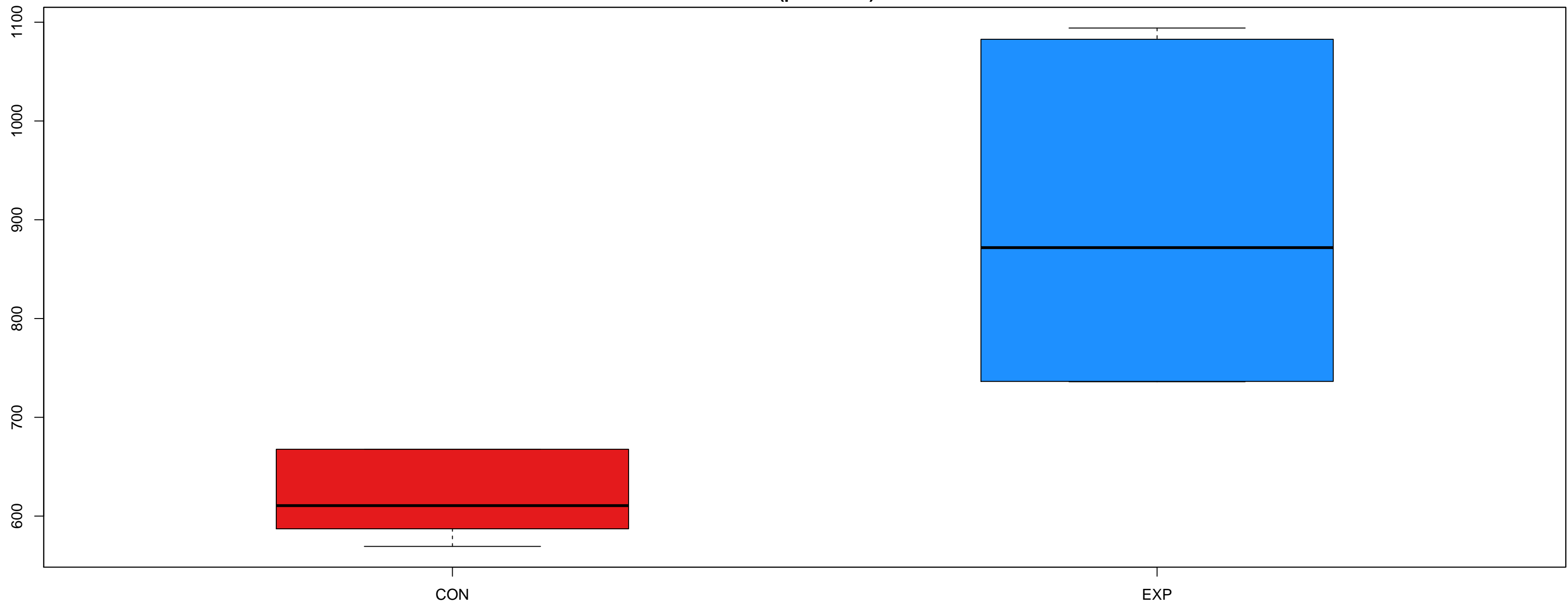

Supplement: Supplementary file 1 [file Data_Sheet_1.ZIP › Source data/gut microbiota diversity analysis/CON-EXP/02.Alpha/01.Estimators/Chao.group.boxplot.pdf]

Evenness(p=0.03737)

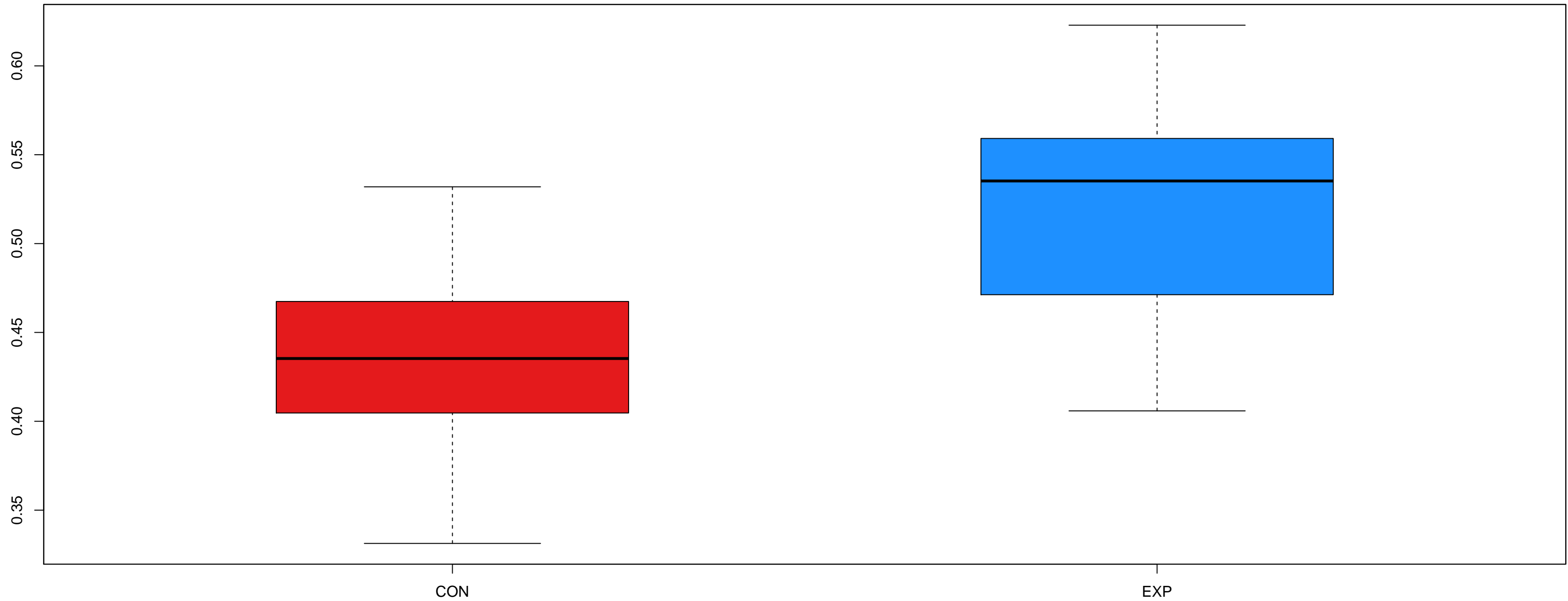

Supplement: Supplementary file 1 [file Data_Sheet_1.ZIP › Source data/gut microbiota diversity analysis/CON-EXP/02.Alpha/01.Estimators/Evenness.group.boxplot.pdf]

Reads(p=0.07817)

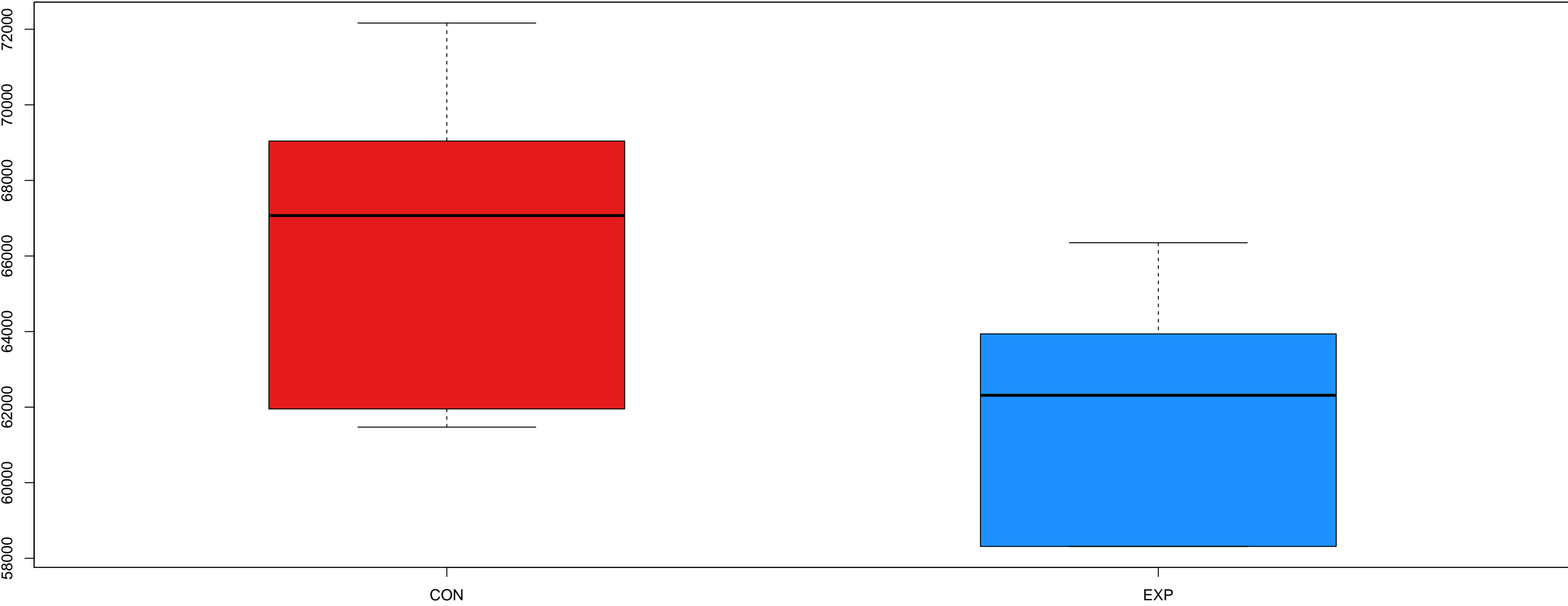

Supplement: Supplementary file 1 [file Data_Sheet_1.ZIP › Source data/gut microbiota diversity analysis/CON-EXP/02.Alpha/01.Estimators/Reads.group.boxplot.pdf]

Richness(p=0.01612)

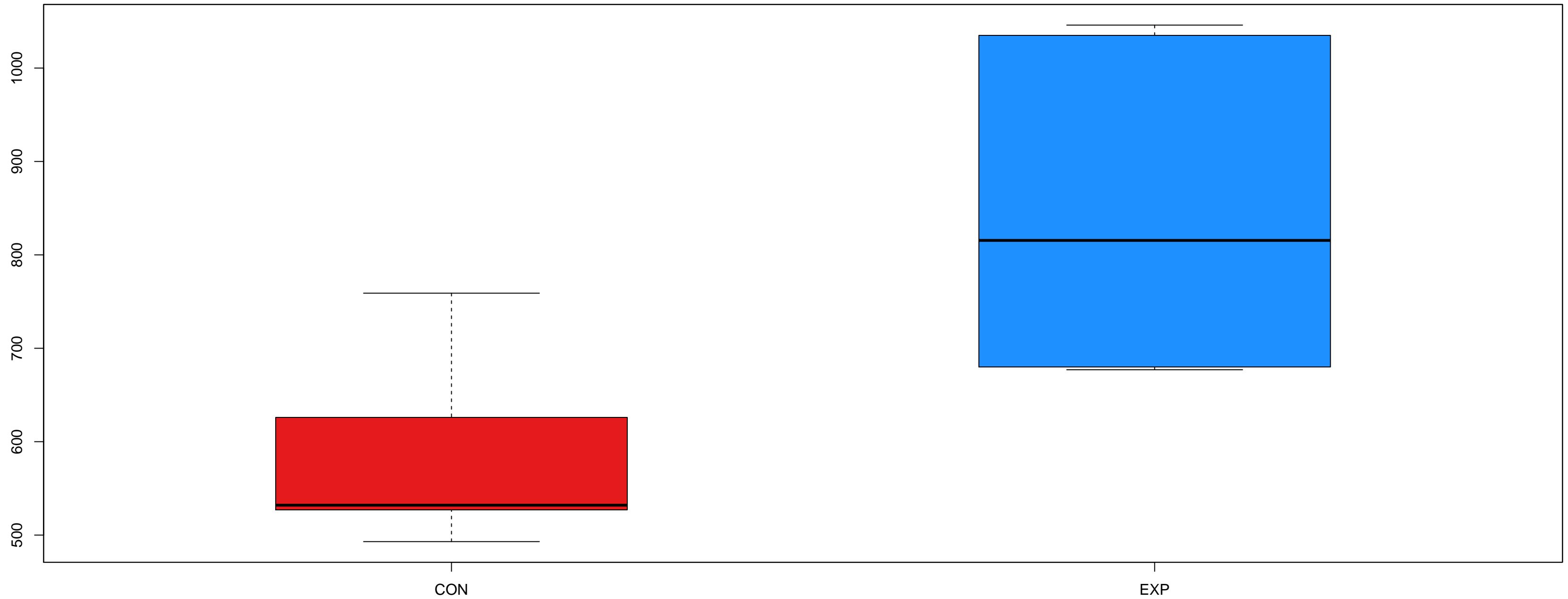

Supplement: Supplementary file 1 [file Data_Sheet_1.ZIP › Source data/gut microbiota diversity analysis/CON-EXP/02.Alpha/01.Estimators/Richness.group.boxplot.pdf]

Shannon(p=0.05466)

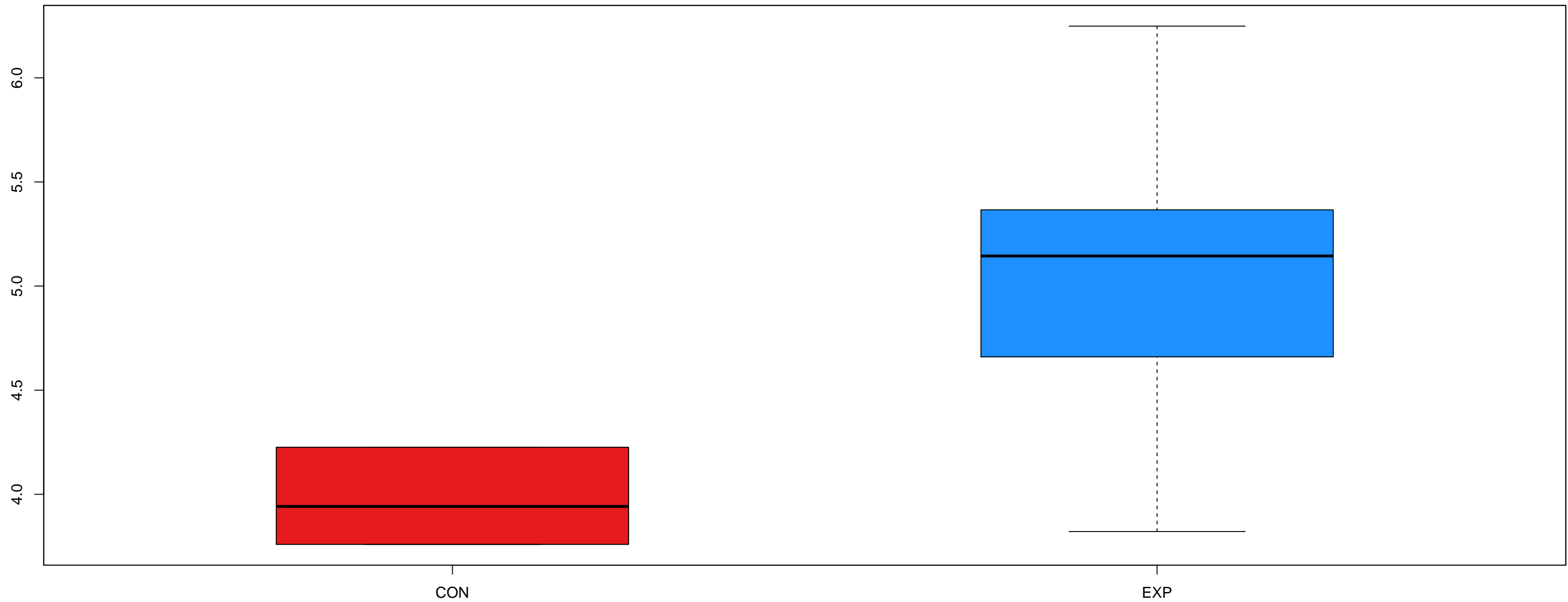

Supplement: Supplementary file 1 [file Data_Sheet_1.ZIP › Source data/gut microbiota diversity analysis/CON-EXP/02.Alpha/01.Estimators/Shannon.group.boxplot.pdf]

Simpson(p=0.05466)

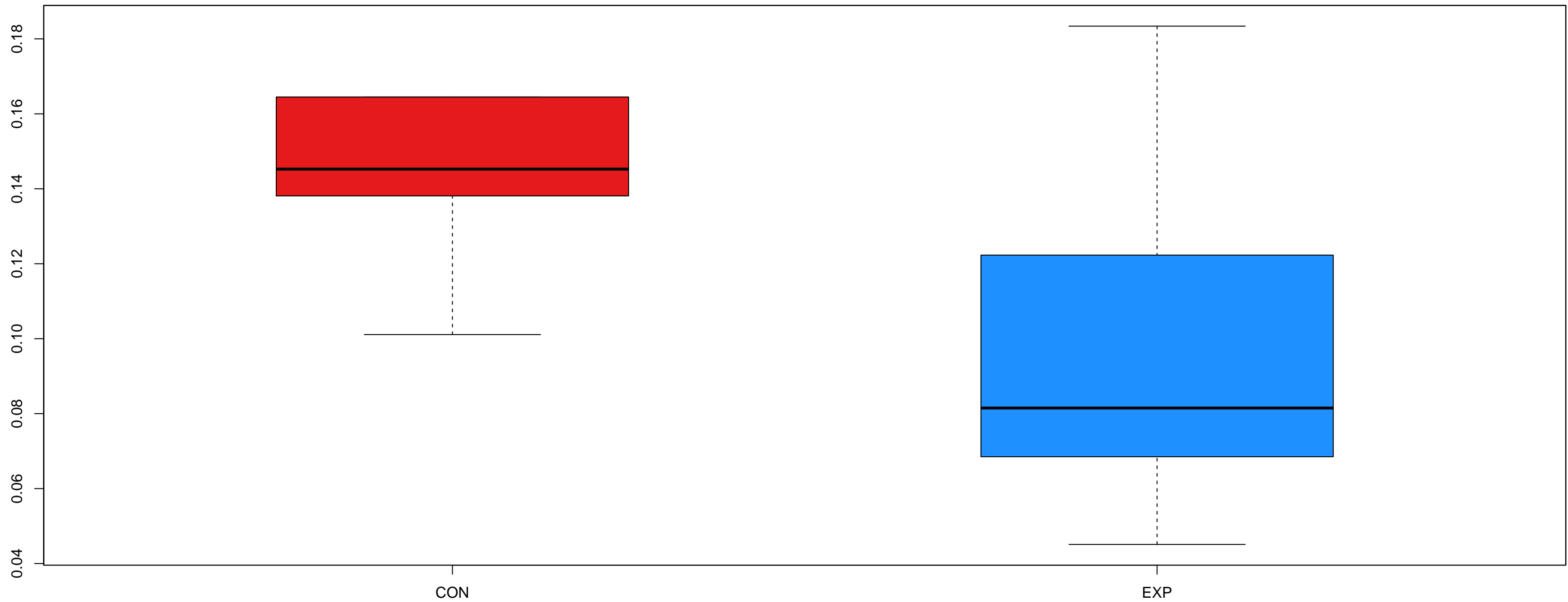

Supplement: Supplementary file 1 [file Data_Sheet_1.ZIP › Source data/gut microbiota diversity analysis/CON-EXP/02.Alpha/01.Estimators/Simpson.group.boxplot.pdf]

coverage(p=0.03737)

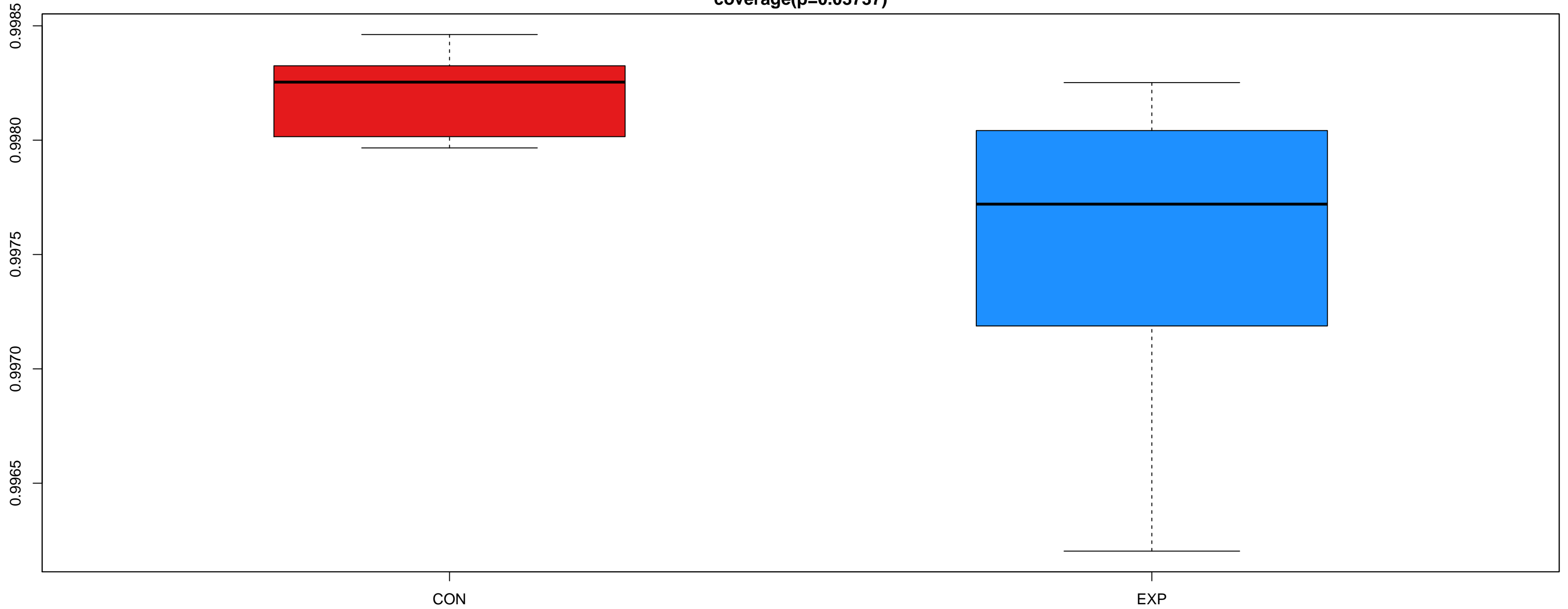

Supplement: Supplementary file 1 [file Data_Sheet_1.ZIP › Source data/gut microbiota diversity analysis/CON-EXP/02.Alpha/01.Estimators/coverage.group.boxplot.pdf]

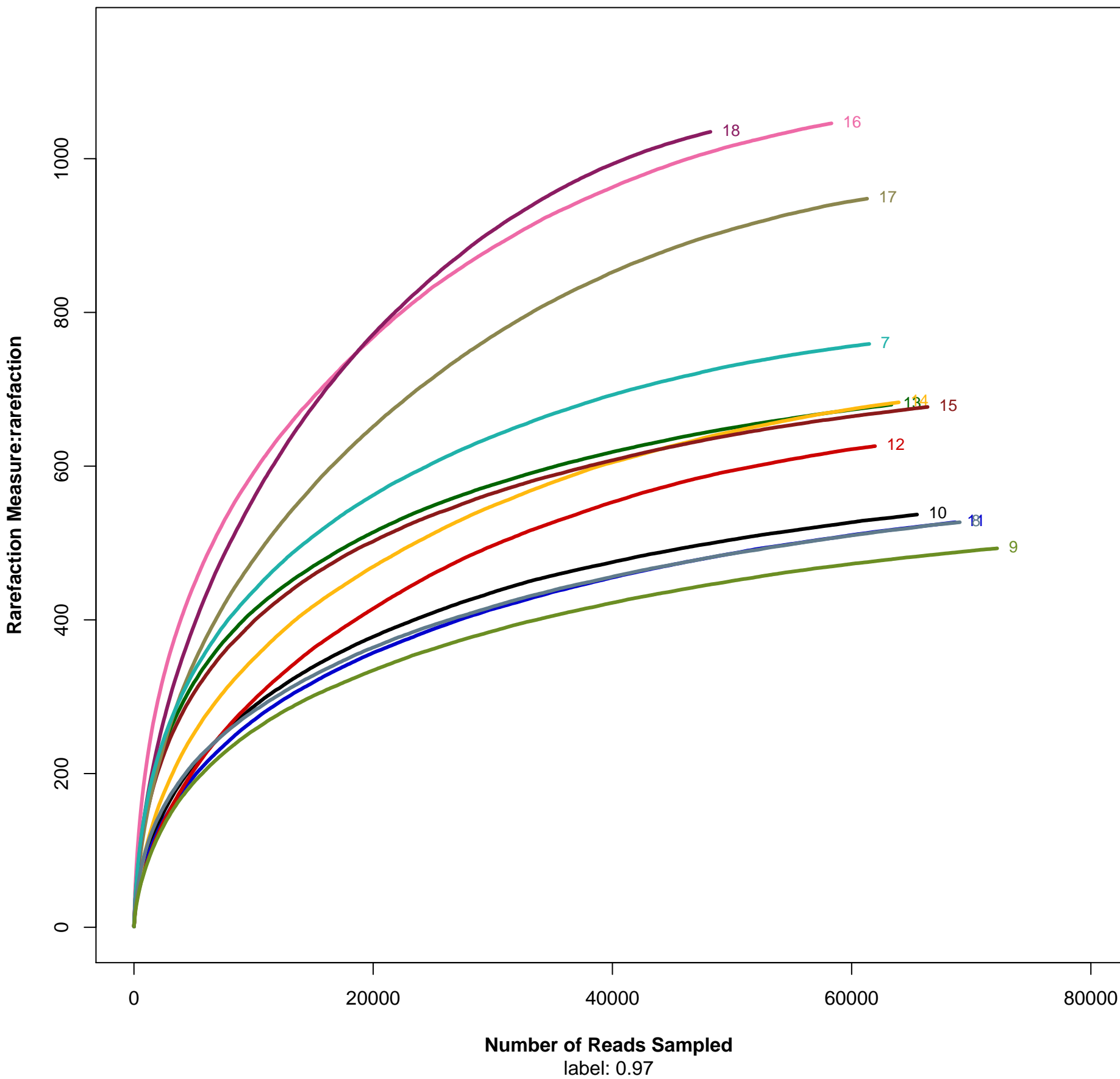

Supplement: Supplementary file 1 [file Data_Sheet_1.ZIP › Source data/gut microbiota diversity analysis/CON-EXP/02.Alpha/02.Rarefaction/rarefaction.All.pdf]

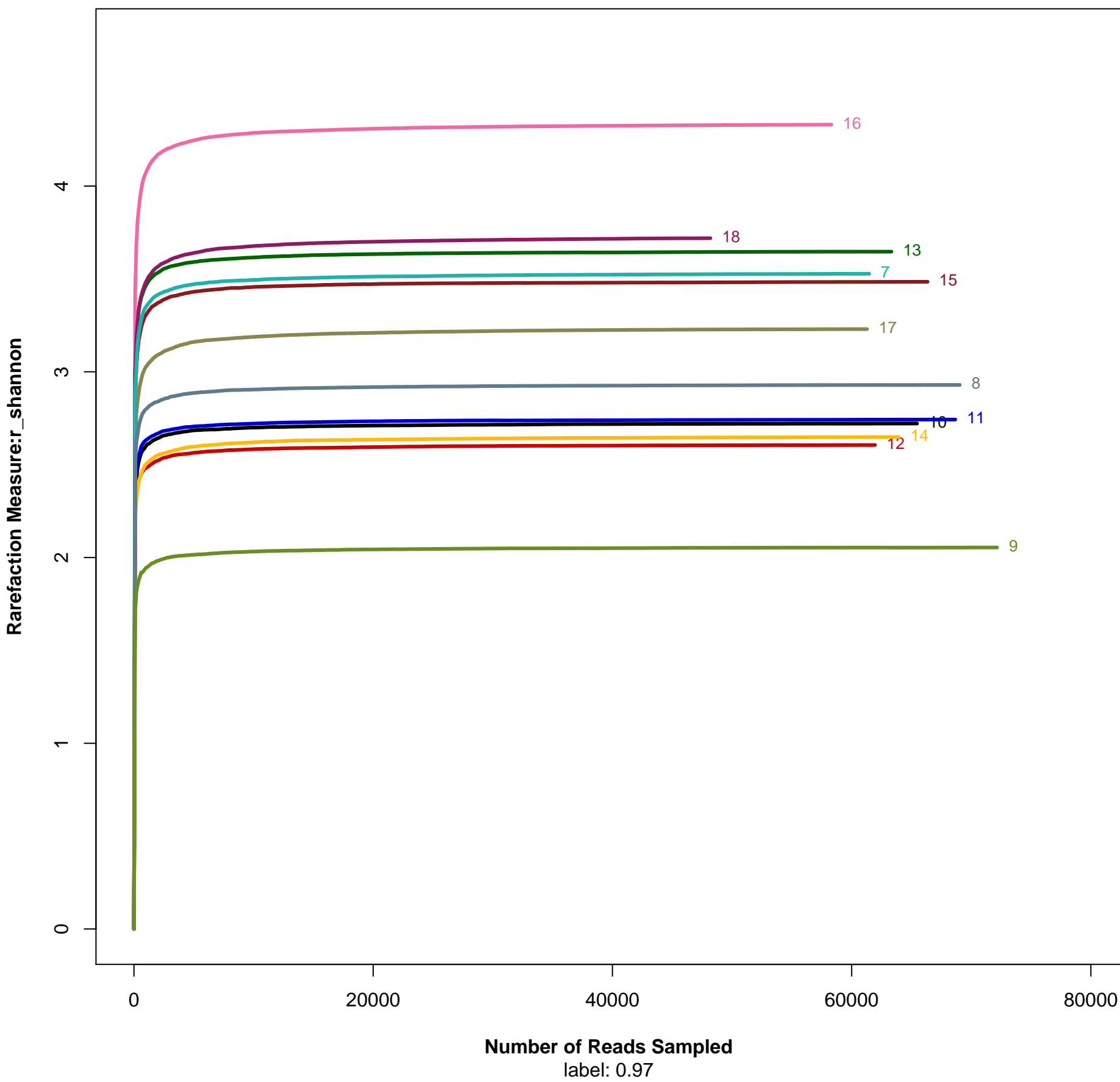

Supplement: Supplementary file 1 [file Data_Sheet_1.ZIP › Source data/gut microbiota diversity analysis/CON-EXP/02.Alpha/03.Shannon_rarefac/r_shannon.All.pdf]

**Rank–abundance distribution curve**

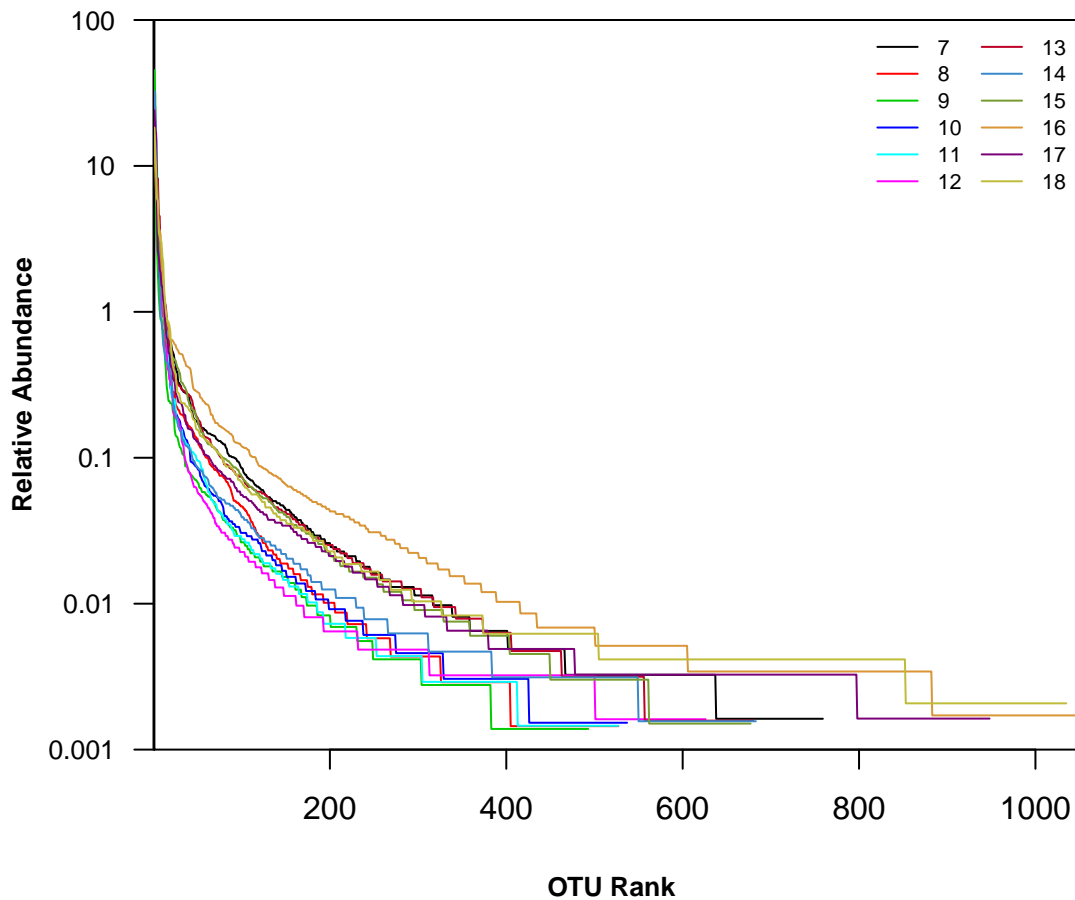

Supplement: Supplementary file 1 [file Data_Sheet_1.ZIP › Source data/gut microbiota diversity analysis/CON-EXP/02.Alpha/04.Rank_abundance/rankabundance.pdf]

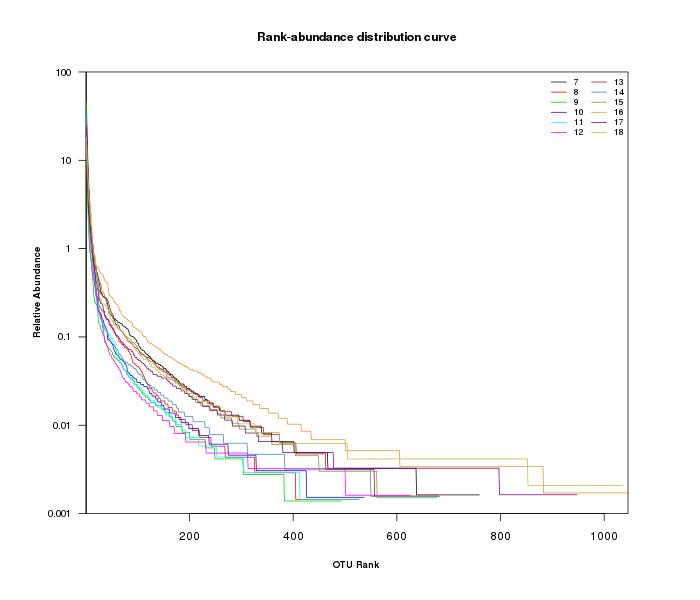

Supplement: Supplementary file 1 [file Data_Sheet_1.ZIP › Source data/gut microbiota diversity analysis/CON-EXP/02.Alpha/04.Rank_abundance/rankabundance.png]

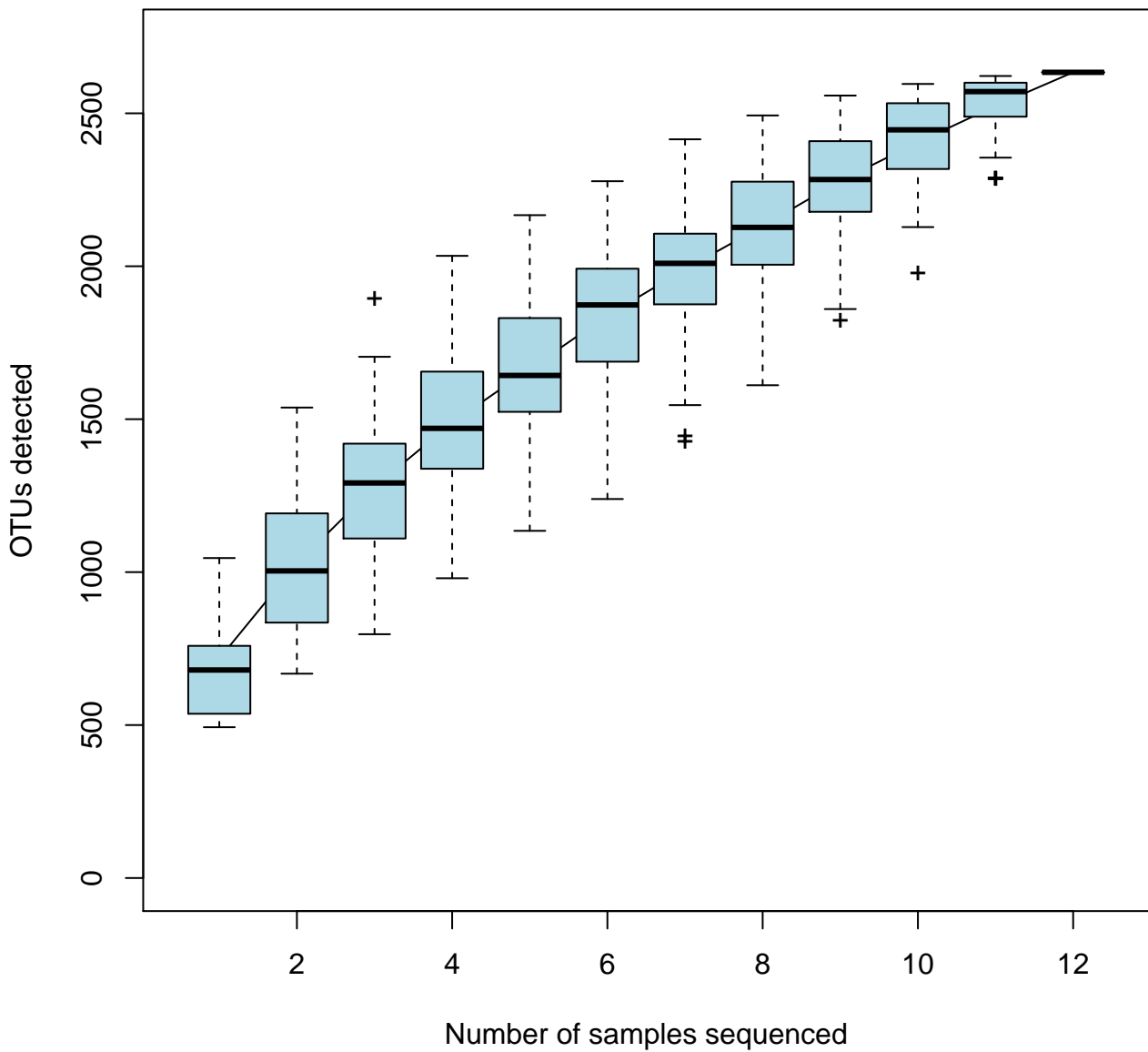

Supplement: Supplementary file 1 [file Data_Sheet_1.ZIP › Source data/gut microbiota diversity analysis/CON-EXP/02.Alpha/05.Specaccum/specaccum.all.pdf]

CON

EXP

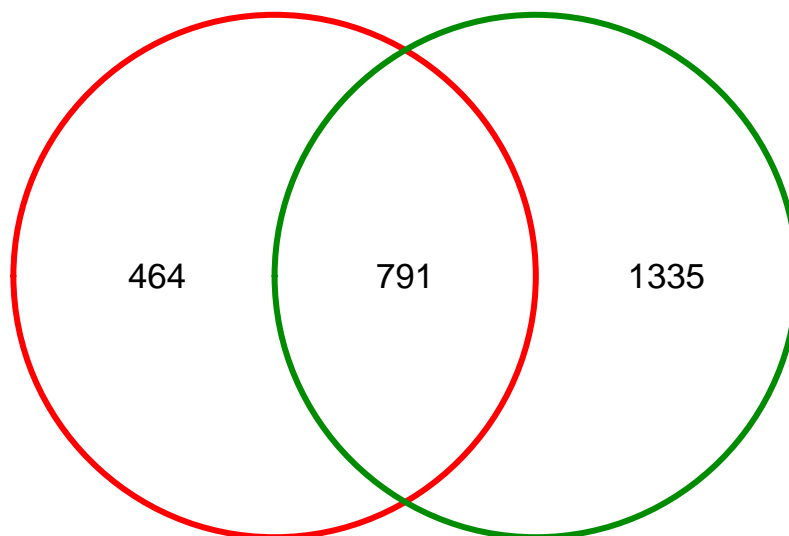

Supplement: Supplementary file 1 [file Data_Sheet_1.ZIP › Source data/gut microbiota diversity analysis/CON-EXP/03.Community_composition/01.Venn/venn.pdf]

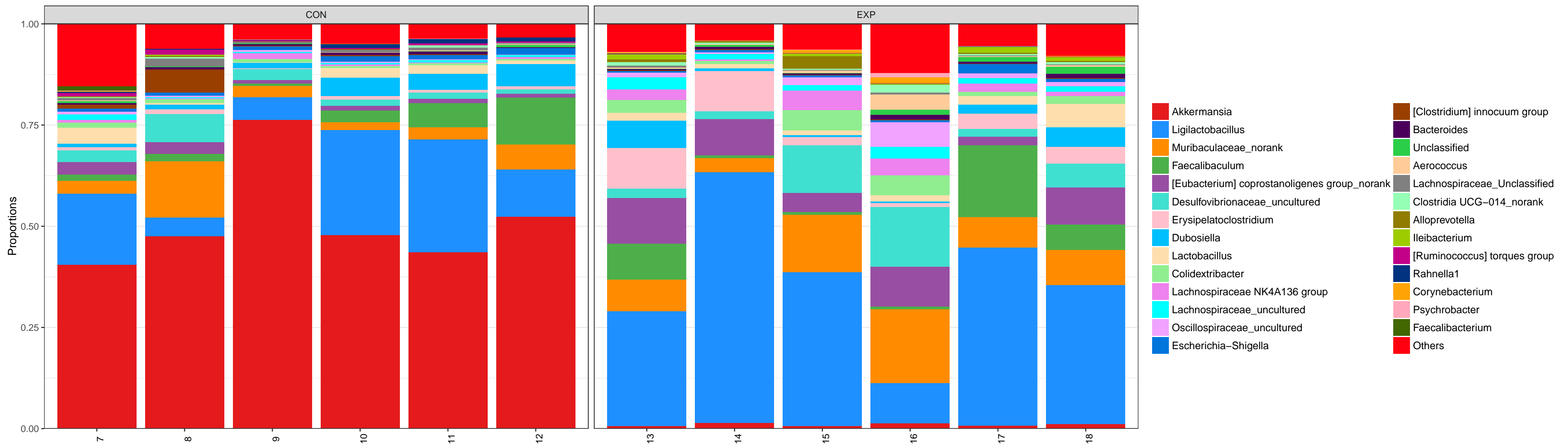

Supplement: Supplementary file 1 [file Data_Sheet_1.ZIP › Source data/gut microbiota diversity analysis/CON-EXP/03.Community_composition/02.Community/bar.ALL.genus.xls.pdf]

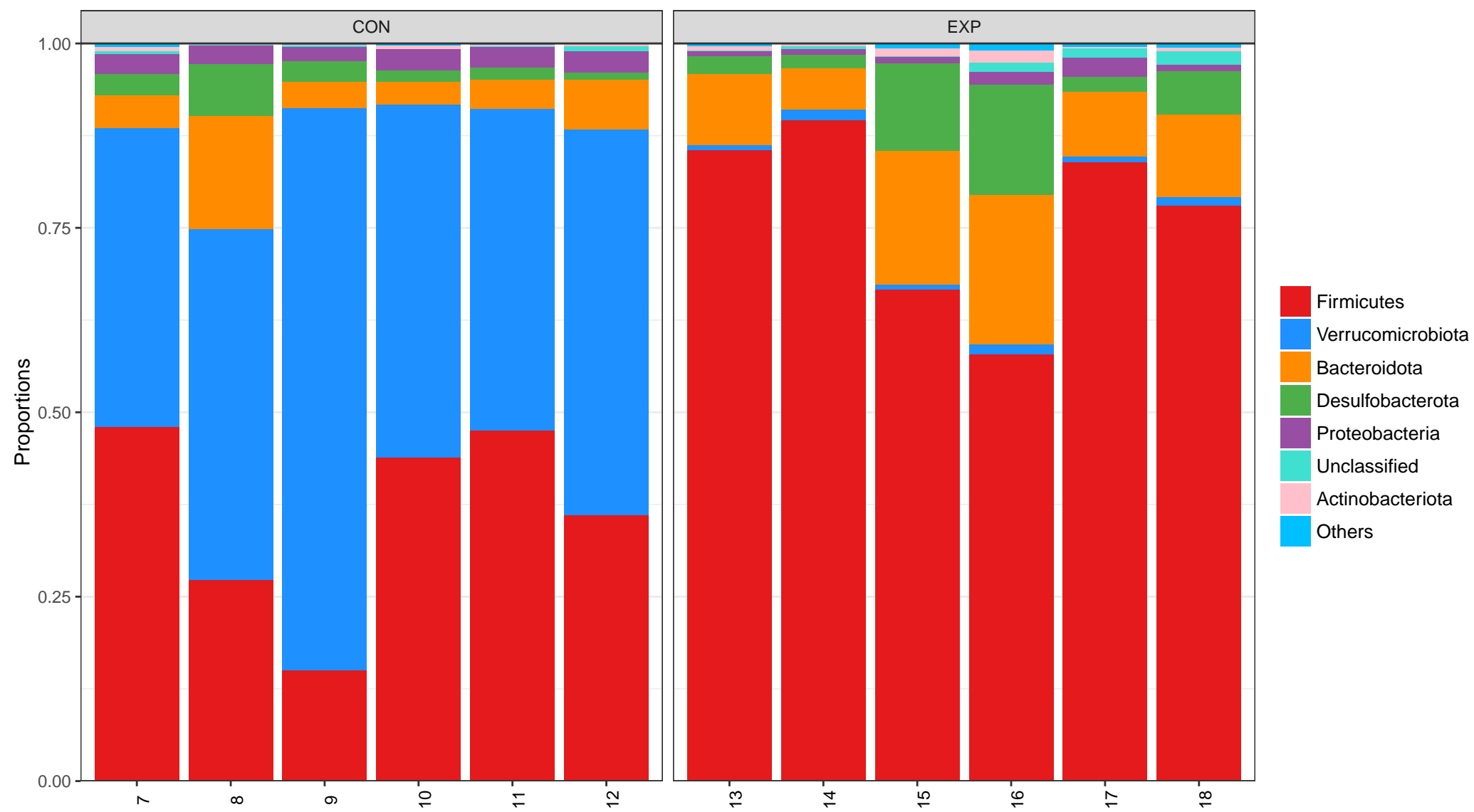

Supplement: Supplementary file 1 [file Data_Sheet_1.ZIP › Source data/gut microbiota diversity analysis/CON-EXP/03.Community_composition/02.Community/bar.ALL.phylum.xls.pdf]

## Similarity

### ***Taxonomic composition***

***Taxon***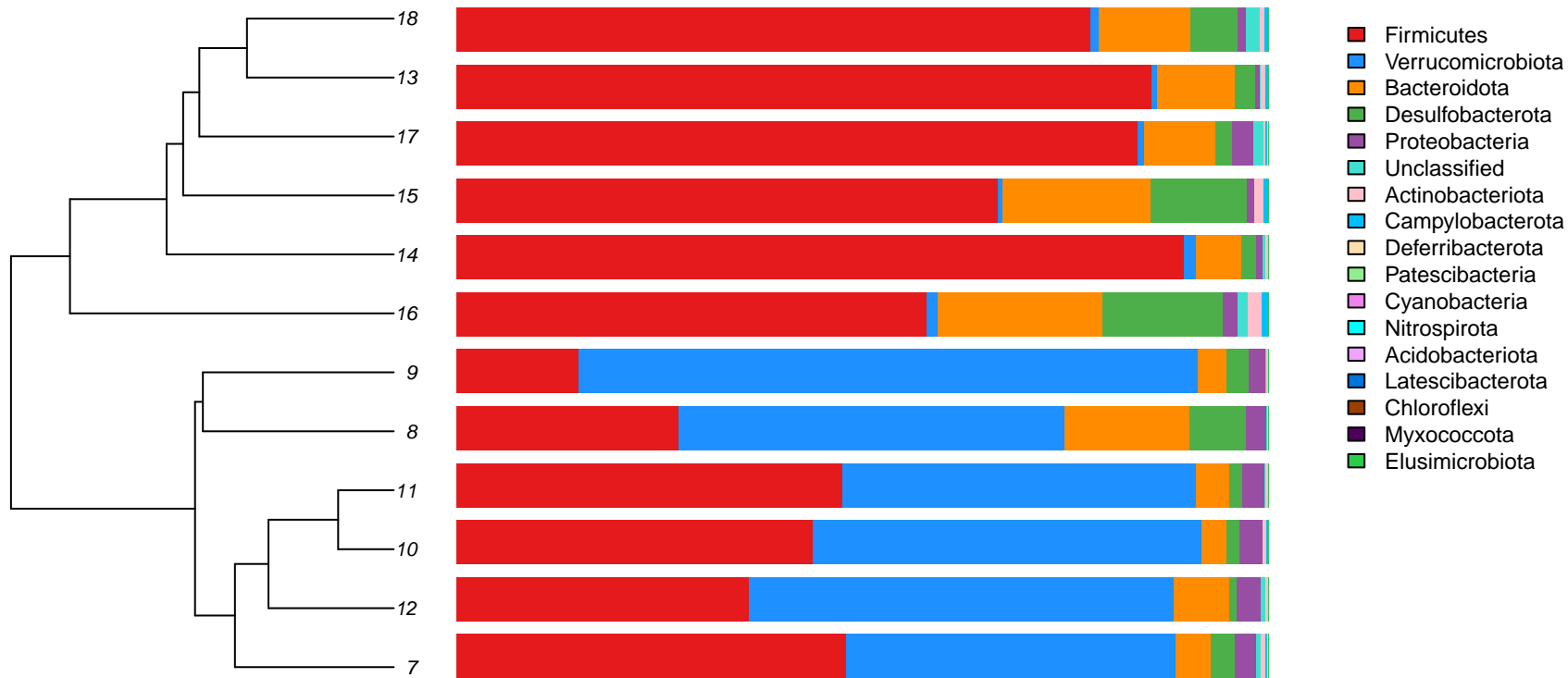

Supplement: Supplementary file 1 [file Data_Sheet_1.ZIP › Source data/gut microbiota diversity analysis/CON-EXP/03.Community_composition/03.Hcluster_bar/treebar_phylum.pdf]

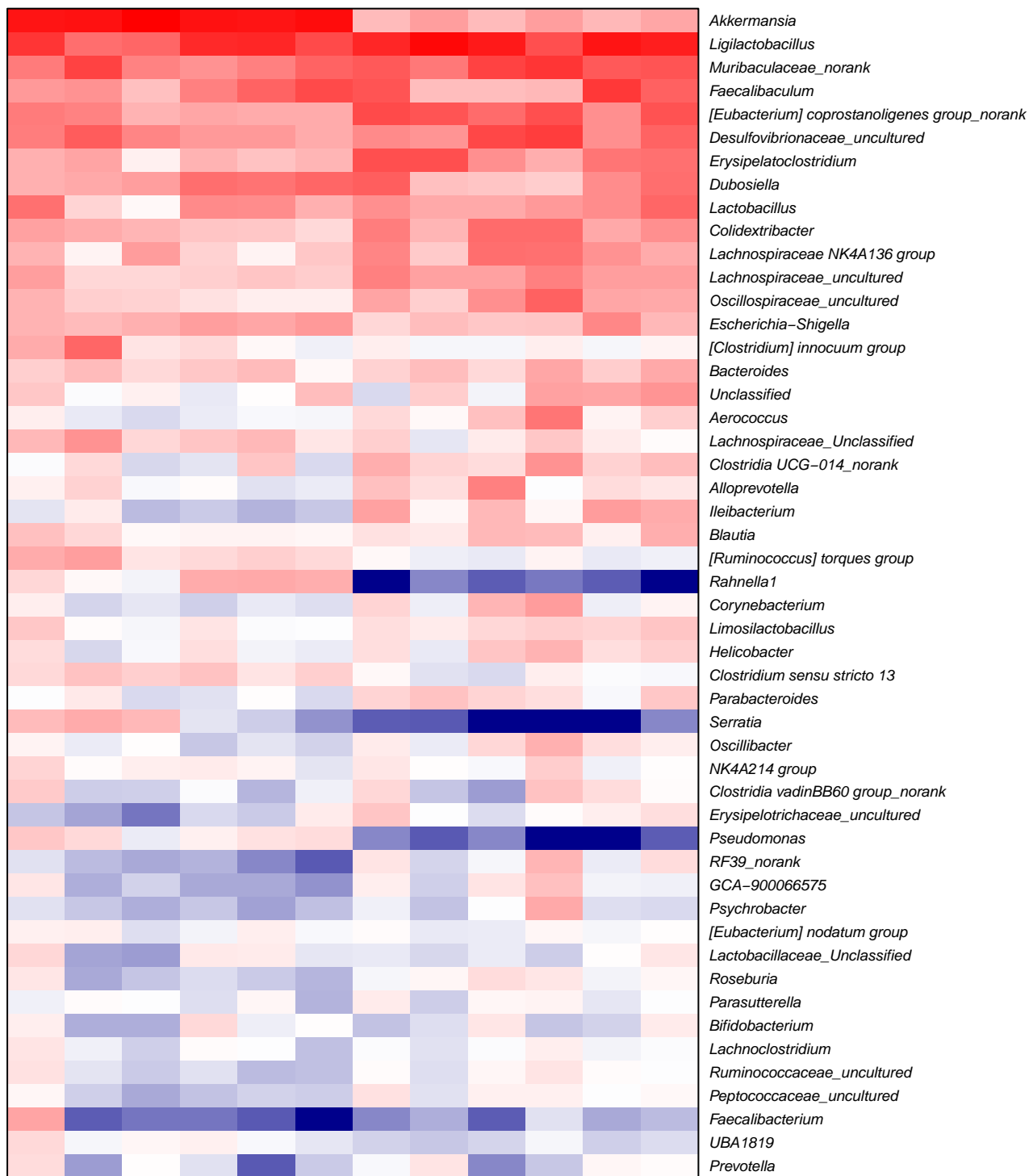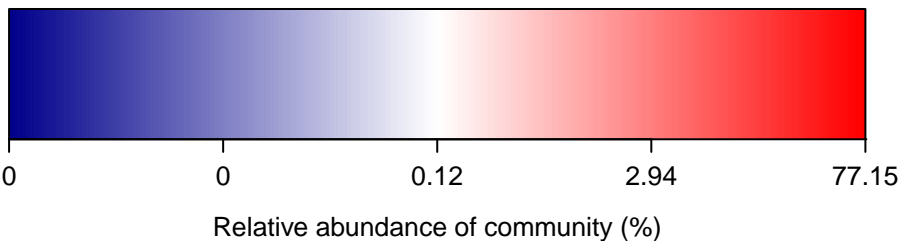

Supplement: Supplementary file 1 [file Data_Sheet_1.ZIP › Source data/gut microbiota diversity analysis/CON-EXP/03.Community_composition/04.Heatmap/heatmap_genus.pdf]

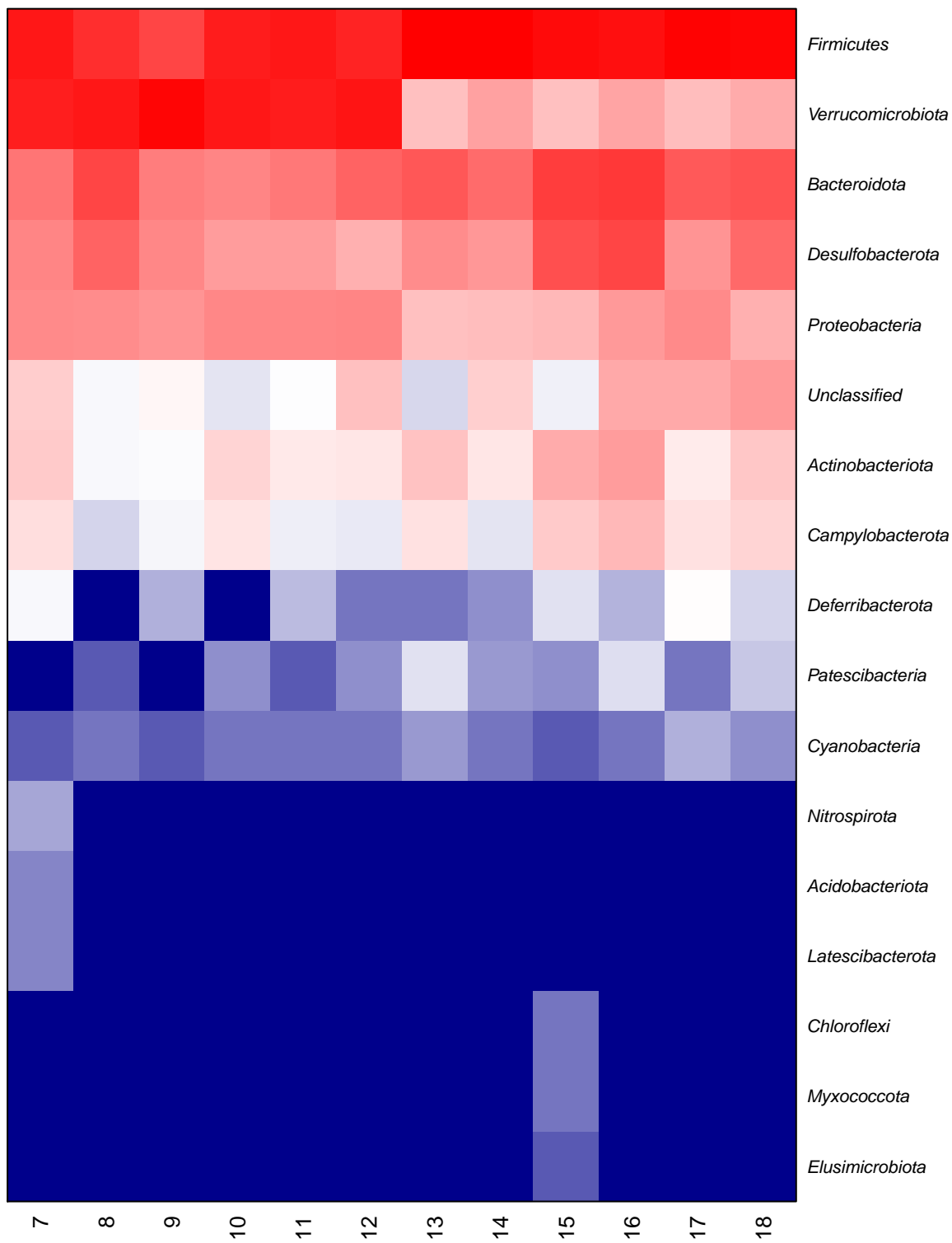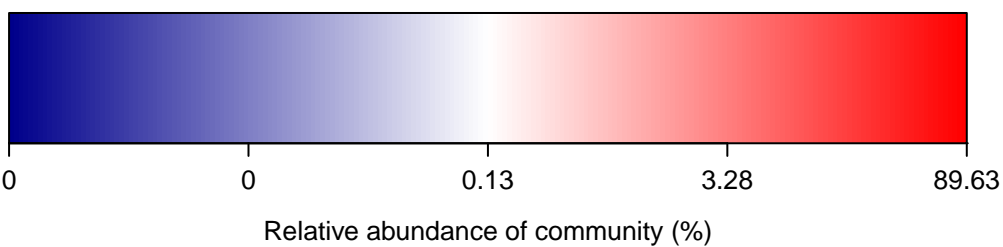

Supplement: Supplementary file 1 [file Data_Sheet_1.ZIP › Source data/gut microbiota diversity analysis/CON-EXP/03.Community_composition/04.Heatmap/heatmap_phylum.pdf]

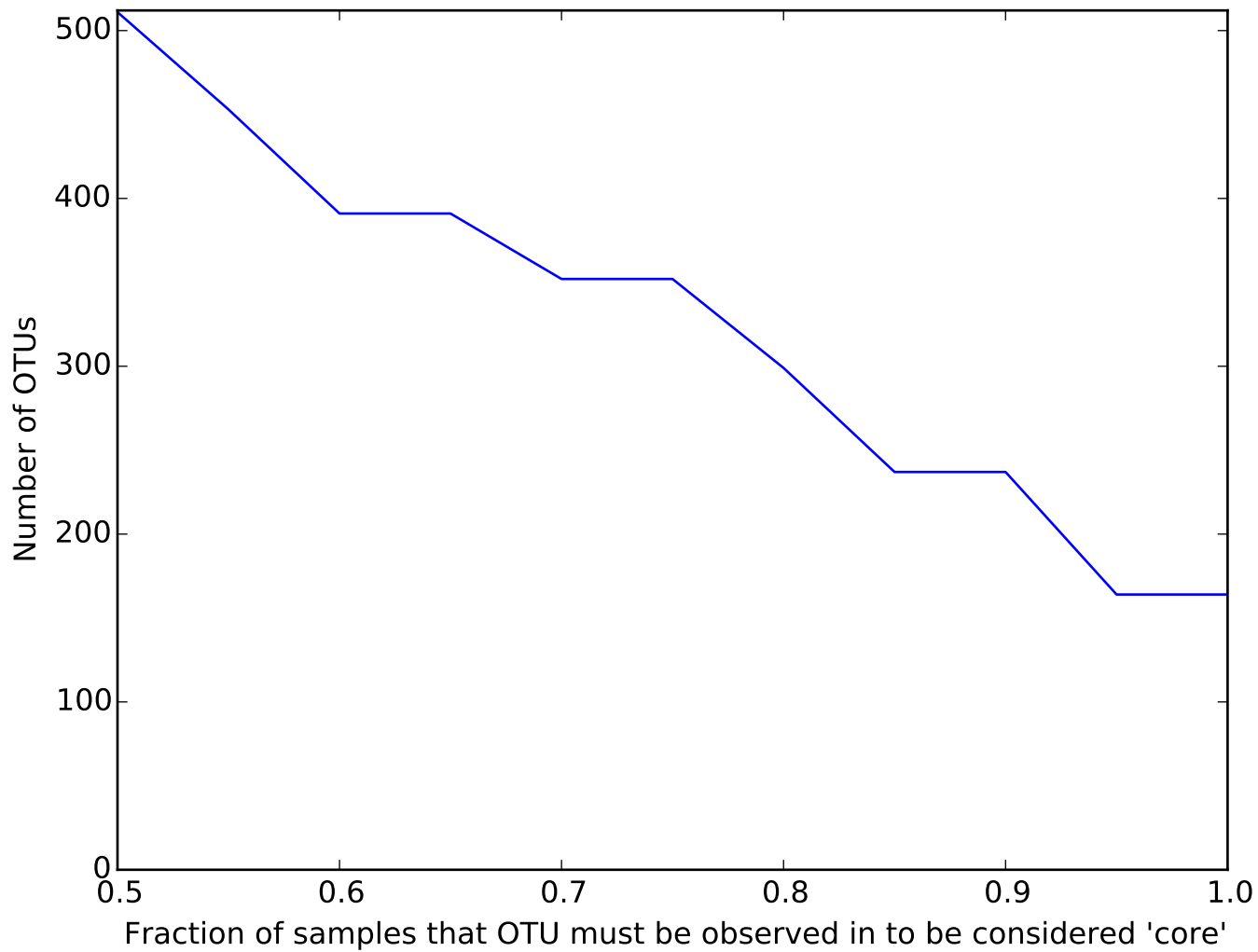

Supplement: Supplementary file 1 [file Data_Sheet_1.ZIP › Source data/gut microbiota diversity analysis/CON-EXP/03.Community_composition/05.Core/core_otu_size.pdf]

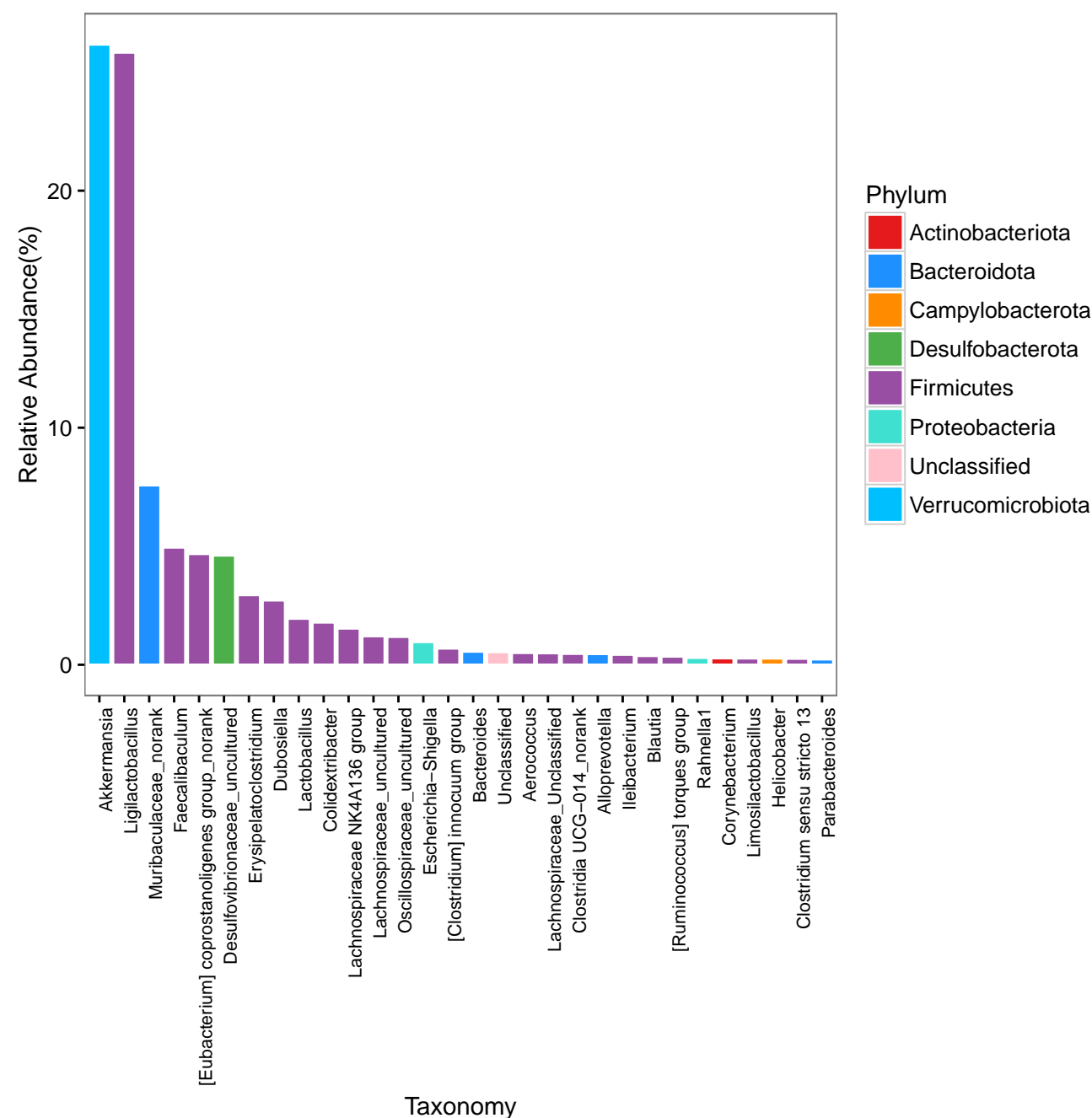

Supplement: Supplementary file 1 [file Data_Sheet_1.ZIP › Source data/gut microbiota diversity analysis/CON-EXP/03.Community_composition/06.Dominant_species/genus.taxonomy.top30.pdf]

# Group Distances

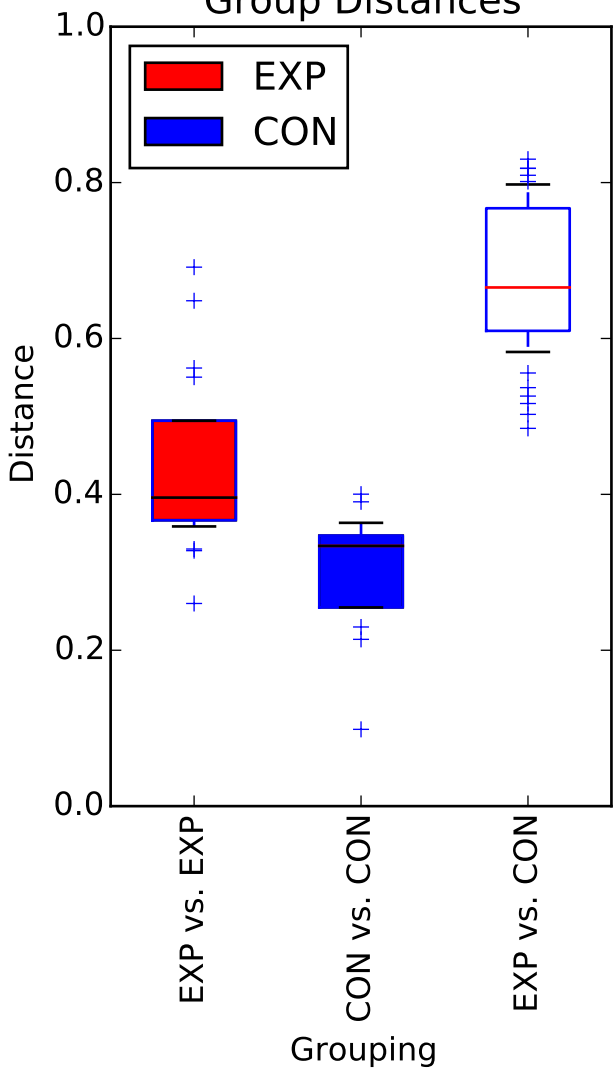

Supplement: Supplementary file 1 [file Data_Sheet_1.ZIP › Source data/gut microbiota diversity analysis/CON-EXP/04.Beta/02.Distance/bray_curtis/Group_Distances.pdf]

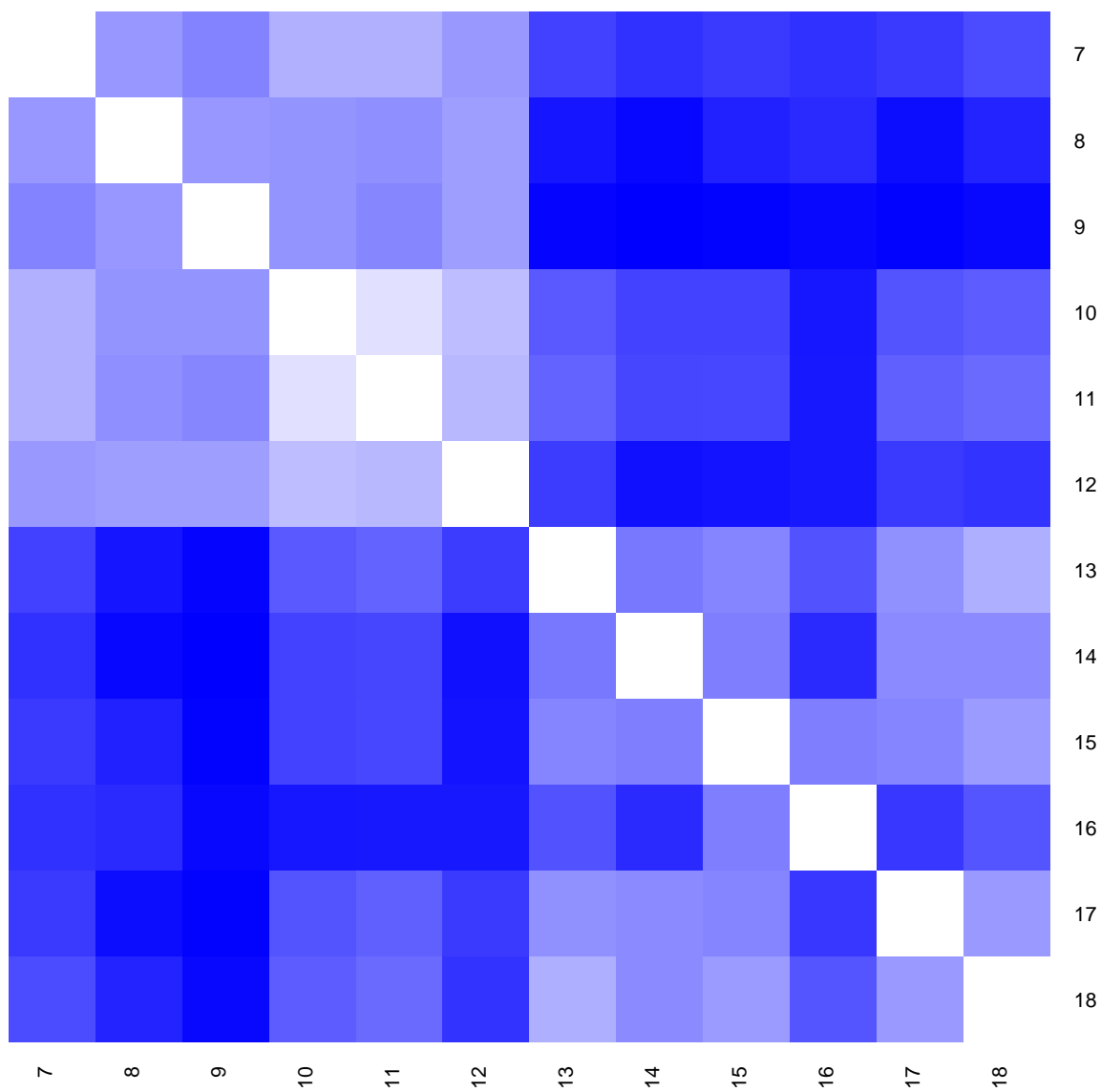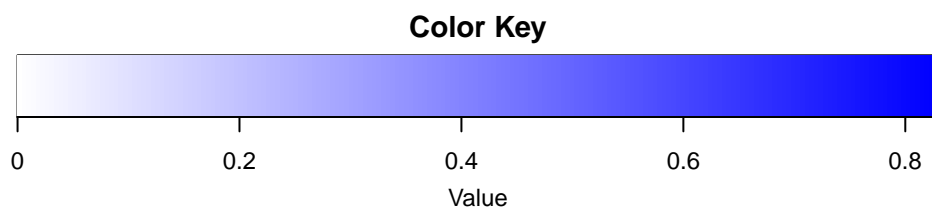

Supplement: Supplementary file 1 [file Data_Sheet_1.ZIP › Source data/gut microbiota diversity analysis/CON-EXP/04.Beta/02.Distance/bray_curtis/bray_curtis_otu_table.txt.all.pdf]

# Group Distances

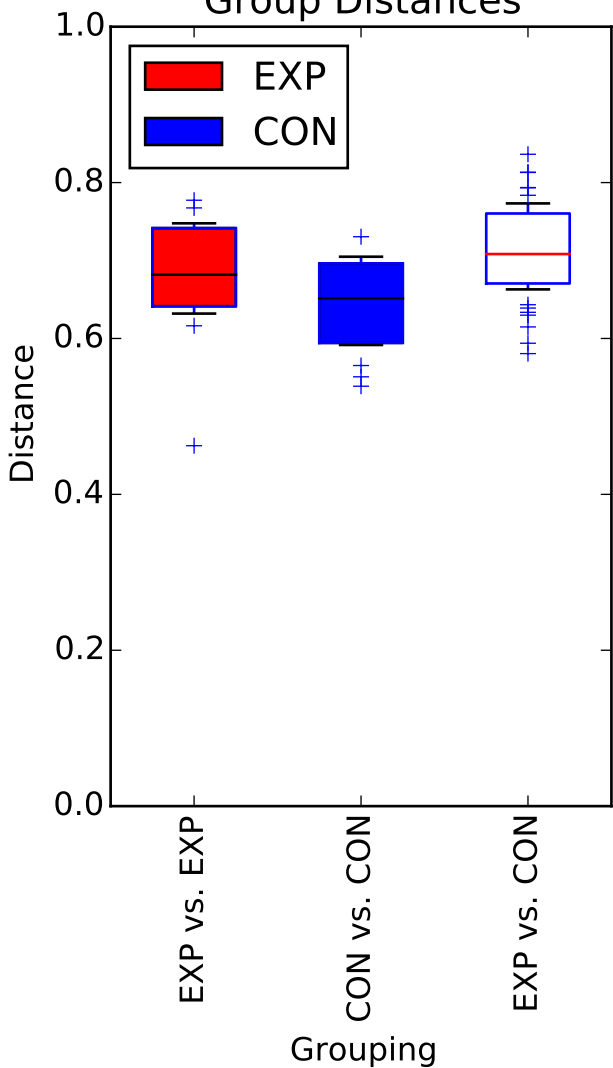

Supplement: Supplementary file 1 [file Data_Sheet_1.ZIP › Source data/gut microbiota diversity analysis/CON-EXP/04.Beta/02.Distance/unweighted_unifrac/Group_Distances.pdf]
