# Supplementary material for: β-sitosterol inhibits trimethylamine production by regulating the gut microbiota and attenuates atherosclerosis in ApoE–/– mice
Source: Front Cardiovasc Med. 2022 Nov 1;9:986905. doi: 10.3389/fcvm.2022.986905 (PMC9663806; doi:10.3389/fcvm.2022.986905)
Supplement: Supplementary file 1 [file Data_Sheet_1.ZIP › Source data/gut microbiota diversity analysis/CON-EXP/04.Beta/02.Distance/unweighted_unifrac/unweighted_unifrac_otu_table.txt.all.pdf]

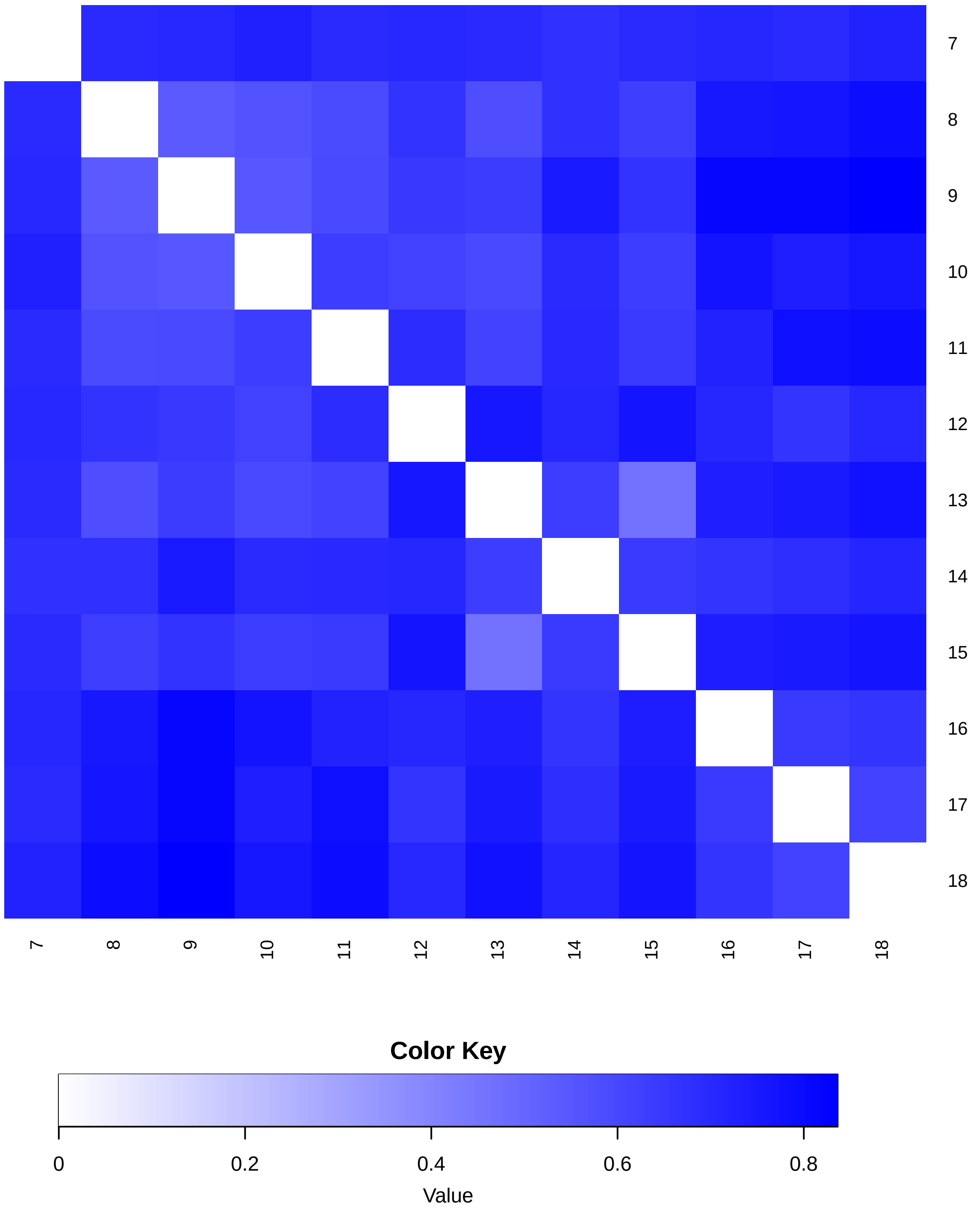
7
8
9
10
11
12
13
14
15
16
17
18
Color Key
0
0.2
0.4
0.6
0.8
Value
