# Supplementary material for: β-sitosterol inhibits trimethylamine production by regulating the gut microbiota and attenuates atherosclerosis in ApoE–/– mice
Source: Front Cardiovasc Med. 2022 Nov 1;9:986905. doi: 10.3389/fcvm.2022.986905 (PMC9663806; doi:10.3389/fcvm.2022.986905)
Supplement: Supplementary file 1 [file Data_Sheet_1.ZIP › Source data/gut microbiota diversity analysis/CON-EXP/04.Beta/02.Distance/weighted_unifrac/Group_Distances.pdf]

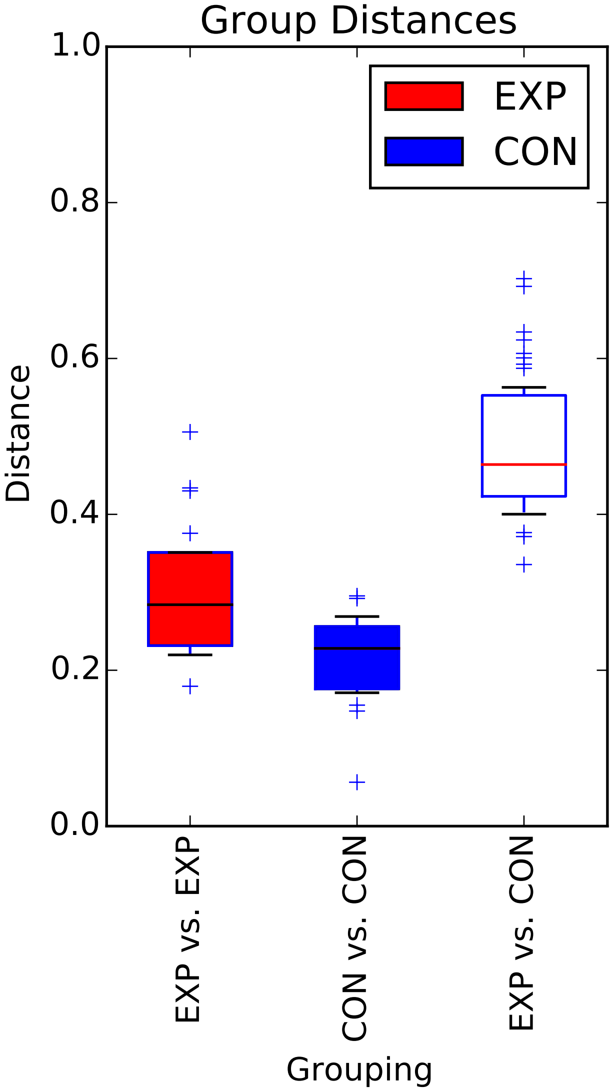

Group Distances
1.0
0.8
0.6
0.4
0.2
0.0
Distance
EXP
CON
EXP vs. EXP
CON vs. CON
EXP vs. CON
Grouping
