# Supplementary material for: β-sitosterol inhibits trimethylamine production by regulating the gut microbiota and attenuates atherosclerosis in ApoE–/– mice
Source: Front Cardiovasc Med. 2022 Nov 1;9:986905. doi: 10.3389/fcvm.2022.986905 (PMC9663806; doi:10.3389/fcvm.2022.986905)
Supplement: Supplementary file 1 [file Data_Sheet_1.ZIP › Source data/gut microbiota diversity analysis/CON-EXP/04.Beta/03.PCA/3d-pca_Group_pc1-2-3.pdf]

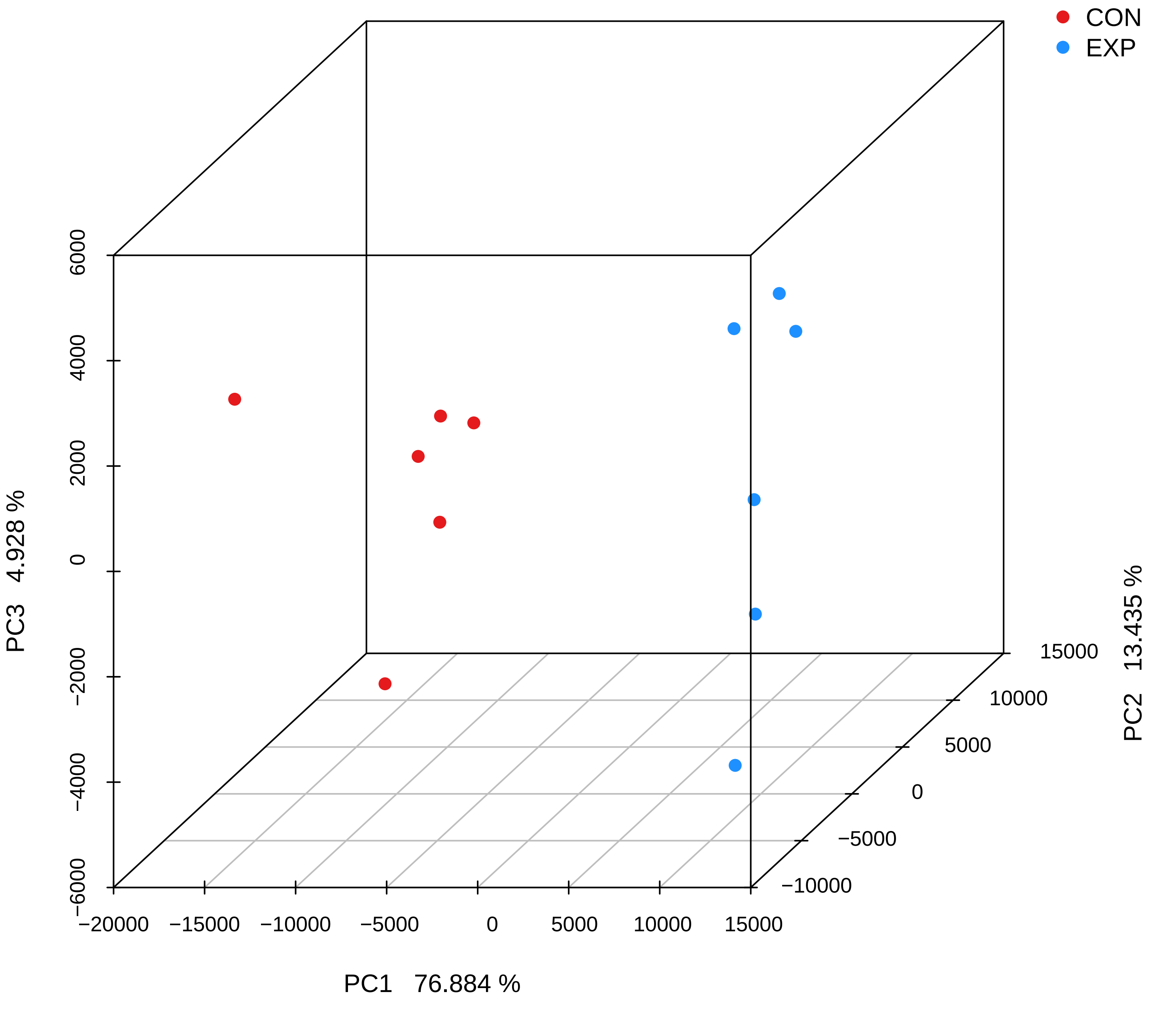
CON
EXP
PC1 76.884 %
PC2 13.435 %
PC3 4.928 %
−20000
−15000
−10000
−5000
0
5000
10000
15000
−10000
−5000
0
5000
10000
15000
−6000
−4000
−2000
0
2000
4000
6000
