# Supplementary material for: β-sitosterol inhibits trimethylamine production by regulating the gut microbiota and attenuates atherosclerosis in ApoE–/– mice
Source: Front Cardiovasc Med. 2022 Nov 1;9:986905. doi: 10.3389/fcvm.2022.986905 (PMC9663806; doi:10.3389/fcvm.2022.986905)
Supplement: Supplementary file 1 [file Data_Sheet_1.ZIP › Source data/gut microbiota diversity analysis/CON-EXP/04.Beta/03.PCA/pca_Group_pc1-3.pdf]

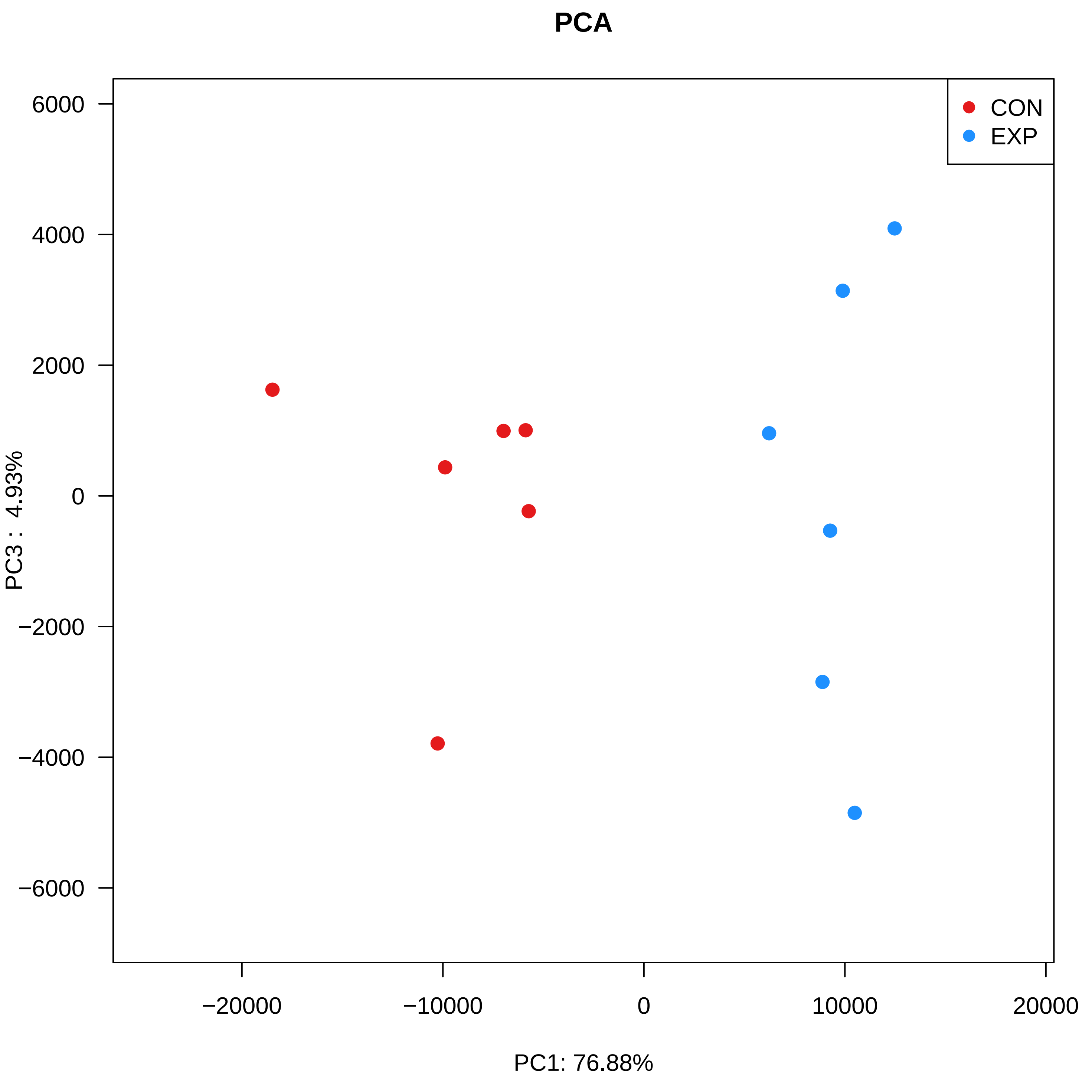

PCA
CON
EXP
6000
4000
2000
0
−2000
−4000
−6000
PC3 : 4.93%
−20000
−10000
0
10000
20000
PC1: 76.88%
