# Supplementary material for: β-sitosterol inhibits trimethylamine production by regulating the gut microbiota and attenuates atherosclerosis in ApoE–/– mice
Source: Front Cardiovasc Med. 2022 Nov 1;9:986905. doi: 10.3389/fcvm.2022.986905 (PMC9663806; doi:10.3389/fcvm.2022.986905)
Supplement: Supplementary file 1 [file Data_Sheet_1.ZIP › Source data/gut microbiota diversity analysis/CON-EXP/04.Beta/04.PCOA/bray_curtis/3d-pcoa_bray_curtis_Group_pc1-2-3.pdf]

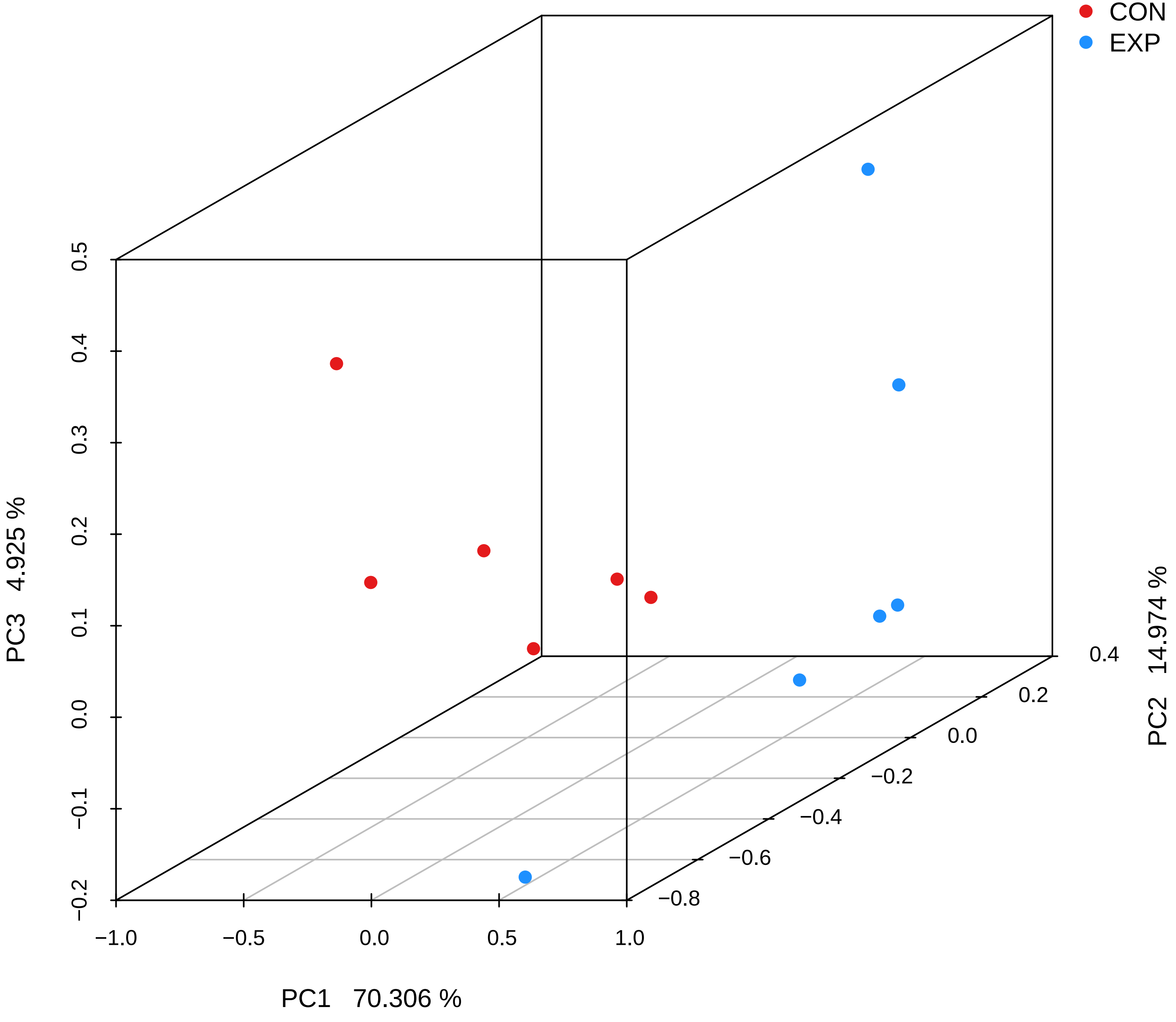
CON
EXP
PC1 70.306 %
PC2 14.974 %
PC3 4.925 %
−1.0
−0.5
0.0
0.5
1.0
−0.8
−0.6
−0.4
−0.2
0.0
0.2
0.4
−0.2
−0.1
0.0
0.1
0.2
0.3
0.4
0.5
