# Supplementary material for: β-sitosterol inhibits trimethylamine production by regulating the gut microbiota and attenuates atherosclerosis in ApoE–/– mice
Source: Front Cardiovasc Med. 2022 Nov 1;9:986905. doi: 10.3389/fcvm.2022.986905 (PMC9663806; doi:10.3389/fcvm.2022.986905)
Supplement: Supplementary file 1 [file Data_Sheet_1.ZIP › Source data/gut microbiota diversity analysis/CON-EXP/04.Beta/04.PCOA/bray_curtis/ellipse_pcoa_bray_curtis_otu_table.txt_pc1-2.pdf]

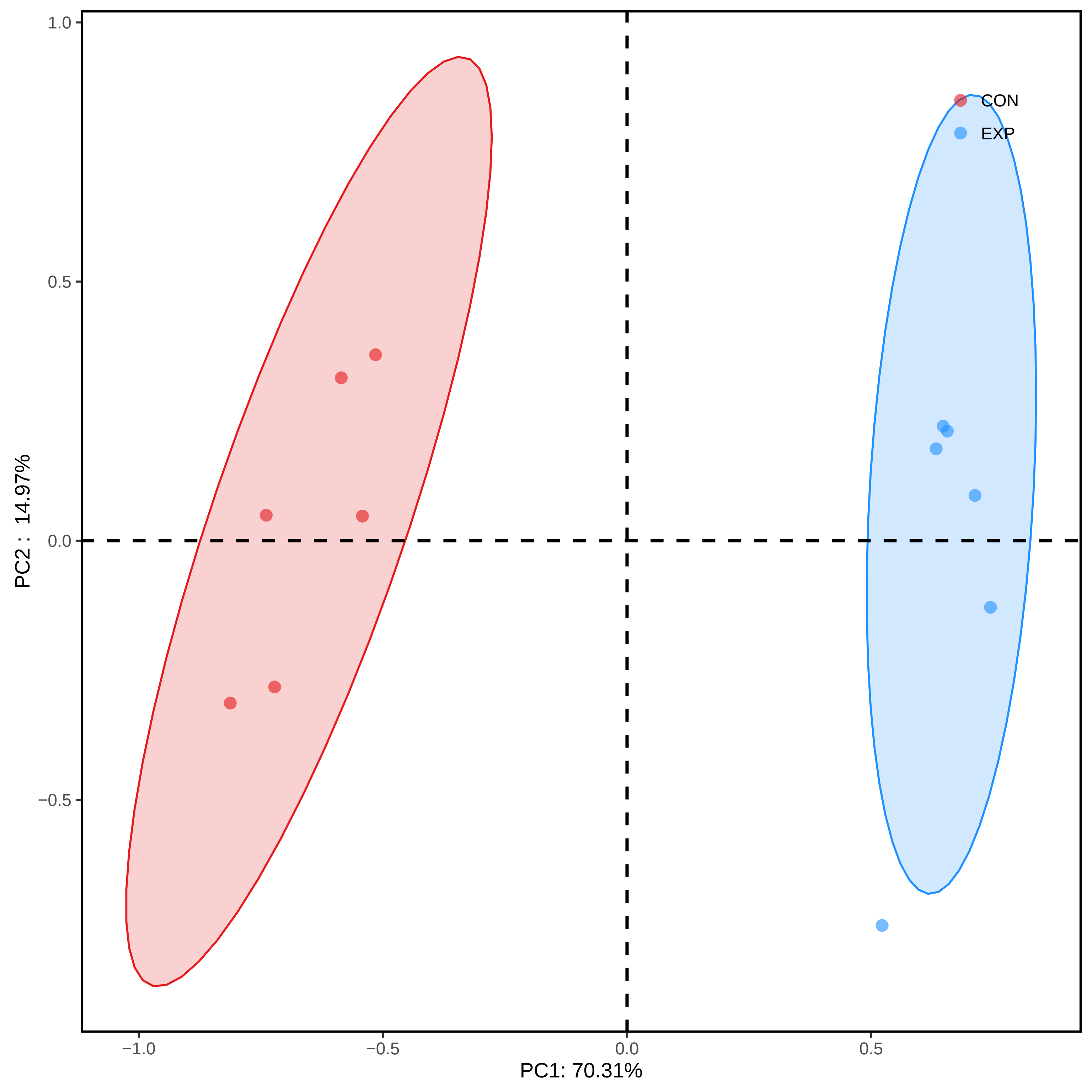
CON
EXP
1.0
0.5
0.0
−0.5
PC2 : 14.97%
−1.0
−0.5
0.0
0.5
PC1: 70.31%
