# Supplementary material for: β-sitosterol inhibits trimethylamine production by regulating the gut microbiota and attenuates atherosclerosis in ApoE–/– mice
Source: Front Cardiovasc Med. 2022 Nov 1;9:986905. doi: 10.3389/fcvm.2022.986905 (PMC9663806; doi:10.3389/fcvm.2022.986905)
Supplement: Supplementary file 1 [file Data_Sheet_1.ZIP › Source data/gut microbiota diversity analysis/CON-EXP/04.Beta/04.PCOA/unweighted_unifrac/3d-pcoa_unweighted_unifrac_Group_pc1-2-3.pdf]

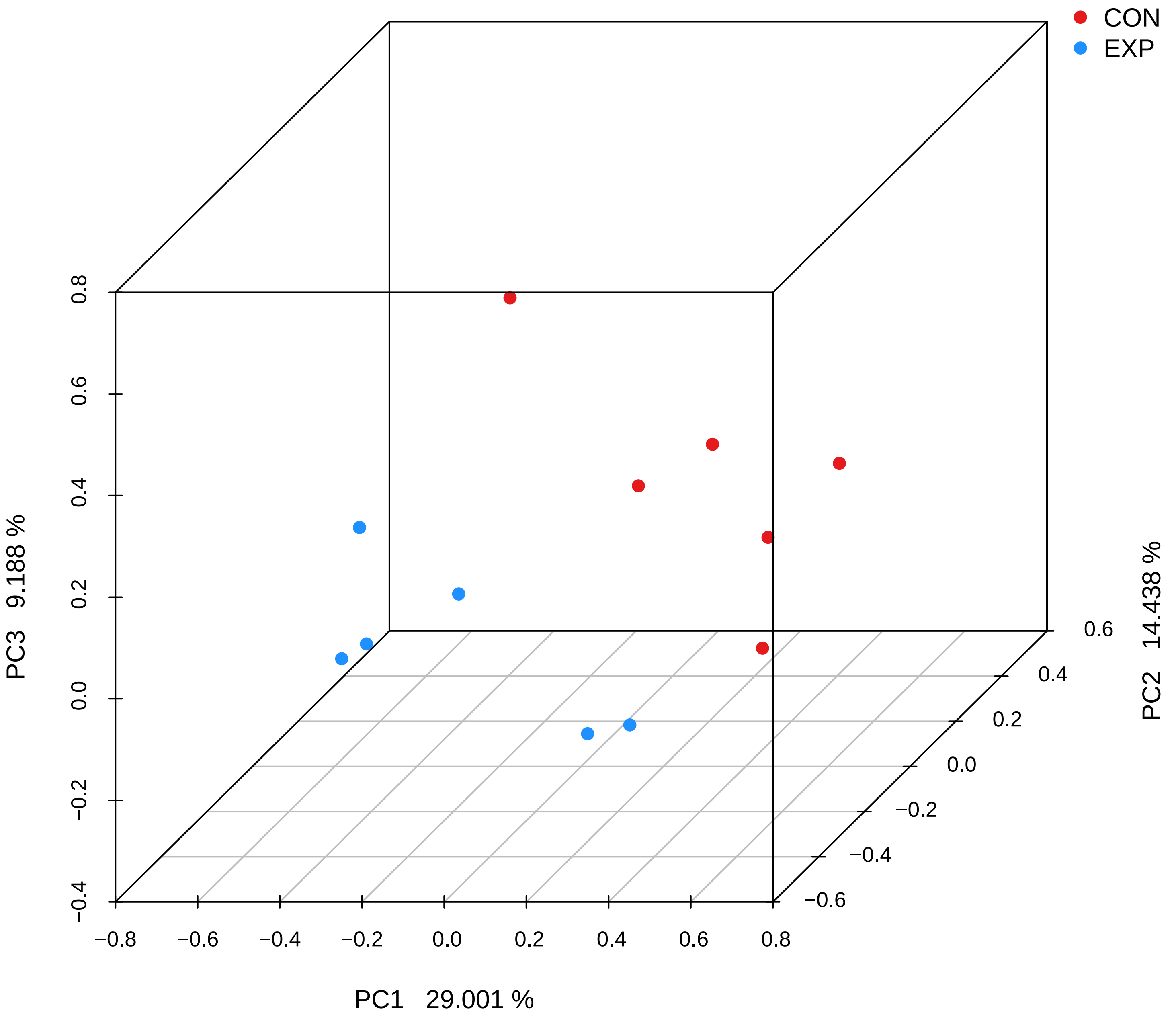

CON
EXP
PC1 29.001 %
PC2 14.438 %
PC3 9.188 %
−0.8
−0.6
−0.4
−0.2
0.0
0.2
0.4
0.6
0.8
