# Supplementary material for: β-sitosterol inhibits trimethylamine production by regulating the gut microbiota and attenuates atherosclerosis in ApoE–/– mice
Source: Front Cardiovasc Med. 2022 Nov 1;9:986905. doi: 10.3389/fcvm.2022.986905 (PMC9663806; doi:10.3389/fcvm.2022.986905)
Supplement: Supplementary file 1 [file Data_Sheet_1.ZIP › Source data/gut microbiota diversity analysis/CON-EXP/04.Beta/04.PCOA/unweighted_unifrac/pcoa_unweighted_unifrac_otu_table.txt_Group_pc1-3.pdf]

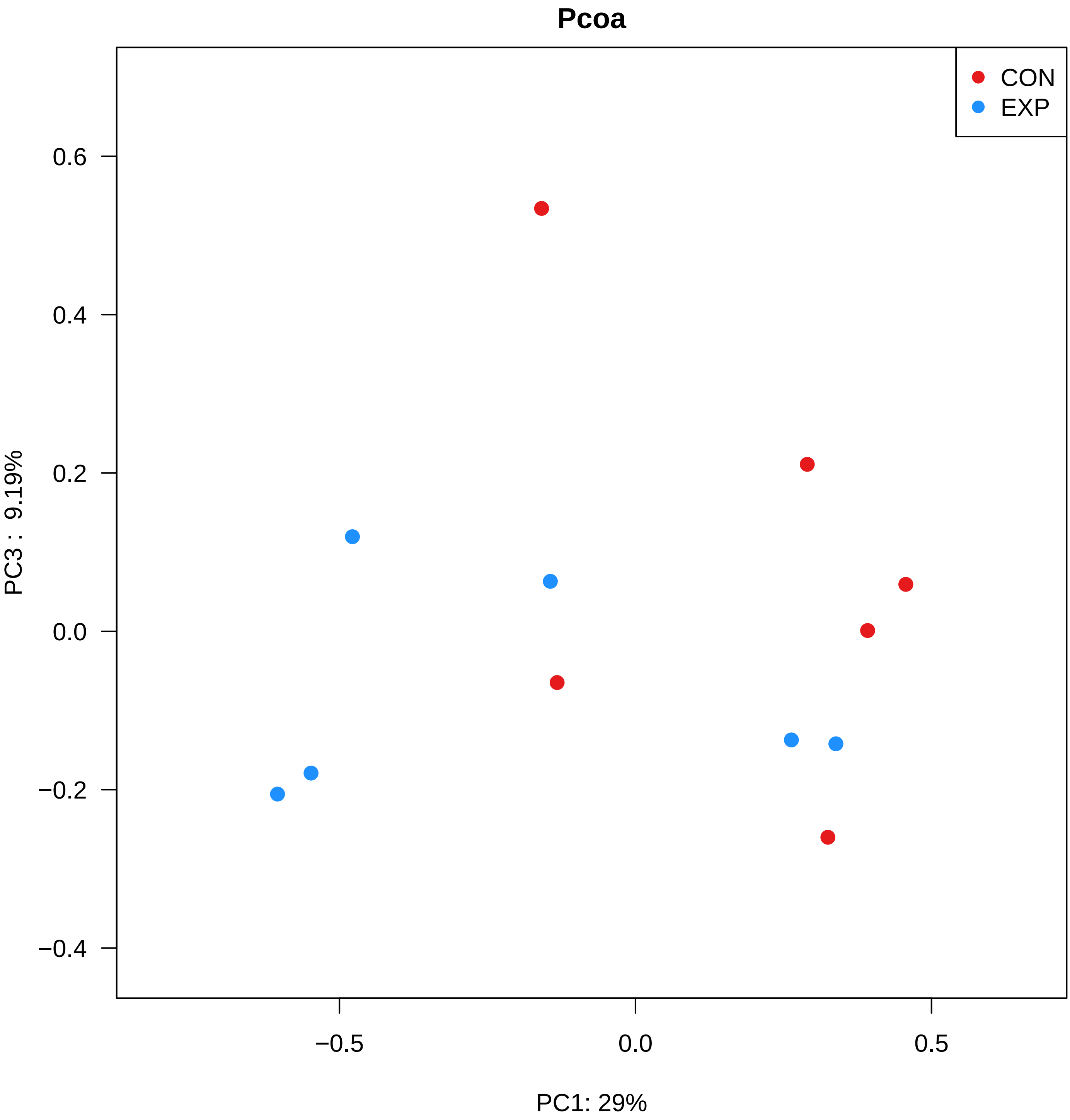
Pcoa
CON
EXP
0.6
0.4
0.2
0.0
−0.2
−0.4
PC3 : 9.19%
−0.5
0.0
0.5
PC1: 29%
