# Supplementary material for: β-sitosterol inhibits trimethylamine production by regulating the gut microbiota and attenuates atherosclerosis in ApoE–/– mice
Source: Front Cardiovasc Med. 2022 Nov 1;9:986905. doi: 10.3389/fcvm.2022.986905 (PMC9663806; doi:10.3389/fcvm.2022.986905)
Supplement: Supplementary file 1 [file Data_Sheet_1.ZIP › Source data/gut microbiota diversity analysis/CON-EXP/04.Beta/05.Nmds/unweighted_unifrac/ellipse_nmds_unweighted_unifrac_otu_table.txt.pdf]

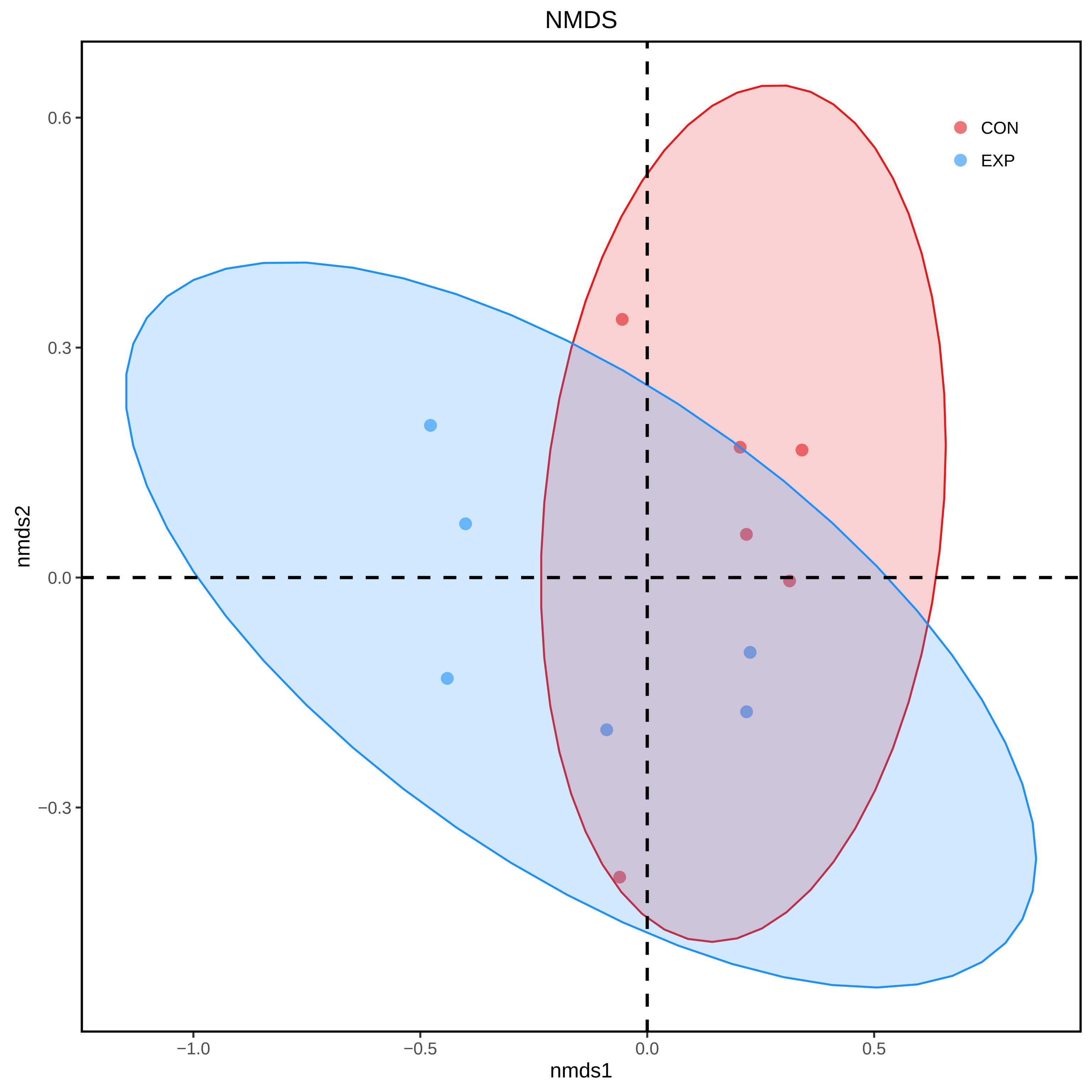

NMDS
CON
EXP
nmds2
nmds1
0.6
0.3
0.0
−0.3
−1.0
−0.5
0.0
0.5
