# Supplementary material for: β-sitosterol inhibits trimethylamine production by regulating the gut microbiota and attenuates atherosclerosis in ApoE–/– mice
Source: Front Cardiovasc Med. 2022 Nov 1;9:986905. doi: 10.3389/fcvm.2022.986905 (PMC9663806; doi:10.3389/fcvm.2022.986905)
Supplement: Supplementary file 1 [file Data_Sheet_1.ZIP › Source data/gut microbiota diversity analysis/CON-EXP/05.Differential_analysis/01.Lefse/lefse_LDA.cladogram.pdf]

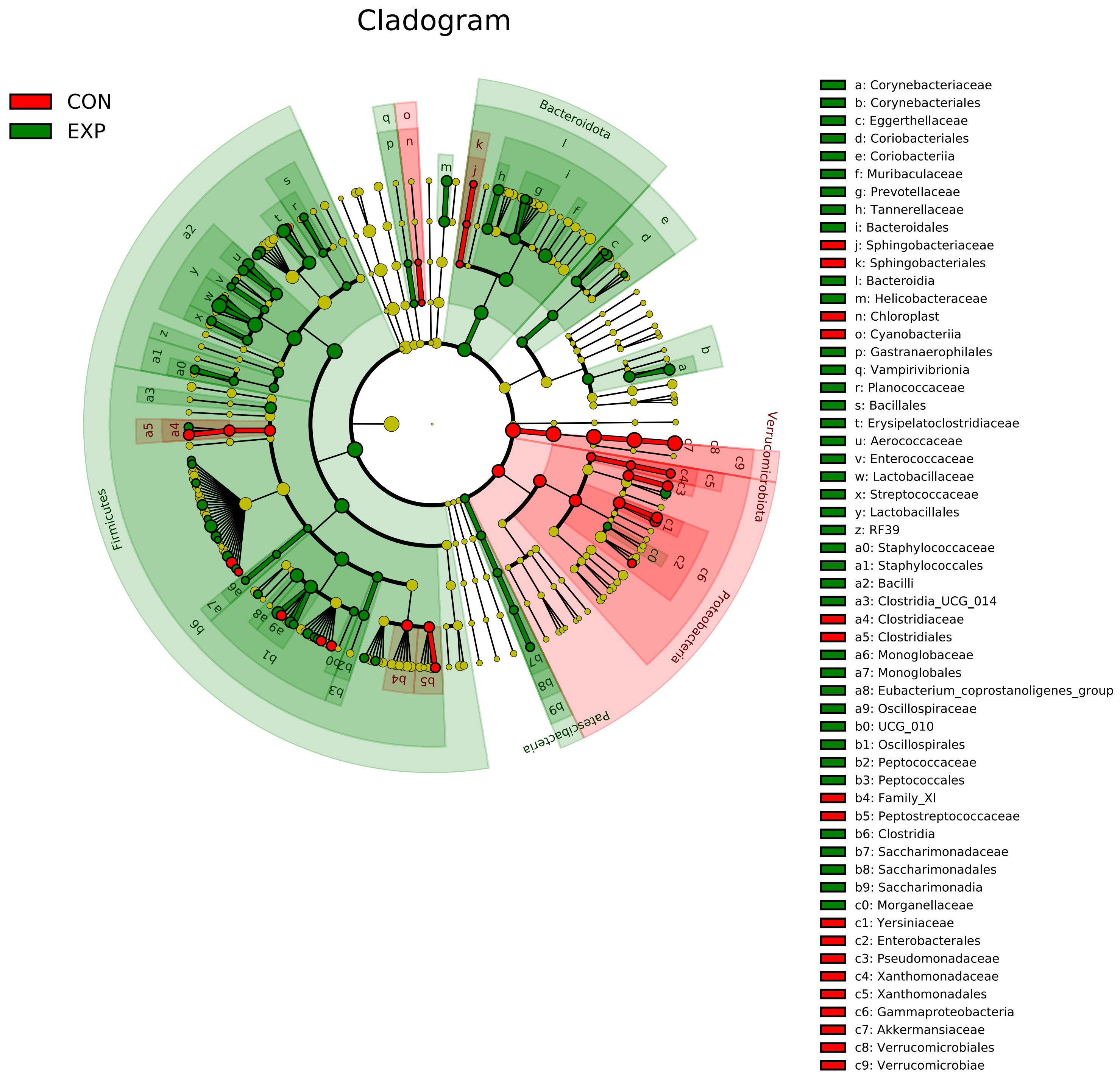
Cladogram
CON
EXP
Bacteroidota
Firmicutes
Verrucomicrobiota
Proteobacteria
Patescibacteria
a: Corynebacteriaceae
b: Corynebacteriales
c: Eggerthellaceae
d: Coriobacteriales
e: Coriobacteriia
f: Muribaculaceae
g: Prevotellaceae
h: Tannerellaceae
i: Bacteroidales
j: Sphingobacteriaceae
k: Sphingobacteriales
l: Bacteroidia
m: Helicobacteraceae
n: Chloroplast
o: Cyanobacteriia
p: Gastranaerophilales
q: Vampirivibrionia
r: Planococcaceae
s: Bacillales
t: Erysipelatoclostridiaceae
u: Aerococcaceae
v: Enterococcaceae
w: Lactobacillaceae
x: Streptococcaceae
y: Lactobacillales
z: RF39
a0: Staphylococcaceae
a1: Staphylococcales
a2: Bacilli
a3: Clostridia_UCG_014
a4: Clostridiaceae
a5: Clostridiales
a6: Monoglobaceae
a7: Monoglobales
a8: Eubacterium_coprostanoligenes_group
a9: Oscillospiraceae
b0: UCG_010
b1: Oscillospirales
b2: Peptococcaceae
b3: Peptococcales
b4: Family_XI
b5: Peptostreptococcaceae
b6: Clostridia
b7: Saccharimonadaceae
b8: Saccharimonadales
b9: Saccharimonadia
c0: Morganellaceae
c1: Yersiniaceae
c2: Enterobacterales
c3: Pseudomonadaceae
c4: Xanthomonadaceae
c5: Xanthomonadales
c6: Gammaproteobacteria
c7: Akkermansiaceae
c8: Verrucomicrobiales
c9: Verrucomicrobiae
