# Supplementary material for: β-sitosterol inhibits trimethylamine production by regulating the gut microbiota and attenuates atherosclerosis in ApoE–/– mice
Source: Front Cardiovasc Med. 2022 Nov 1;9:986905. doi: 10.3389/fcvm.2022.986905 (PMC9663806; doi:10.3389/fcvm.2022.986905)
Supplement: Supplementary file 1 [file Data_Sheet_1.ZIP › Source data/gut microbiota diversity analysis/CON-EXP/05.Differential_analysis/01.Lefse/lefse_LDA.pdf]

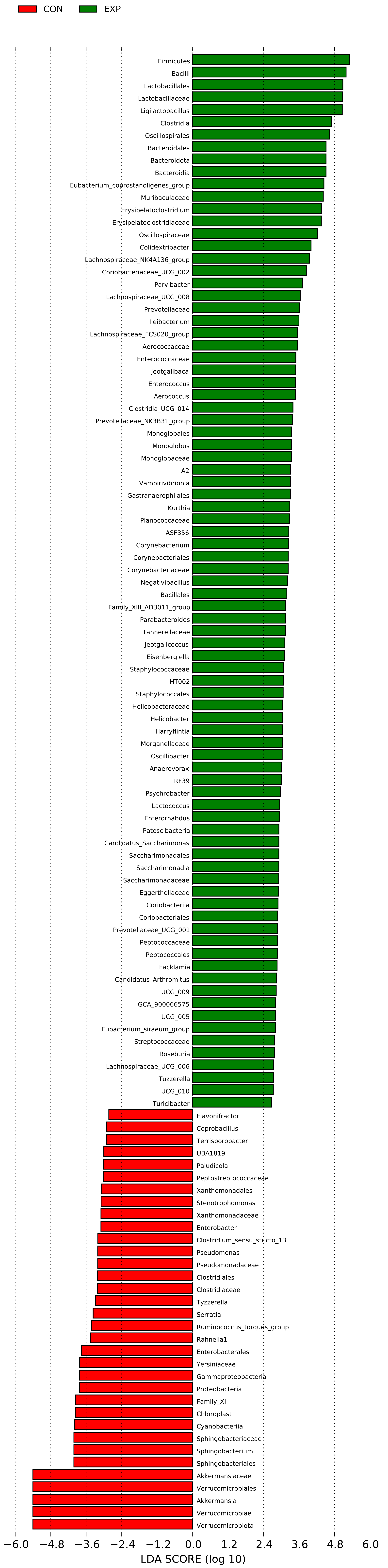

CON
EXP
Firmicutes
Bacilli
Lactobacillales
Lactobacillaceae
Ligilactobacillus
Clostridia
Oscillospirales
Bacteroidales
Bacteroidota
Bacteroidia
Eubacterium_coprostanoligenes_group
Muribaculaceae
Erysipelatoclostridium
Erysipelatoclostridiaceae
Oscillospiraceae
Colidextribacter
Lachnospiraceae_NK4A136_group
Coriobacteriaceae_UCG_002
Parvibacter
Lachnospiraceae_UCG_008
Prevotellaceae
Ileibacterium
Lachnospiraceae_FCS020_group
Aerococcaceae
Enterococcaceae
Jeotgalibaca
Enterococcus
Aerococcus
Clostridia_UCG_014
Prevotellaceae_NK3B31_group
Monoglobales
Monoglobus
Monoglobaceae
A2
Vampirivibrionia
Gastranaerophilales
Kurthia
Planococcaceae
ASF356
Corynebacterium
Corynebacteriales
Corynebacteriaceae
Negativibacillus
Bacillales
Family_XIII_AD3011_group
Parabacteroides
Tannerellaceae
Jeotgalicoccus
Eisenbergiella
Staphylococcaceae
HT002
Staphylococcales
Helicobacteraceae
Helicobacter
Harryflintia
Morganellaceae
Oscillibacter
Anaerovorax
RF39
Psychrobacter
Lactococcus
Enterorhabdus
Patescibacteria
Candidatus_Saccharimonas
Saccharimonadales
Saccharimonadia
Saccharimonadaceae
Eggerthellaceae
Coriobacteriia
Coriobacteriales
Prevotellaceae_UCG_001
Peptococcaceae
Peptococcales
Facklamia
Candidatus_Arthromitus
UCG_009
GCA_900066575
UCG_005
Eubacterium_siraeum_group
Streptococcaceae
Roseburia
Lachnospiraceae_UCG_006
Tuzzerella
UCG_010
Turicibacter
Flavonifractor
Coprobacillus
Terrisporobacter
UBA1819
Paludicola
Peptostreptococcaceae
Xanthomonadales
Stenotrophomonas
Xanthomonadaceae
Enterobacter
Clostridium_sensu_stricto_13
Pseudomonas
Pseudomonadaceae
Clostridiales
Clostridiaceae
Tyzzerella
Serratia
Ruminococcus_torques_group
Rahnella1
Enterobacterales
Yersiniaceae
Gammaproteobacteria
Proteobacteria
Family_XI
Chloroplast
Cyanobacteriia
Sphingobacteriaceae
Sphingobacterium
Sphingobacteriales
Akkermansiaceae
Verrucomicrobiales
Akkermansia
Verrucomicrobiae
Verrucomicrobiota
−6.0
−4.8
−3.6
−2.4
−1.2
0.0
1.2
2.4
3.6
4.8
6.0
LDA SCORE (log 10)
