# Supplementary material for: β-sitosterol inhibits trimethylamine production by regulating the gut microbiota and attenuates atherosclerosis in ApoE–/– mice
Source: Front Cardiovasc Med. 2022 Nov 1;9:986905. doi: 10.3389/fcvm.2022.986905 (PMC9663806; doi:10.3389/fcvm.2022.986905)
Supplement: Supplementary file 1 [file Data_Sheet_1.ZIP › Source data/gut microbiota diversity analysis/CON-EXP/report.html]

微生物群落多样性分析项目


## 微生物群落多样性分析项目

版权所有：上海凌恩生物科技有限公司  
联系电话：400-098 6026  
邮箱地址：ngs@biozeron.com

<

项目概况及整体流程介绍
:   一、 分析项目
:   二、 Illumina PE250测序实验流程
:   三、 生物信息分析流程

OTU聚类和物种注释
:   一、 原始数据样本区分与统计
:   二、 数据优化
:   三、 OTU聚类
:   四、 分类学分析
:   五、 分类学统计

Alpha多样性分析
:   一、 多样性指数
:   二、 稀释性曲线
:   三、 Shannon-Wiener曲线
:   四、 Rank-Abundance曲线
:   五、 Specaccum物种累积曲线

物种组成分析
:   一、 OTU分布Venn图
:   二、 群落结构组分图
:   三、 样本聚类树与柱状图组合分析
:   四、 群落Heatmap图
:   五、 Core
    microbiome分析
:   六
    、 优势物种群落结构组分图

Beta多样性比较分析
:   一、 组间距离分析
:   二、 PCA分析
:   三、 PCOA分析
:   四、 基于UniFrac的PCOA分析
:   五、 非度量多维尺度分析(NMDS)
:   六、 多样本相似度树状图
:   七、 基于UniFrac的聚类树分析
:   八、 ANOSIM相似性分析
:   九、 Adonis多因素方差分析

差异分析
:   一、 LEfSE分析
:   二、 ANOVA方差分析
:   三、 Wilcoxon秩和检验分析

附录
:   一、 文档结构
:   二、 文件打开或浏览方法
:   三、 分析方法说明（英文）
:   四、 参考文献

项目负责单位：上海凌恩生物科技有限公司

联系邮箱：project@biozeron.com

---

## 项目概况及整体流程介绍

### 一 、 分析项目

---

### 二 、 Illumina PE250测序实验流程

从DNA提取到上机测序，需要严谨、可靠的样本检测、质控流程，在每一环节都对样本质量严格控制，才能确保测序数据的真实可信。

### 2.1 基因组DNA抽提

完成基因组DNA抽提后，利用1%琼脂糖凝胶电泳检测抽提的基因组DNA。

### 2.2 PCR扩增

按指定测序区域，合成带有barcode的特异引物。

为保证后续数据分析的准确性及可靠性，需满足两个条件，1) 尽可能使用低循环数扩增；2)
保证每个样本扩增的循环数一致。随机选取具有代表性的样本进行预实验，确保在最低循环数中使绝大多数样本能够扩增出浓度合适的产物。

PCR 采用TransGen AP221-02：TransStart Fastpfu DNA Polymerase；

PCR仪：ABI GeneAmp® 9700型；

全部样本按照正式实验条件进行，每个样本3个重复，将同一样本的PCR产物混合后用2%琼脂糖凝胶电泳检测，使用AxyPrepDNA凝胶回收试剂盒（AXYGEN公司）切胶回收PCR产物，Tris\_HCl洗脱；
2%琼脂糖电泳检测。

### 2.3 荧光定量

参照电泳初步定量结果，将PCR产物用QuantiFluor™
-ST蓝色荧光定量系统（Promega公司）进行检测定量，之后按照每个样本的测序量要求，进行相应比例的混合。

### 2.4 Illumina PE250文库构建

1) 连接“Y”字形接头；

2) 使用磁珠筛选去除接头自连片段；

3) 利用PCR扩增进行文库模板的富集；

4) 氢氧化钠变性，产生单链DNA片段。

### 2.5 Illumina PE250测序

1) DNA片段的一端与引物碱基互补，固定在芯片上；

2) 另一端随机与附近的另外一个引物互补，也被固定住，形成“桥 (bridge)”；

3) PCR扩增，产生DNA簇；

4) DNA扩增子线性化成为单链。

5) 加入改造过的DNA聚合酶和带有4种荧光标记的dNTP，每次循环只合成一个碱基；

6) 用激光扫描反应板表面，读取每条模板序列第一轮反应所聚合上去的核苷酸种类；

7) 将“荧光基团”和“终止基团”化学切割，恢复3'端粘性，继续聚合第二个核苷酸；

8) 统计每轮收集到的荧光信号结果，获知模板DNA片段的序列。

### 三 、 生物信息分析流程

Illumina PE250测序得到的PE
reads首先根据overlap关系进行拼接，同时对序列质量进行质控和过滤，区分样本后进行OTU聚类分析和物种分类学分析，基于OTU聚类分析结果，可以对OTU进行多种多样性指数分析，以及对测序深度的检测；基于分类学信息，可以在各个分类水平上进行群落结构的统计分析。在上述分析的基础上，可以进行一系列群落结构和系统发育等深入的统计学和可视化分析。

## OTU聚类和物种注释

### 一 、 原始数据样本区分与统计

根据index序列区分各个样本的数据，提取出的数据以fastq格式保存，PE数据每个样本有fq1和fq2两个文件，里面为测序两端的reads，序列按顺序一一对应。

Fastq：Fastq是Solexa测序技术中一种反映测序序列的碱基质量的文件格式。每条read包含4 行信息，
其中第一行和的第三行由文件识别标志和读段名（ID）组成（第一行以“@”开头，第三行以“+” 开头；第 三行中 ID可以省略，但“+”不能省略），第二行为碱基序列，而第四行是第二行中的序列内容每个碱基所
对应的测序质量值。为方便保存和共享各实验室产生的高通量测序数据，NCBI数据中心建立了大容量的数 据库SRA（Sequence Read Archive，
http://www.ncbi.nlm.nih.gov/Traces/sra）来存放共享的原始测序数据。

@HWI-ST531R:144:D11RDACXX:4:1101:1212:1946 1:N:0:ATTCCT

ATNATGACTCAAGCGCTTCCTCAGTTTAATGAAGCTAACTTCAATGCTGAGATCGTTGACGACATCGAATGGG

+ HWI-ST531R:144:D11RDACXX:4:1101:1212:1946 1:N:0:ATTCCT

?A#AFFDFFHGFFHJJGIJJJIICHIIIIJJGGHIIJJIIJIIJIHGI@FEHIIJBFFHGJJIIHHHDFFFFDCCCCEDDCDDCDEACC

**FASTQ格式示例**

### 二 、 数据优化

### 2.1 数据优化与统计

Illumina PE250测序序列首先需要根据barcode得到所有样品的有效序列；然后对reads的质量进行质控过滤；接着根据PE
reads之间的overlap关系，将成对的reads拼接(merge)成一条序列；最后按照barcode和引物序列拆分得到每个样本的优质序列，并在过程中根据正反barcode和引物方向校正序列方向以及去除嵌合体。

#### 数据去杂方法和参数：

- 过滤read尾部质量值20以下的碱基，设置10bp的窗口，如果窗口内的平均质量值低于20，从窗口开始截去后端碱基，过滤质控后50bp以下的read；
- 根据PE
  reads之间的overlap关系，将成对reads拼接(merge)成一条序列，最小overlap长度为10bp；
- 拼接序列的overlap区允许的最大错配比率为0.2，筛除不符合序列；
- 根据序列首尾两端的barcode和引物区分样品，并调整序列方向，barcode允许的错配数为0，最大引物错配数为2；
- 用Usearch软件和gold数据库，采用denovo和reference结合的方式去除嵌合体

  

使用软件：Trimmomatic、FLASH、Usearch、qiime、自行开发的perl程序；

结果目录: 00.Data/

cleanData.stat.xls : 各样本优化序列统计表；

clean.data.len.pdf : 优化数据长度分布图；

clean.data.len.dist.txt : 优化数据长度分布；

#### 优化数据量统计及长度分布如下:

**Statistics of trimed sequences**

注：Trimed：分析时的优化数据；Samples：样本个数；Sequences：优化数据的序列条数；Bases：优化数据的碱基数；AverageLength：优化数据的序列平均长度。

  
**Length distribution of valid sequences**

注：Length：序列长度分布区间；Sequences：对应长度分布区间的序列条数；Percent：对应长度分布区间序列所占百分比。

### 2.2 各样本数据统计

各样本序列统计见下表：

**Statistics of trimed sequences**

注：Sample：样本名称；Sequences：样本分析时的优化数据的序列条数；Bases：样本优化序列碱基数；AverageLength：样本优化数据的序列平均长度。

### 三 、 OTU聚类

#### 分析方法

OTU（Operational Taxonomic
Units）是在系统发生学或群体遗传学研究中，为了便于进行分析，人为给某一个分类单元（品系，属，种、分组等）设置的同一标志。要了解一个样本测序结果中的菌种、菌属等数目信息，就需要对序列进行归类操作（cluster）。通过归类操作，将序列按照彼此的相似性分归为许多小组，一个小组就是一个OTU。可根据不同的相似度水平，对所有序列进行OTU划分，通常在97%的相似水平下的OTU进行生物信息统计分析。

软件平台：Usearch（vsesion 10 http://drive5.com/uparse/）

分析步骤如下：

- 对优化序列提取非重复序列，便于降低分析中间过程冗余计算量（http://drive5.com/usearch/manual/dereplication.html）；
- 去除没有重复的单序列（http://drive5.com/usearch/manual/singletons.html)；
- 按照97%相似性对非重复序列（不含单序列）进行OTU聚类，在聚类过程中去除嵌合体，得到OTU的代表序列。
- 将所有优化序列map至OTU代表序列，选出与OTU代表序列相似性在97%以上的序列，生成OTU表格。

#### 结果说明

结果目录： 01.OTU\_Taxa/

original/ : 原始的非抽平结果；

normalize/ : 抽平的结果；

otu\_table.xls: 各样本OTU中序列数统计表；

OTU聚类得到每个样本中各个OTU的丰度，结果形式为每行是一个OTU在不同样本中的序列数，每列对应一个样本。

  
**OTU table**

注：由于数据表较大，此处以图例形式列出部分数据，分析数据表格见附件中对应文件。

otu\_reps.fasta ： fasta格式OTU代表序列；

otu\_seqids.txt : 每个OTU中包含的序列编号列表。

otu\_table.biom : biom格式otu表。biom（Biological Observation Matrix）
格式是生物学样本中观察列联表的一种通用格式，具体信息参考http://biom-format.org/。

### 四 、 分类学分析

#### 分析方法

为了得到每个OTU对应的物种分类信息，采用uclust
算法对97%相似水平的OTU代表序列进行分类学分析，并分别在各个分类水平：domain（域），kingdom（界），phylum（门），class（纲），order（目），family（科），genus（属），species（种）统计各样本的群落组成。比对数据库如下:

16s细菌和古菌核糖体数据库（没有指定的情况下默认使用silva数据库）：

   &nbspSilva [4]（Release138.1 http://www.arb-silva.de）；

   &nbspRDP [5]（Release 11.5 http://rdp.cme.msu.edu/）；

   &nbspGreengene [6]（Release 13.8 http://greengenes.secondgenome.com/）;

ITS真菌：

   &nbspUnite[7]（Release 8.2 http://unite.ut.ee/index.php）的真菌数据库。

功能基因：

   &nbspFGR[8]，RDP整理来源于GeneBank的（Release7.3 http://fungene.cme.msu.edu/）的功能基因数据库。

软件及算法：

   &nbspQiime平台（V1.9.0http://qiime.org/scripts/assign\_taxonomy.html），
RDP Classifier [9]（version 2.2 http://sourceforge.net/projects/rdp-classifier/），置信度阈值为0.7。

#### 结果说明

结果目录： 01.OTU\_Taxa/

original/：原始的非抽平结果

normalize/：抽平的结果

otu\_taxa\_table.xls ： OTU分类学综合信息表，将OTU分析结果分类学信息结合得到的综合表；

**OTU and Taxonomy information of samples**

注：由于数据表较大，此处以图例形式列出部分数据，分析数据表格见附件中的对应文件。

OTU ID 为OTU编号；

第二列至taxonomy列的前一列为各样本的序列在各个OTU中的分布情况。

taxonomy列拉开可查看分类学系谱信息。各级分类水平以“；”隔开，分类学名称前的单个字母为分类等级的首字母缩写，以“\_\_”隔开。

tax\_summary\_a/ ： 各分类学水平样本序列数统计表

tax\_summary\_r/ ： 各分类学水平样本序列数相对丰度百分比统计表；

otu\_taxa\_table.biom : biom格式otu物种分类表。biom（Biological Observation
Matrix） 格式是生物学样本中观察列联表的一种通用格式，具体信息参考http://biom-format.org/。

  

物种分类中有一些特殊的名称，代表的意义如下：

(1) norank 分类学系谱中没有这个层级的科学名称，用norank做标记

(2) Uncultured 未分离培养的环境序列

(3) Unclassified 在置信度阈值下，与数据库比对不上

(4) \*\_norank(\*\_uncultured、\*\_unclassified)
在当前分类级别没有物种信息，用已知的最近的祖先分类名称+norank(uncultured、unclassified)表示。

(5) 结果说明 ：
original目录下存放的是直接聚类注释得到的丰度表，后续alpha多样性分析是基于这个非抽平的表做的normal目录下存放的抽平后的丰度表(按照所有样本中最低的数据量进行抽平。如果抽平的数据量高于部分样本的数据量，那么这些样本抽平后就会丢失)，后续物种组成分析、beta比较分析、差异分析和相关性分析等，都是基于抽平后的表做的。这主要是为了排除不同样本数据量之间差异影响样本本身的差异，使结果更加精确。

### 五、分类学统计

分别在各个分类水平：domain（域），phylum（门），class（纲），order（目），family（科），genus（属），species（种）统计注释到的物种比例。

#### 结果说明

结果目录： 01.OTU\_Taxa/02.normalize/

-tax\_summary\_a/\*layer.stat.xls：各分类学水平信息统计表

**总物种信息统计**

**门水平以下分类信息统计**

注意：norank，Uncultured，Unclassified未统计在内，其他水平统计信息见结果目录。

  

## 样本多样性分析（Alpha-diversity）

### 一 、 多样性指数

群落生态学中研究微生物多样性，通过单样本的多样性分析（Alpha多样性）可以反映微生物群落的丰度和多样性，包括一系列统计学分析指数估计环境群落的物种丰度和多样性。

计算物种丰度（Community richness）的指数有：

  Chao - the Chao1 estimator (http://www.mothur.org/wiki/Chao)；

  Richness：物种丰富度为群落中丰度大于0的物种数之和，值越大表明群落中物种种类越丰富。

  

计算物种丰富度（Community diversity）的指数有：

  Shannon - the Shannon index (http://www.mothur.org/wiki/Shannon)；

  Simpson - the Simpson index (http://www.mothur.org/wiki/Simpson)；

  ACE指数：是利用稀有物种估算物种多样性的指数，其值越高代表群落物种种类越丰富。

  Evenness：又Pielou均匀度（Pielou’s evenness），为群落实际的Shannon指数与具有相同物种丰富度的群落中能够获得的最大Shannon指数的比值。

  

测序深度指数有：

  Coverage - the Good’s coverage (http://www.mothur.org/wiki/Coverage) 。

  

各指数算法详解： https://mp.weixin.qq.com/s/M8SEMfcIjGLr1fmZGnf1oA

  

分析软件： mothur [10] ( version v.1.30.1 http://www.mothur.org/wiki/Schloss\_SOP#Alpha\_diversity)指数分析，用于指数评估的OTU相似水平97%
(0.97)。

#### 结果说明

结果目录： 01.Estimators/

Estimators.html：所有指数分析结果列表。

**Estimators table**

注：由于数据样本较多，此处以图例形式列出部分，分析数据表格见附件中的对应文件。

Sample ID：样本名称；Reads：被分入所有OTU中的总优化序列数；

OTU：本次实验中该样本优化序列划分得到的OTU数目；

Reads，Chao，Richness，Shannon，Simpson，ACE，Evenness，coverage：分别表示各个指数；

0.97：相似性水平为0.97。

  

组间多样性指数boxplot分析如下：

**组间Chao指数boxplot图**

**组间Richness指数boxplot图**

**组间Shannon指数boxplot图**

**组间Simpson指数boxplot图**

**组间ACE指数boxplot图**

**组间Evenness指数boxplot图**

**组间coverage指数boxplot图**

### 二 、 稀释性曲线（Rarefaction curve）

#### 分析方法

稀释性曲线[11]是从样本中随机抽取一定数量的个体，统计这些个体所代表的物种数目，并以个体数与物种数来构建曲线。它可以用来比较测序数据量不同的样本中物种的丰富度，也可以用来说明样本的测序数据量是否合理。采用对序列进行随机抽样的方法，以抽到的序列数与它们所能代表OTU的数目构建rarefaction
curve，当曲线趋向平坦时，说明测序数据量合理，更多的数据量只会产生少量新的OTU，反之则表明继续测序还可能产生较多新的OTU。因此，通过作稀释性曲线，可得出样本的测序深度情况。

软件：使用97%相似度的OTU，利用mothur做rarefaction分析，利用R语言工具制作曲线图。

#### 结果说明

结果目录： 02.Rarefaction/

otu.\*.rarefaction.xls: 每个样本在不同取样值的OTU数目表

**Rarefaction**

注：由于数据样本较多，此处以图例形式列出部分，分析数据表格见附件中的对应文件。

Numsampled：随机取样数 （预设为从1开始每增加100计算一次直到本次该样本取样数）；

Lci\hci ：统计学中的下限和上限值；

rarefaction.all.pdf: 各样本在97%相似水平下的曲线图

**Rarefaction curves**

注：横坐标：随机抽取的测序数据量；纵坐标：观测到的OTU数量。

### 三 、 Shannon-Wiener曲线

#### 分析方法

Shannon-Wiener
[12]是反映样本中微生物多样性的指数，利用各样本的测序量在不同测序深度时的微生物多样性指数构建曲线，以此反映各样本在不同测序数量时的微生物多样性。当曲线趋向平坦时，说明测序数据量足够大，可以反映样本中绝大多数的微生物信息。

软件：使用97%相似度的OTU，利用mothur计算不同随机抽样下的shannon值，利用R语言工具制作曲线图。

#### 结果说明

结果目录： 03.Shannon\_rarefac/

otu.\*r.shannon.xls：每个样本在不同取样值的shannon指数表

**Shannon Wiener**

注：由于数据样本较多，此处以图例形式列出部分，分析数据表格见附件中的对应文件。

r\_shannon.All.pdf： shannon曲线图

**Shannon Wiener curves of samples**

### 四 、 Rank-Abundance曲线

#### 分析方法

Rank-abundance
[13]曲线是分析多样性的一种方式。构建方法是统计单一样本中，每一个OTU所含的序列数，将OTUs按丰度（所含有的序列条数）由大到小等级排序，再以OTU等级为横坐标，以每个OTU中所含的序列数（也可用OTU中序列数的相对百分含量）为纵坐标做图。

Rank-abundance曲线可用来解释多样性的两个方面，即物种丰度和物种均匀度。在水平方向，物种的丰度由曲线的宽度来反映，物种的丰度越高，曲线在横轴上的范围越大；曲线的形状（平滑程度）反映了样本中物种的均度，曲线越平缓，物种分布越均匀。

软件：利用R语言工具制作曲线图。

#### 结果说明

结果目录： 04.Rank\_Abundance/

rankabundance.pdf：多样本曲线图

注：横坐标：OTU等级，“500”代表样本中按照丰度排列第500位的OTU；纵坐标：该等级OTU中序列数的相对百分含量，即属于该OTU的序列数除以总序列数，纵坐标轴上数字，例如“100”代表相对丰度为100%，“10”代表相对丰度为10%，依次类推。

### 五 、 Specaccum物种累积曲线

#### 分析方法

物种累积曲线( species accumulation curves)[14]
是用于描述随着样本量的加大物种增加的状况，是调查样本的物种组成和预测样本中物种丰度的有效工具，在生物多样性和群落调查中，被广泛用于样本量是否充分的判断以及物种丰富度( species richness)
的估计。因此，通过物种累积曲线不仅可以判断样本量是否充分，在样本量充分的前提下，运用物种累积曲线还可以对物种丰富度进行预测（默认在样本量大于10个时分析）。

#### 结果说明

结果目录： 05.Specaccum/

specaccum.all.pdf：物种累计曲线图

**Species accumulation curves**

注：横坐标：样本量；纵坐标：抽样后OTU数目。结果反映了持续抽样下新OTU（新物种）出现的速率。在一定范围内，随着样本量的加大，若曲线表现为急剧上升则表示群落中有大量物种被发现；当曲线趋于平缓，则表示此环境中的物种并不会随样本量的增加而显著增多。利用物种累积曲线可以作为对样本量是否充分的判断，曲线急剧上升表明样本量不足，需要增加抽样量；反之，则表明抽样充分，可以进行数据分析。

## 物种组成分析

### 一 、 OTU分布Venn图

#### 分析方法

Venn图[15]可用于统计多个样本中所共有和独有的OTU数目，可以比较直观的表现环境样本的OTU数目组成相似性及重叠情况。通常情况下，分析时选用相似水平为97%的OTU样本表。

软件：R语言工具统计和作图。

#### 结果说明

结果目录： 01.Venn/

venn.pdf：文氏图（样本数为2~5）/花瓣图（样本数>=6）；

venn.sets.xls：文氏图中各部分的OTU名称。

**OTU Venn analysis**

注：不同的颜色代表不同的样本，如果两个不同颜色圆圈重叠的区域标注有数字100，说明这两个样本均有序列被划分入相同的OTU中，且这样的OTU有100个。

### 二 、 群落结构组分图

#### 分析方法

根据分类学分析结果，可以得知一个或多个样本在各分类水平上的分类学比对情况。在结果中，包含了两个信息：

- 样本中含有何种微生物；
- 样本中各微生物的序列数，即各微生物的相对丰度。

因此，可以使用统计学的分析方法，观测样本在不同分类水平上的群落结构。将多个样本的群落结构分析放在一起对比时，还可以观测其变化情况。根据研究对象是单个或多个样本，结果可能会以不同方式展示。通常使用较直观的饼图或柱状图等形式呈现[19]。群落结构的分析可在任一分类水平进行。

软件：基于tax\_summary\_a文件夹中的数据表，利用R语言工具作图或在EXCLE中编辑作图。

#### 结果说明

结果目录： 02.Community /

bar.All.\*.xls.pdf：多样本柱状图

 **Microbial community barplot**

注：为使视图效果最佳，作图时可将丰度低于1%的部分合并为other在图中显示。

### 三 、 样本聚类树与柱状图组合分析

#### 分析方法

左边是样本间基于群落组成的层次聚类分析（bray-curtis算法），右边是样本的群落结构柱状图[20]

#### 结果说明

结果目录： 03.Hcluster\_bar /

treebar\_phylum.pdf：样本聚类树与柱状图组合分析图

**Microbial community barplot with cluster tree**

### 四 、 群落Heatmap图

#### 分析方法

Heatmap[21]可以用颜色变化来反映二维矩阵或表格中的数据信息，它可以直观地将数据值的大小以定义的颜色深浅表示出来。常根据需要将数据进行物种或样本间丰度相似性聚类，将聚类后数据表示在heatmap图上，可将高丰度和低丰度的物种分块聚集，通过颜色梯度及相似程度来反映多个样本在各分类水平上群落组成的相似性和差异性。结果可有彩虹色和黑红色两种选择。

软件及算法：R语言vegan包，vegdist和hclust进行距离计算和聚类分析；距离算法：Bray-Curtis，聚类方法：complete。图中颜色梯度可自定为两种或两种以上颜色渐变色。样本间和物种间聚类树枝可自定是否画出。

#### 结果说明

结果目录： 04.Heatmap/

heatmap\_\*.pdf：heatmap图

**Microbial community heatmap analysis**

### 五 、 Core microbiome分析（总样品数>=5）

#### 分析方法

根据OTU在各样本中的丰度，找到在50%以上样本、60%以上样本、70%以上样本、80%以上样本、90%以上样本、所有样本中存在的core microbiome，并绘制core
microbiome曲线

#### 结果说明

结果目录： 05.Core/

core\_table\*.xls： table格式的core microbiome丰度表

Core\_table\*.biom： biom格式的core microbiome丰度表

**Core OTU and Taxonomy information of samples**

第一列OTU ID是OTU名称；

第二列...N-1列为样品OTU数量Taxonomy Level是OTU所代表的物种所在的分类水平；

第N列Taxonomy Name是OTU所代表的物种名称。

core\_otu\_size.pdf： core microbiome曲线

**core microbiome cruve**

### 六 、 优势物种群落结构组分图

#### 分析方法

选取单个或者所有样品丰度最高的一些物种（属水平）绘制柱状图并显示该物种对应的门水平分类信息，同一颜色物种表示来源于同一门类

#### 结果说明

结果目录： 06.Dominant\_species/

\*.taxonomy.top\*.pdf：优势物种柱状图

\*.taxonomy.top\*.xls：优势物种列表

**Dominant species barplot**

## 组间(样本间)比较分析(β多样性分析)

### 一 、 组间距离分析

样本间的物种或功能的丰度分布差异程度可通过统计学中的距离进行量化分析，使用统计算法Bray-Curtis计算两两样本间距离，获得距离矩阵，可用于后续进一步的beta多样性分析和可视化统计分析。将距离矩阵使用热图表示可直观观察样本间的差异高低分布。

将不同分类或环境的多组样本的距离进行四分位计算，比较不同样本组的组内和组间的距离分布差异。同时进行multiple
Student’s two-sample t-tests判断样本组间的显著性差异。

箱式图的作用：识别数据异常值；粗略估计和判断数据特征；比较几批数据的形状，同一数轴上，几批数据的箱形图并行排列，几批数据的中位数、尾长、异常值、分布区间等形状信息一目了然。

箱线图（Boxplot）也称箱须图（Box-whisker
Plot）[28]，是利用数据中的五个统计量：最小值、第一四分位数、中位数、第三四分位数与最大值来描述数据的一种方法，它也可以粗略地看出数据是否具有对称性，分布的分散程度等信息，特别可以用于对几个样本的比较。
简单箱线图由五部分组成，分别是最小值、中位数、最大值和两个四分位数

结果目录： 02.Distance/

\*otu\_table.txt.all.pdf： 样本间距离heatmap图

**Heatmap analysis for Distance matrix**

注：图中热图不同颜色表示的值是样品两两之间的差异系数，差异系数越小的两个样品，物种多样性的差异越小。

Group\_Distances.pdf： 组间组内距离箱式图

**boxplot for group distance**

注：第一四分位数 (Q1)，又称“下四分位数”，等于该样本中所有数值由小到大排列后第25%的数字。第二四分位数
(Q2)，又称“中位数”，等于该样本中所有数值由小到大排列后第50%的数字。 第三四分位数 (Q3)，又称“上四分位数”，等于该样本中所有数值由小到大排列后第75%的数字。

### 二 、 PCA分析

#### 分析方法

PCA分析(Principal Component
Analysis)[12]，即主成分分析，是一种对数据进行简化分析的技术，这种方法可以有效的找出数据中最“主要”的元素和结构，去除噪音和冗余，将原有的复杂数据降维，揭示隐藏在复杂数据背后的简单结构。其优点是简单且无参数限制。通过分析不同样本OTU（97%相似性）组成可以反映样本间的差异和距离，PCA运用方差分解，将多组数据的差异反映在二维坐标图上，坐标轴取能够最大反映方差值的两个特征值。如样本组成越相似，反映在PCA图中的距离越近。不同环境间的样本可能表现出分散和聚集的分布情况，PCA结果中对样本差异性解释度最高的两个或三个成分可以用于对假设因素进行验证。

软件：R语言PCA统计分析和作图。

#### 结果说明

结果目录： 03.PCA/

pca.sites.xls：记录了样本在各个维度上的位置，其中PC1为x轴，PC2为y轴，依此类推。

pca\_rotation.xls：记录了每个OTU对各主成分的贡献度。

pca\_importance.xls：记录了各维度解释结果的百分比。如果PC1值为50%，则表示x轴的差异可以解释全面分析结果的50%。

pca\_pc\*.pdf : PCA图

3d-pca\_pc1-2-3.pdf : 3维PCA图

**Multiple samples PCA analysis**

**Multiple samples 3D PCA analysis**

注：结果文件中的pc1-2，pc1-3，pc2-3分别是用前三个主要成分两两组合，分别进行了作图。不同颜色或形状的点代表不同环境或条件下的样本组，横、纵坐标轴的刻度是相对距离，无实际意义。PC1、PC2分别代表对于两组样本微生物组成发生偏移的疑似影响因素，需要结合样本特征信息归纳总结，例如A组（红色）和B组（蓝色）样本在pc1轴的方向上分离开来，则可分析为PC1是导致A组和B组分开（可以是两个地点或酸碱不同）的主要因素，同时验证了这个因素有较高的可能性影响了样本的组成。

### 三 、 PCOA分析

#### 分析方法

PCOA分析，即主坐标分析（principal co-ordinates
analysis），也是一种非约束性的数据降维分析方法，可用来研究样本群落组成的相似性或差异性，与PCA分析类似；主要区别在于，PCA基于欧氏距离，PCoA基于除欧氏距离以外的其它距离，通过降维找出影响样本群落组成差异的潜在主成分。

PCOA分析，首先对一系列的特征值和特征向量进行排序，然后选择排在前几位的最主要特征值，并将其表现在坐标系里，结果相当于是距离矩阵的一个旋转，它没有改变样本点之间的相互位置关系，只是改变了坐标系统。

软件：R语言PCOA统计分析和作图。

#### 结果说明

结果目录： 04.PCOA/bray\_curtis

pcoa\_\*\_otu\_table.txt\_sites：记录了样本在各个维度上的位置，其中Pco1为x轴，Pco2为y轴，依此类推。

pcoa\_\*\_otu\_table.txt\_rotation：记录了每个OTU对各主成分的贡献度。

pcoa\_\*\_otu\_table.txt\_importance：记录了各维度解释结果的百分比。如果PC1值为50%，则表示x轴的差异可以解释全面分析结果的50%。

pcoa\_\*\_otu\_table.txt\_pc\*.pdf: PCOA图

3d-pcoa\_\*pc1-2-3.pdf : 3维PCOA图

**Multiple samples PCOA analysis**

**Multiple samples 3D PCoA analysis**

注：结果文件中的pc1-2，pc1-3，pc2-3分别是用前三个主要成分两两组合，分别进行了作图。不同颜色或形状的点代表不同环境或条件下的样本组，横、纵坐标轴的刻度是相对距离，无实际意义。PC1、PC2分别代表对于两组样本微生物组成发生偏移的疑似影响因素，需要结合样本特征信息归纳总结，例如A组（红色）和B组（蓝色）样本在PC1轴的方向上分离开来，则可分析为PC1是导致A组和B组分开（可以是两个地点或酸碱不同）的主要因素，同时验证了这个因素有较高的可能性影响了样本的组成。

### 四 、 基于UniFrac的PCOA分析

#### 分析方法

Unifrac分析得到的距离矩阵可用于多种分析方法，可通过多变量统计学方法PCoA分析，直观显示不同环境样本中微生物进化上的相似性及差异性。

PCOA（principal co-ordinates
analysis）[16]是一种研究数据相似性或差异性的可视化方法，通过一系列的特征值和特征向量进行排序后，选择主要排在前几位的特征值，PCoA可以找到距离矩阵中最主要的坐标，结果是数据矩阵的一个旋转，它没有改变样本点之间的相互位置关系，只是改变了坐标系统。

UnifracPCoa基于进化距离，在进化水平上挖掘影响样品群落组成差异的潜在主成分。

分析软件：R语言PCoA分析和作PCoA图。

#### 结果说明

结果目录： 04.PCOA/weighted\_unifrac/
04.PCOA/unweighted\_unifrac/

pcoa\_\*weighted\_unifrac\_dm.txt\_sites：记录了样本在各个维度上的位置，其中Pco1为x轴，Pco2为y轴，依此类推。

pcoa\_\*weighted\_unifrac\_dm.txt\_rotation：记录了每个OTU对各主成分的贡献度。

pcoa\_\*weighted\_unifrac\_dm.txt\_importance：记录了各维度解释结果的百分比。如果PC1值为50%，则表示x轴的差异可以解释全面分析结果的50%。

pcoa\_\*weighted\_unifrac\_dm.txt\_pc\*.pdf：(un)weighted\_Unifrac\_PCoA图

3d-pcoa\_\*pc1-2-3.pdf : (un)weighted\_Unifrac 3维PCOA图

**(un)weighted unifrac PCoA analysis**

**(un)weighted unifrac 3D PCoA analysis**

注：结果文件中的pc1-2，pc1-3，pc2-3分别是用前三个主要成分两两组合，分别进行了作图。不同颜色或形状的点代表不同环境或条件下的样本组，横、纵坐标轴的刻度是相对距离，无实际意义。PC1、PC2分别代表对于两组样本微生物组成发生偏移的疑似影响因素，需要结合样本特征信息归纳总结，例如A组（红色）和B组（蓝色）样本在PC1轴的方向上分离开来，则可分析为PC1是导致A组和B组分开（可以是两个地点或酸碱不同）的主要因素，同时验证了这个因素有较高的可能性影响了样本的组成。

### 五 、 基于Beta多样性距离的非度量多维尺度分析(NMDS)

### 5.1 基于Beta多样性距离的NMDS分析

#### 分析方法

非度量多维尺度法[17]是一种将多维空间的研究对象（样本或变量）简化到低维空间进行定位、分析和归类，同时又保留对象间原始关系的数据分析方法。适用于无法获得研究对象间精确的相似性或相异性数据，仅能得到他们之间等级关系数据的情形。其基本特征是将对象间的相似性或相异性数据看成点间距离的单调函数，在保持原始数据次序关系的基础上，用新的相同次序的数据列替换原始数据进行度量型多维尺度分析。换句话说，当资料不适合直接进行变量型多维尺度分析时，对其进行变量变换，再采用变量型多维尺度分析，对原始资料而言，就称之为非度量型多维尺度分析。其特点是根据样本中包含的物种信息，以点的形式反映在多维空间上，而对不同样本间的差异程度，则是通过点与点间的距离体现的，最终获得样本的空间定位点图。

软件：Qiime计算beta多样性距离矩阵，R语言vegan软件包作NMDS分析和作图。

#### 结果说明

结果目录： 05.NMDS/bray\_curtis/

nmds\_bray\_cruist\*.xls：记录了样本在坐标空间中各个维度上的位置；

nmds\_bray\_cruist\*.pdf：NMDS分析图。

 **NMDS analysis**

注：不同颜色或形状的点代表不同环境或条件下的样本组。

### 5.2 基于UniFrac的NMDS分析

#### 分析方法

Unifrac分析得到的距离矩阵可用于多种分析方法，可通过多变量统计学方法Nmds分析，直观显示不同环境样本中微生物进化上的相似性及差异性。

非度量多维尺度法[17]是一种将多维空间的研究对象（样本或变量）简化到低维空间进行定位、分析和归类，同时又保留对象间原始关系的数据分析方法。适用于无法获得研究对象间精确的相似性或相异性数据，仅能得到他们之间等级关系数据的情形。其基本特征是将对象间的相似性或相异性数据看成点间距离的单调函数，在保持原始数据次序关系的基础上，用新的相同次序的数据列替换原始数据进行度量型多维尺度分析。换句话说，当资料不适合直接进行变量型多维尺度分析时，对其进行变量变换，再采用变量型多维尺度分析，对原始资料而言，就称之为非度量型多维尺度分析。其特点是根据样本中包含的物种信息，以点的形式反映在多维空间上，而对不同样本间的差异程度，则是通过点与点间的距离体现的，最终获得样本的空间定位点图。

Unifrac基于进化距离，在进化水平上挖掘影响样品群落组成差异的程度。

软件：unifrac计算beta多样性距离矩阵，R语言vegan软件包作NMDS分析和作图。

#### 结果说明

结果目录： 05.Nmds/weighted\_unifrac/
05.Nmds/unweighted\_unifrac/

nmds\_\*weighted\_unifrac\*\_sites.xls：记录了样本在坐标空间中各个维度上的位置；

nmds\_\*weighted\_unifrac\*.pdf：NMDS分析图。

 **NMDS analysis**

注：不同颜色或形状的点代表不同环境或条件下的样本组。

### 六 、 多样本相似度树状图

#### 分析方法

利用树枝结构描述和比较多个样本间的相似性和差异关系。首先使用描述群落组成关系和结构的算法计算样本间的距离，即根据beta多样性距离矩阵进行层次聚类（Hierarchical
cluatering）分析[16]，使用非加权组平均法UPGMA（Unweighted pair group method with arithmetic
mean）算法构建树状结构，得到树状关系形式用于可视化分析。

软件：Qiime计算beta多样性距离矩阵，计算距离矩阵的算法为bray curtis

其中

SA,i = 表示A样本中第i个OTU所含的序列数；

SB,i = 表示B样本中第i个OTU所含的序列数。

最后用R语言作图画树。

#### 结果说明

结果目录： 06.Hcluster\_tree/bray\_curtis/

hcluster\_tree\_bray\_cruist.tre：newick-formatted树文件；newick是一种树状的标准格式文件，可被多种建树软件识别，例如：PHYLIP、TREEVIEW、ARB。

newick 格式：

(((SAMP1:0.02120,(SAMP2:0.09111,SAMP3:0.04491)node1:0.00097)node2:0.00194,(SAMP4:0.03160,SAMP5:0.04378)node3:0.00365)node4:0.00188,SAMP6:0.00881)node5:0.00739；
其中括号内聚到一起相似的树枝，冒号后为距离。

hcluster\_tree\_bray\_cruist.pdf：多样本相似度树状图

**Multiple samples similarity tree**

注：树枝长度代表样本间的距离，样本可按预先的分组以不同颜色区分，末端竖线表示样本聚在一起相似度较高。

### 七 、 基于UniFrac的聚类树分析

#### 分析方法

Unifrac分析得到的距离矩阵可用于多种分析方法，通过层次聚类（Hierarchical
cluatering）[17]中的非加权组平均法UPGMA构建进化树等图形可视化处理，可以直观显示不同环境样本中微生物进化上的相似性及差异性。

UPGMA（Unweighted pair group method with arithmetic
mean）假设在进化过程中所有核苷酸/氨基酸都有相同的变异率，即存在着一个分子钟。通过树枝的距离和聚类的远近可以观察样本间的进化距离。分析软件： R语言vegan包UPGMA分析和作进化树。

#### 结果说明

结果目录： 06.Hculster\_tree/weighted\_unifrac/
06.Hcluster\_tree/unweighted\_unifrac/

\*\_unifrac.pdf : 样本进化树分析图

**(un) weighted unifrac tree analysis**

注：树枝颜色为预先定义的不同分组标注。

### 八 、 ANOSIM相似性分析

#### 分析方法

相似性分析(ANOSIM)是一种非参数检验，用来检验组间（两组或多组）的差异是否显著大于组内差异，从而判断分组是否有意义。首先利用Bray-Curtis算法计算两两样品间的距离，然后将所有距离从小到大进行排序，按以下公式计算R值，之后将样品进行置换，重新计算R\*值，R\*大于R的概率即为P值。

其中， ---- 表示组间（Between
groups）距离排名的平均值；

---- 表示组内（Within
groups）距离排名的平均值；

          n
---- 表示样品总数。

使用软件：R语言vegan包或QIIME软件

#### 结果说明

结果目录： 07.Anosim/

anosim\_results.txt：组间相似性分析结果

**Anosim analysis**

注：理论上，R值范围为-1到+1，实际中R值一般从0到1。R值接近1表示组间差异越大于组内差异，R值接近0则表示组间和组内没有明显差异；此次统计分析的可信度用P-value表示，
P< 0.05表示统计具有显著性；Number of permutation表示置换次数。

### 九 、 Adonis多因素方差分析

#### 分析方法

Adonis又称置换多因素方差分析（permutational MANOVA）或非参数多因素方差分析（nonparametric
MANOVA）。它利用半度量(如Bray-Curtis) 或度量距离矩阵(如Euclidean)对总方差进行分解，分析不同分组因素对样品差异的解释度，并使用置换检验对划分的统计学意义进行显著性分析。

使用软件：R语言vegan包或QIIME软件

结果目录： 08.Adonis/

adonis\_results.txt：Adonis分析结果

**permutational MANOVA analysis**

Call:

adonis(formula = as.dist(qiime.data$distmat) ~ qiime.data$map[[opts$category]], permutations = opts$num\_permutations)

Permutation: free

Number of permutations: 999

Terms added sequentially (first to last)

注：category --- 表示分组标准；

Df --- 表示自由度；

SumsOfSqs --- 总方差，又称离差平方和；

MeanSqs --- 均方（差），即SumsOfSqs/Df；

F.Model ---- F检验值；

R2 --- 表示不同分组对样品差异的解释度，即分组方差与总方差的比值，R2越大表示分组对差异的解释度越高；

Pr --- 表示P值，小于0.05说明本次检验的可性度高。

## 差异分析

### 一 、 LEfSE分析

#### 分析方法

LEfSe[30]是一种用于发现高维生物标识和揭示基因组特征的软件。包括基因，代谢和分类，用于区别两个或两个以上生物条件（或者是类群）。该算法强调的是统计意义和生物相关性。让研究人员能够识别不同丰度的特征以及相关联的类别。

LEfSe通过生物学统计差异使其具有强大的识别功能。然后，它执行额外的测试，以评估这些差异是否符合预期的生物学行为。具体来说，首先使用non-parametric factorial
Kruskal-Wallis (KW) sum-rank
test（非参数因子克鲁斯卡尔—沃利斯和秩验检）检测具有显著丰度差异特征，并找到与丰度有显著性差异的类群。最后，LEfSe采用线性判别分析（LDA）来估算每个组分（物种）丰度对差异效果影响的大小。

分析软件：LEfSE[31]（http://huttenhower.sph.harvard.edu/galaxy/root?tool\_id=lefse\_upload）根据分类学组成对样本按照不同的分组条件进行线型判别分析（LDA），找出对样本划分产生显著性差异影响的群落或物种。

#### 结果说明

结果目录： 01.Lefse/

lefse\_LDA.xls : LDA判别分析结果；

lefse\_LDA.pdf : LDA分析柱图；

lefse\_LDA.cladogram.pdf：进化分枝图。

注：图为聚类树，红色区域和绿色区域表示不同分组，树枝中红色节点表示在红色组别中起到重要作用的微生物类群，绿色节点表示在绿色组别中起到重要作用的微生物类群，黄色
节点表示的是在两组中均没有起到重要作用的微生物类群。图中英文字母表示的物种名称在右侧图例中进行展示。

 **Plot LEfSe Result**

注：图为统计两个组别当中有显著作用的微生物类群通过LDA分析（线性回归分析）后获得的LDA分值。在lefse\_LDA.xls
表中：第一列为物种名称；第二列为Abundance丰度值，为差异组的丰度；第三列为Group，展示差异组名；第四列为LDA值，第五列为P值。默认LDA值大于2，P值小于0.05，该物种为差异物种，差异组即两组中（或多组中）丰度高的一组。若Group、LDA、P值均为空，则表示该物种在组间无差异，即无差异物种。

### 二 、 ANOVA方差分析

#### 分析方法

ANOVA方差分析又称F检验，通过分析研究不同来源的变异对总变异的贡献大小，从而确定可控因素对研究结果的影响力。其基本原理是认为不同处理组的差别来源有两个：

(1)实验条件。即不同处理造成的差异，称为组间差异。用变量在各组的均值与总均值之偏差平方和的总和表示。

(2)随机误差。如测量造成的差异或个体间的差异，称为组内差异，用变量在各组的均值与该组内变量值之偏差平方和的总和表示。

组间差异、组内差异除以各自的自由度，得到均方差MSb和MSw。MSw/MSb越接近1，表示各组样本均来自同一总体，说明处理没有作用；反之越接近0，说明差异主要是由于不同处理造成的，即各样本来自于不同的总体，说明处理具有明显作用。可选分类学水平：OTU、门、纲、目、科、属、种，默认OTU水平。

#### 结果说明

结果目录： 02.Anova/

anova.txt：anova方差分析结果

**表 差异丰富度特征分析**

OTU：OTU编号； Test-Statistic：统计检验值

P：原始p值；FDR\_P：FDR校正后的p值；Bonferroni\_P：Bonferroni校正后的p值

Group1\_mean：第一组的均值；Group2\_mean：第二组的均值；Group3\_mean：第三组的均值

Taxonomy：对应的分类学信息

### 三 、 Wilcoxon秩和检验分析

#### 分析方法

Wilcoxon rank-sum test，也叫曼-惠特尼U检验（Mann–Whitney U
test），是两组独立样本非参数检验的一种方法。其原假设为两组独立样本来自的两总体分布无显著差异，通过对两组样本平均秩的研究来实现判断两总体的分布是否存在差异。可选分类学水平：OTU、门、纲、目、科、属；默认门和属水平

#### 结果说明

结果目录： 03.Wilcoxon/

wilcox.\*txt：wilcoxon秩和检验分析结果

**表 Wilcox 秩和检验分析结果**

OTU：OTU编号（或其他水平物种名称）；

mean(group1)：第一组的均值；sd(group1)：第一组的标准差；

mean(group2)：第二组的均值；sd(group2)：第二组的标准差；

p-value：p值，p-value < 0.05表示统计具有显著性；

q-value：q值，用来表示此次统计分析的可信度，小于0.05说明本次检验的可信度高。

## 附录：

### 一 、 文档结构

注意：原始数据文件虽然是文本格式，但是解压缩后一般体积较大，不适合在Windows 下直接打开。

  
  
  

### 二 、 文件打开或浏览方法

所有提供的文件均为Linux系统下的文件，压缩包使用“tar –zcvf
”命令压缩，以下为不同系统用户解压缩的方法：

Unix/Linux/Mac用户: 使用tar –zcvf \*.tar.gz命令

Windows用户：使用WinRAR软件解压缩

如在本附录中无特殊说明，所有提供的文件均为Liux系统下文本文件。Unix/Linux用户可以使用more或less命令查看该文本文件内容；Windows用户，一般文本文件可以使用写字板打开。推荐使用开源文本编辑器gedit
for win32版本(http://projects.gnome.org/gedit/)或者notepad。当文件比较大时，打开文件可能导致Windows系统死机，建议使用性能较好的计算机或者使用更适合处理大量数据的Unix/Linux系统打开。

数据中可能包含部分图像文件，一般图像文件后缀名为.png、.pdf、.gif、tiff等，对于图像文件，Windows用户可以使用图片浏览器打开，Linux/Unix用户使用display命令打开。
公司默认提供“pdf”格式的矢量图，可利用"Adobe Illustrator"软件对该格式图片进行编辑。

Linx下的表格均为制表符(Tab)分割的文本，为了便于阅读，建议使用excel或openoffice等办公软件用表格形式打开，打开时请用“Tab分割”方式。

### 三 、 分析方法说明（英文）

### 3.1 Sample Collection（此部分视实际项目情况撰写）

### 3.2 DNA extraction and PCR amplification

Microbial DNA was extracted from xx samples using
the E.Z.N.A.® Soil（人粪便换为Stool） DNA Kit (Omega Bio-tek, Norcross, GA, U.S.) according to
manufacturer’s protocols. The V4-V5 region of the bacteria 16S ribosomal RNA gene were amplified
by PCR (95 °C for 2 min, followed by 25 cycles at 95 °C for 30 s, 55 °C for 30 s, and 72 °C for
30 s and a final extension at 72 °C for 5 min) using primers 515F 5’-barcode- GTGCCAGCMGCCGCGG)-3’
and 907R 5’-CCGTCAATTCMTTTRAGTTT-3’, where barcode is an eight-base sequence unique to each sample.
PCR reactions were performed in triplicate 20 μL mixture containing 4 μL of 5 × FastPfu Buffer,
2 μL of 2.5 mM dNTPs, 0.8 μL of each primer (5 μM), 0.4 μL of FastPfu Polymerase, and 10 ng of
template DNA. Amplicons were extracted from 2% agarose gels and purified using the AxyPrep DNA Gel
Extraction Kit (Axygen Biosciences, Union City, CA, U.S.) according to the manufacturer’s
instructions.

### 3.3 Library Construction and Sequencing

Purified PCR products were quantified by Qubit®3.0 (Life
Invitrogen) and every twenty-four amplicons whose barcodes were different were mixed equally. The
pooled DNA product was used to construct Illumina Pair-End library following Illumina’s genomic DNA
library preparation procedure. Then the amplicon library was paired-end sequenced (2 × 250) on an
Illumina MiSeq platform (Shanghai BIOZERON Co., Ltd ) according to the standard protocols. The raw
reads were deposited into the NCBI Sequence Read Archive (SRA) database (Accession Number:
SRP\*\*\*\*\*\*).

### 3.4 Processing of sequencing data

Raw fastq files were first demultiplexed using in-house perl
scripts according to the barcode sequences information for each sample with the following criteria:
(i) The 250bp reads were truncated at any site receiving an average quality score <20 over a 10 bp
sliding window, discarding the truncated reads that were shorter than 50bp. (ii) exact barcode matching,
2 nucleotide mismatch in primer matching, reads containing ambiguous characters were removed. (iii) only
sequences that overlap longer than 10 bp were assembled according to their overlap sequence. Reads which
could not be assembled were discarded.

#### Method 2 (dada2) -- Ribosomal Sequence Variants (RSVs) Generation

Passed sequences were dereplicated and subjected to the DADA2
algorithm (QIIME 2 recommended) to identify indel-mutations and substitutions [4]. The trimming and
filtering were performed on paired reads with a maximum of two expected errors per read (maxEE=2). After
merging paired reads and chimera filtering, the phylogenetic affiliation of each 16S rRNA gene sequence
(herein called RSVs) was analyzed by uclust algorithm (http://www.drive5.com/usearch/manual/uclust\_algo.html)
against the silva (SSU138.1)16S rRNA database using confidence threshold of 80% [1].

#### Method 3 (deblur) -- Operational Units (OTUs) Generation

Sequences were clustered into operational taxonomic units
(OTUs) at 100% similarity (identical) using the Deblur denoising algorithm, which removes noise due to
sequencing error [5]. Clusters of identical sequences allowed us to detect microbial changes at fine
scale resolution. The phylogenetic affiliation of each 16S rRNA gene sequence was analyzed by uclust
algorithm (http://www.drive5.com/usearch/manual/uclust\_algo.html) against the silva (SSU138.1)16S
rRNA database using confidence threshold of 80% [1].

### 3.5 Alpha- and Beta-diversity Analyses (根据实际使用的结果，选择性使用对应的方法介绍)

The rarefaction analysisbased on Mothur v.1.21.1[2] was
conducted to reveal the diversity indices, including the Chao, ACE, and Shannon diversity indices. The
beta diversity analysis was performed using UniFrac [3] to compare the results of the principal component
analysis (PCA) using the community ecology package, R-forge (Vegan 2.0 package was used to generate a PCA
figure). Mantel tests were carried out to examine the Spearman’s rank correlation between the XXXX factor
and the bacterial community similarity using Bray-Curtis distance matrices with 999 permutations, using
the vegan package in R. Multivariate analysis of variance (MANOVA) was conducted to further confirm the
observed differences. The Spearman’s correlation coefficients were assessed to determine the relationships
between microbiota and chemical factor such as signaling molecules. Correlation was considered significant
when the absolute value of Spearman’s rank correlation coefficient (Spearman’s r) was >0.6 and statistically
significant (P < 0.05). All statistic analysis were performed by R stats package. The R (pheatmap package)
and Cytoscape (http://www.cytoscape.org）were applied to visualize the relationships through correlation
heatmap and network diagrams respectively. Redundancy analysis (RDA) was employed to explore the relationship
between environmental factors and bacterial communities. One way analysis of variance (ANOVA) tests were
performed to assess the statistically significant difference of diversity indices between samples. Differences
were considered significant at p < 0.05. Venn diagrams were drawn using online tool “Draw Venn Diagram”
(http://bioinformatics.psb.ugent.be/webtools/Venn) to analyze overlapped and unique OTUs during the treatment
processes. One-way permutational analysis of variance (PERMANOVA) was performed using R vegan package to assess
the statistically significant effects of treatment processes on bacterial communities
(Anderson and Walsh, 2013).

### 3.6 LEfSe Analysis

For identification of biomarkers for highly dimensional colonic
bacteria, LEfSe (linear discriminant analysis effect size) analysis was done [6]. Kruskal-Wallis sum-rank
test was performed to examine the changes and dissimilarities among classes followed by LDA analysis to
determine the size effect of each distinctively abundant taxa [7].

### 3.7 Functional prediction of the microbial genes

Phylogenetic Investigation of Communities by Reconstruction of
Unobserved States (PICRUSt) (http://picrust.github.io/picrust/tutorials/genome\_prediction.html) program based
on the Kyoto Encyclopedia of Genes and Genomes (KEGG) database was used to predict the functional alteration
of microbiota in different samples. The OTU data obtained were used to generate BIOM files formatted as input
for PICRUSt v1.1.09 with the make.biom script usable in the Mothur. OTU abundances were mapped to Greengenes OTU
IDs as input to speculate about the functional alteration of microbiota.

### 3.8 Reference（分析方法说明部分参考文献）

[1] Katherine R Amato, Carl J Yeoman, Angela Kent, et al. Habitat degradation
impacts black howler monkey (Alouatta pigra) gastrointestinal microbiomes. The ISME Journal (2013) 7,1344–1353.

[2] Schloss PD et al (2009) Introducing mothur: open-source,
platform-independent, community-supported software for describing and comparing microbial communities. Appl Environ
Microbiol 75:7537–7541

[3] Lozupone C, Lladser ME, Knights D, Stombaugh J, Knight R (2011) UniFrac:
an effective distance metric for microbial community comparison. ISME J 5:169

[4] Callahan BJ, McMurdie PJ, Rosen MJ, Han AW, Johnson AJ, Holmes SP. (2016a).
DADA2: high-resolution sample inference from Illumina amplicon data. Nat Methods 13: 581–583.

[5] Amir A, McDonald D, Navas-Molina JA, Kopylova E, Morton JT, Zech Xu Z,
Kightley EP, Thompson LR, Hyde ER, Gonzalez A, et al. 2017. Deblur rapidly resolves single-nucleotide community
sequence patterns. mSystems 2:e00191–16.

[6] Segata, N., Izard, J., Waldron, L., Gevers, D., Miropolsky, L., Garrett,
W. S.,et al. (2011). Metagenomic biomarker discovery and explanation. Genome Biol.12:R60.
doi: 10.1186/gb-2011-12-6-r60

[7] Ijaz M U , Ahmed M I , Zou X , et al. Beef, Casein, and Soy Proteins
Differentially Affect Lipid Metabolism, Triglycerides Accumulation and Gut Microbiota of High-Fat Diet-Fed C57BL/6J
Mice[J]. Frontiers in Microbiology, 2018, 9.

### 四 、 参考文献

[1] Caporaso JG, Kuczynski J. et al. QIIME allows analysis of
high-throughput community sequencing data. Nature Methods 7(5): 335-336(2010).

[2] Edgar, R.C, Haas,BJ. et al. UCHIME improves sensitivity
and speed of chimera detection, Bioinformatics doi: 10.1093/bioinformatics/btr381 [PMID
21700674](2011).

[3] Edgar, R.C. (2013) UPARSE: Highly accurate OTU sequences
from microbial amplicon reads, Nature Methods [Pubmed:23955772, dx.doi.org/10.1038/nmeth.2604].

[4] Quast C, Pruesse E, Yilmaz P, Gerken J, Schweer T, Yarza
P, Peplies J, Glöckner FO . The SILVA ribosomal RNA gene database project: improved data processing
and web-based tools. Nucl. Acids Res. 41 (D1): D590-D596(2013).

[5] Cole, J. R., Q. Wang, E. Cardenas, J. Fish, B. Chai, R.
J. Farris, A. S. Kulam-Syed-Mohideen, D. M. McGarrell, T. Marsh, G. M. Garrity, and J. M. Tiedje.
The Ribosomal Database Project: improved alignments and new tools for rRNA analysis. Nucleic Acids
Res. 37 (Database issue): D141-145;doi: 10.1093/nar/gkn879] [PMID: 19004872] (2009).

[6] DeSantis, T. Z., P. Hugenholtz, N. Larsen, M. Rojas, E.
L. Brodie, K. Keller, T. Huber, D. Dalevi, P. Hu, and G. L. Andersen. 2006. Greengenes, a
Chimera-Checked 16S rRNA Gene Database and Workbench Compatible with ARB. Appl Environ Microbiol
72:5069-72.

[7] Urmas Kõljalg, R. Henrik Nilsson, Kessy Abarenkov, Leho
Tedersoo, et al. Towards a unified paradigm for sequence-based identification of fungi. Molecular
Ecology (2013)22,5271–5277DOI: 10.1111/mec.12481

[8] Jordan A. Fish, Benli Chai, Qiong Wang, Yanni Sun, C.
Titus Brown, James M. Tiedje and James R. Cole , FunGene: the functional gene pipeline and
repository. Front. Microbiol., 01 October 2013 | doi: 10.3389/fmicb.2013.00291

[9] Wang Q, Garrity GM, Tiedje JM, Cole JR. 2007. Naive
Bayesian classifier for rapid assignment of rRNA sequences into the new bacterial taxonomy.Appl
Environ Microb 73(16): 5261-5267.

[10] Schloss PD, Gevers D, Westcott SL (2011) Reducing the
Effects of PCR Amplification and Sequencing Artifacts on 16S rRNA-Based Studies. PLoS ONE 6(12):
e27310. doi:10.1371/journal.pone.0027310.

[11] Katherine R Amato. et al. Habitat degradation impacts
black howler monkey (Alouatta pigra) gastrointestinal microbiomes. The ISME Journal (2013) 7,
1344–1353; doi:10.1038/ismej.

[12] Yu Wang, Hua-Fang Sheng, et al. Comparison of the Levels
of Bacterial Diversity in Freshwater,Intertidal Wetland, and Marine Sediments by Using Millions of
Illumina Tags. Appl. Environ. Microbiol. 2012, 78(23):8264. DOI: 10.1128/AEM.01821-12.

[13] Scott T Bates, Jose C Clemente, et al. Global
biogeography of highly diverse protistan communities in soil. The ISME Journal (2013) 7, 652–659;
doi:10.1038/ismej.2012.147.

[14] Maughan H, Wang PW, Diaz Caballero J, Fung P, Gong Y, et
al. (2012) Analysis of the Cystic Fibrosis Lung Microbiota via Serial Illumina Sequencing of
Bacterial 16S rRNA Hypervariable Regions. PLoS ONE 7(10): e45791.
doi:10.1371/journal.pone.0045791.

[15] Fouts DE, Szpakowski S, Purushe J, Torralba M, Waterman
RC, et al. (2012) Next Generation Sequencing to Define Prokaryotic and Fungal Diversity in the
Bovine Rumen. PLoS ONE 7(11): e48289. doi:10.1371/journal.pone.0048289.

[16] Xiao-Tao Jiang ,Xin Peng, et al.Illumina Sequencing of
16S rRNA Tag Revealed Spatial Variations of Bacterial Communities in a Mangrove Wetland. Microb Ecol
(2013) 66:96–104.DOI 10.1007/s00248-013-0238-8.

[17] Magali Noval Rivas, PhD, Oliver T. Burton, et al. A
microbita signature associated with experimental food allergy promotes allergic senitization and
anaphylaxis. The Journal of Allergy and Clinical Immunology.Volume 131, Issue 1 , Pages 201-212,
January 2013.

[18] Sheik CS, Mitchell TW, Rizvi FZ, Rehman Y, Faisal M, et
al. (2012) Exposure of Soil Microbial Communities to Chromium and Arsenic Alters Their Diversity and
Structure. PLoS ONE 7(6): e40059. doi:10.1371/journal.pone.0040059.

[19] Lisa Oberauner, Christin Zachow, Stefan Lackner, et al.
The ignored diversity: complex bacterialcommunities in intensive care units revealed by 16S
pyrosequencing. SCIENTIFIC REPORTS. 3 :1413 .DOI: 10.1038/srep01413.(2013)

[20] Srinivasan S, Hoffman NG, Morgan MT, Matsen FA, Fiedler
TL, et al. (2012) Bacterial Communities in Women with Bacterial Vaginosis: High Resolution
Phylogenetic Analyses Reveal Relationships of Microbiota to Clinical Criteria. PLoS ONE 7(6):
e37818. doi:10.1371/journal.pone.0037818.

[21] Elie Jami, Adi Israel, et al. Exploring the bovine rumen
bacterial community from birth to adulthood. The ISME Journal advance online publication, 21
February 2013; doi:10.1038/ismej.2013.2

[22] Huson, DH, Mitra, S, Weber, N, Ruscheweyh, H, and
Schuster, SC (2011). Integrative analysis of environmental sequences using MEGAN4. Genome Research,
21:1552-1560.

[23] Tingting Wang, Guoxiang Cai, et al. Structural
segregation of gut microbiota between colorectal cancer patients and healthy volunteers. The ISME
Journal advance online publication, 18 August 2011; doi:10.1038/ismej.2011.109.

[24] Laurent Philippot, et al. Loss in microbial diversity
affects nitrogen cycling in soil. The ISME Journal (2013) 7, 1609–1619;
doi:10.1038/ismej.2013.34.

[25] Price MN, Dehal PS, Arkin AP (2010) FastTree 2 –
Approximately Maximum-Likelihood Trees for Large Alignments. PLoS ONE 5(3): e9490.
doi:10.1371.journal.pone.0009490.

[26] Tanya Yatsunenko, Federico, et al. Human gut microbiome
viewed across age and geography. Nature 486, 222–227 (14 June 2012) doi:10.1038.nature11053.

[27] Micah Hamady, Catherine Lozupone and Rob Knight. Fast
UniFrac: facilitating high-throughput phylogenetic analyses of microbial communities including
analysis of pyrosequencing and PhyloChip data. The ISME Journal (2010) 4, 17–27;
doi:10.1038/ismej.2009.97

[28] Abrahamsson T.R., Jakobsson H.E., Andersson A.F.,
Bjorksten B., Engstrand L., Jenmalm M.C. Low diversity of the gut microbiota in infants with atopic
eczema. (2012) Journal of Allergy and Clinical Immunology, 129(2).

[29] Mark V Brown, Federico M Lauro, et al. Global
biogeography of SAR11 marine bacteria. Molecular Systems Biology 8; Article number 595;
doi:10.1038/msb.2012.28.

[30] Chenhong Zhang, Shoufeng Li, Liu Yang, et al. Structural
modulation of gut microbiota in life-longcalorie-restricted mice. NATURE
COMMUNICATIONS,4:2163,DOI:10.1038/ncomms3163(2013).

[31] Segata N, Izard J, Waldron L, Gevers D, Miropolsky L et
al. (2011) Metagenomic biomarker discovery and explanation. Genome Biol 12:
R60.10.1186/gb-2011-12-6-r60 PubMed: 21702898.

[32] White, J.R., Nagarajan, N. & Pop, M. Statistical methods
for detecting differentially abundant features in clinical metagenomic samples. PLoS Comput Biol 5,
e1000352 (2009).
