# Supplementary material for: β-sitosterol inhibits trimethylamine production by regulating the gut microbiota and attenuates atherosclerosis in ApoE–/– mice
Source: Front Cardiovasc Med. 2022 Nov 1;9:986905. doi: 10.3389/fcvm.2022.986905 (PMC9663806; doi:10.3389/fcvm.2022.986905)
Supplement: Supplementary file 1 [file Data_Sheet_1.ZIP › Source data/gut microbiota diversity analysis/CON-EXP/web/res/00.Data/clean.data.len.dist.html]

| Length | Sequences | Percent |
| --- | --- | --- |
| 1-50 | 9 | 0.00% |
| 51-100 | 131 | 0.01% |
| 101-150 | 117 | 0.01% |
| 151-200 | 33 | 0.00% |
| 201-250 | 7233 | 0.62% |
| 251-300 | 6853 | 0.59% |
| 301-350 | 3647 | 0.31% |
| 351-400 | 1824 | 0.16% |
| 401-450 | 1146645 | 98.30% |
