# Supplementary material for: β-sitosterol inhibits trimethylamine production by regulating the gut microbiota and attenuates atherosclerosis in ApoE–/– mice
Source: Front Cardiovasc Med. 2022 Nov 1;9:986905. doi: 10.3389/fcvm.2022.986905 (PMC9663806; doi:10.3389/fcvm.2022.986905)
Supplement: Supplementary file 1 [file Data_Sheet_1.ZIP › Source data/gut microbiota diversity analysis/CON-EXP/web/res/00.Data/cleanData.stat.html]

| #Sample | Sequences | Bases(bp) | AverageLength(bp) |
| --- | --- | --- | --- |
| 1 | 60687 | 25897329 | 426.74 |
| 10 | 66531 | 27791098 | 417.72 |
| 11 | 69776 | 29173162 | 418.10 |
| 12 | 63924 | 26452943 | 413.82 |
| 13 | 65038 | 27412760 | 421.49 |
| 14 | 66046 | 27877981 | 422.10 |
| 15 | 68421 | 28856187 | 421.74 |
| 16 | 62594 | 25679384 | 410.25 |
| 17 | 65515 | 27336163 | 417.25 |
| 18 | 52211 | 21521880 | 412.21 |
| 2 | 67595 | 28805298 | 426.15 |
| 3 | 65921 | 27902395 | 423.27 |
| 4 | 58136 | 24578645 | 422.78 |
| 5 | 58724 | 24983722 | 425.44 |
| 6 | 68067 | 29025746 | 426.43 |
| 7 | 64021 | 26420497 | 412.68 |
| 8 | 70301 | 29206797 | 415.45 |
| 9 | 72984 | 30105389 | 412.49 |
