# Supplementary material for: β-sitosterol inhibits trimethylamine production by regulating the gut microbiota and attenuates atherosclerosis in ApoE–/– mice
Source: Front Cardiovasc Med. 2022 Nov 1;9:986905. doi: 10.3389/fcvm.2022.986905 (PMC9663806; doi:10.3389/fcvm.2022.986905)
Supplement: Supplementary file 1 [file Data_Sheet_1.ZIP › Source data/gut microbiota diversity analysis/CON-EXP/web/res/00.Data/pdf_clean.data.len.dist.html]

| Length(bp) | Sequences | Percent |
| --- | --- | --- |
| 1-250 | 12 | 0.00% |
| 251-300 | 85 | 0.01% |
| 301-350 | 55 | 0.00% |
| 351-400 | 165 | 0.01% |
| 401-450 | 1314993 | 99.98% |
