# Supplementary material for: β-sitosterol inhibits trimethylamine production by regulating the gut microbiota and attenuates atherosclerosis in ApoE–/– mice
Source: Front Cardiovasc Med. 2022 Nov 1;9:986905. doi: 10.3389/fcvm.2022.986905 (PMC9663806; doi:10.3389/fcvm.2022.986905)
Supplement: Supplementary file 1 [file Data_Sheet_1.ZIP › Source data/gut microbiota diversity analysis/CON-EXP/web/res/00.Data/pdf_cleanData.stat.html]

| #Sample | Sequences | Bases(bp) | AverageLength(bp) |
| --- | --- | --- | --- |
| P1 | 41344 | 17016458 | 411.58 |
| P10 | 43134 | 18485877 | 428.57 |
| P11 | 29222 | 12507283 | 428.01 |
| P12 | 47821 | 20070695 | 419.70 |
| P13 | 55498 | 23524036 | 423.87 |
| P14 | 20287 | 8652863 | 426.52 |
| P15 | 55198 | 23557556 | 426.78 |
| P2 | 34001 | 13883255 | 408.32 |
| P3 | 38123 | 15928432 | 417.82 |
| P4 | 53589 | 22098433 | 412.37 |
| P5 | 50537 | 21569817 | 426.81 |
| P6 | 41290 | 17697593 | 428.62 |
| P7 | 33068 | 14174447 | 428.65 |
| P8 | 32754 | 14042756 | 428.73 |
| P9 | 41873 | 17937938 | 428.39 |
| S1 | 35620 | 15037289 | 422.16 |
| S10 | 58996 | 24835097 | 420.96 |
| S11 | 49126 | 20755529 | 422.50 |
| S12 | 57027 | 24202557 | 424.41 |
| S13 | 56044 | 23775603 | 424.23 |
| S14 | 34284 | 14511806 | 423.28 |
| S15 | 30230 | 12719122 | 420.75 |
| S2 | 46959 | 19321164 | 411.45 |
| S3 | 38612 | 16311219 | 422.44 |
| S4 | 36399 | 15348497 | 421.67 |
| S5 | 33009 | 13983387 | 423.62 |
| S6 | 58258 | 24543080 | 421.28 |
| S7 | 47041 | 20003592 | 425.24 |
| S8 | 56065 | 23715907 | 423.01 |
| S9 | 59901 | 25199787 | 420.69 |
