# Supplementary material for: β-sitosterol inhibits trimethylamine production by regulating the gut microbiota and attenuates atherosclerosis in ApoE–/– mice
Source: Front Cardiovasc Med. 2022 Nov 1;9:986905. doi: 10.3389/fcvm.2022.986905 (PMC9663806; doi:10.3389/fcvm.2022.986905)
Supplement: Supplementary file 1 [file Data_Sheet_1.ZIP › Source data/gut microbiota diversity analysis/CON-EXP/web/res/01.OTU_Taxa/normalize/layer.html]

| phylum | class | order | family | genus | species | otu |
| --- | --- | --- | --- | --- | --- | --- |
| Acidobacteriota | 1 | 1 | 0 | 0 | 1 | 1 |
| Actinobacteriota | 2 | 8 | 14 | 22 | 17 | 41 |
| Bacteroidota | 1 | 3 | 11 | 17 | 25 | 260 |
| Campylobacterota | 1 | 1 | 2 | 2 | 5 | 6 |
| Chloroflexi | 1 | 1 | 1 | 0 | 1 | 1 |
