# Supplementary material for: β-sitosterol inhibits trimethylamine production by regulating the gut microbiota and attenuates atherosclerosis in ApoE–/– mice
Source: Front Cardiovasc Med. 2022 Nov 1;9:986905. doi: 10.3389/fcvm.2022.986905 (PMC9663806; doi:10.3389/fcvm.2022.986905)
Supplement: Supplementary file 1 [file Data_Sheet_1.ZIP › Source data/gut microbiota diversity analysis/CON-EXP/web/res/01.OTU_Taxa/normalize/otu_table.html]

| OTU ID | 7 | 8 | 9 | 10 | 11 | 12 | 13 | 14 | 15 | 16 | 17 | 18 |
| --- | --- | --- | --- | --- | --- | --- | --- | --- | --- | --- | --- | --- |
| OTU1 | 11913 | 14254 | 21775 | 13481 | 13327 | 15294 | 242 | 456 | 225 | 403 | 234 | 411 |
| OTU2 | 784 | 67 | 21 | 471 | 482 | 161 | 306 | 222 | 207 | 289 | 427 | 1450 |
| OTU3 | 979 | 92 | 50 | 588 | 468 | 241 | 541 | 252 | 244 | 382 | 537 | 1161 |
| OTU4 | 4887 | 1317 | 1668 | 7470 | 7429 | 3389 | 7693 | 15721 | 10417 | 2883 | 11561 | 8818 |
| OTU5 | 7325 | 8471 | 14664 | 9315 | 7438 | 9608 | 74 | 218 | 99 | 235 | 108 | 125 |
