# Supplementary material for: β-sitosterol inhibits trimethylamine production by regulating the gut microbiota and attenuates atherosclerosis in ApoE–/– mice
Source: Front Cardiovasc Med. 2022 Nov 1;9:986905. doi: 10.3389/fcvm.2022.986905 (PMC9663806; doi:10.3389/fcvm.2022.986905)
Supplement: Supplementary file 1 [file Data_Sheet_1.ZIP › Source data/gut microbiota diversity analysis/CON-EXP/web/res/01.OTU_Taxa/normalize/stat.html]

| sample | domain | phylum | class | order | family | genus | species | otu |
| --- | --- | --- | --- | --- | --- | --- | --- | --- |
| all | 1 | 16 | 20 | 50 | 81 | 187 | 149 | 2590 |
| 10 | 1 | 9 | 12 | 31 | 47 | 106 | 85 | 494 |
| 11 | 1 | 10 | 13 | 32 | 48 | 104 | 77 | 494 |
| 12 | 1 | 10 | 13 | 30 | 46 | 100 | 68 | 595 |
| 13 | 1 | 10 | 13 | 31 | 49 | 120 | 80 | 655 |
