# Supplementary material for: β-sitosterol inhibits trimethylamine production by regulating the gut microbiota and attenuates atherosclerosis in ApoE–/– mice
Source: Front Cardiovasc Med. 2022 Nov 1;9:986905. doi: 10.3389/fcvm.2022.986905 (PMC9663806; doi:10.3389/fcvm.2022.986905)
Supplement: Supplementary file 1 [file Data_Sheet_1.ZIP › Source data/gut microbiota diversity analysis/CON-EXP/web/res/02.Alpha/Estimators/estimators.html]

| Sample | Reads | Chao | Richness | Shannon | Simpson | ACE | Evenness | coverage |
| --- | --- | --- | --- | --- | --- | --- | --- | --- |
| 7 | 61472 | 801.9127 | 759 | 5.0897 | 0.1011 | 846.236 | 0.531953020298151 | 0.998015356585112 |
| 8 | 69042 | 620.7875 | 527 | 4.2262 | 0.1381 | 633.2191 | 0.467420496017745 | 0.998218475710437 |
| 9 | 72164 | 569.3125 | 493 | 2.9632 | 0.2992 | 584.7401 | 0.331260628703673 | 0.998461836927 |
| 10 | 65469 | 600.4285 | 537 | 3.9264 | 0.1502 | 624.0249 | 0.432964534748227 | 0.998289266675831 |
| 11 | 68669 | 587.1376 | 527 | 3.9574 | 0.1403 | 620.542 | 0.437685700992615 | 0.998325299625741 |
| 12 | 61953 | 667.6666 | 626 | 3.7594 | 0.1645 | 714.4074 | 0.404680345311454 | 0.99796620018401 |
| 13 | 63332 | 760.2736 | 680 | 5.2613 | 0.0685 | 773.626 | 0.559161832279518 | 0.998042064043454 |
| 14 | 63939 | 736.3592 | 683 | 3.8213 | 0.1834 | 778.8969 | 0.405849803870209 | 0.997904252490655 |
| 15 | 66350 | 736.0265 | 677 | 5.0277 | 0.0902 | 758.4801 | 0.534691493760522 | 0.998251695553881 |
| 16 | 58314 | 1094.0791 | 1046 | 6.2481 | 0.0451 | 1159.9035 | 0.622904036804181 | 0.997187639331893 |
