# Supplementary material for: β-sitosterol inhibits trimethylamine production by regulating the gut microbiota and attenuates atherosclerosis in ApoE–/– mice
Source: Front Cardiovasc Med. 2022 Nov 1;9:986905. doi: 10.3389/fcvm.2022.986905 (PMC9663806; doi:10.3389/fcvm.2022.986905)
Supplement: Supplementary file 1 [file Data_Sheet_1.ZIP › Source data/gut microbiota diversity analysis/CON-EXP/web/res/02.Alpha/Rarefaction/rarefaction.html]

| numsampled | 0.97 | lci | hci |
| --- | --- | --- | --- |
| 1 | 1.0000 | 1.0000 | 1.0000 |
| 100 | 23.4900 | 18.0000 | 29.0000 |
| 200 | 34.9600 | 29.0000 | 42.0000 |
| 300 | 44.5800 | 37.0000 | 52.0000 |
| 400 | 52.7000 | 44.0000 | 61.0000 |
