# Supplementary material for: β-sitosterol inhibits trimethylamine production by regulating the gut microbiota and attenuates atherosclerosis in ApoE–/– mice
Source: Front Cardiovasc Med. 2022 Nov 1;9:986905. doi: 10.3389/fcvm.2022.986905 (PMC9663806; doi:10.3389/fcvm.2022.986905)
Supplement: Supplementary file 1 [file Data_Sheet_1.ZIP › Source data/gut microbiota diversity analysis/CON-EXP/web/res/02.Alpha/Shannon_rarefac/r_shannon.html]

| numsampled | 0.97 | lci | hci |
| --- | --- | --- | --- |
| 1 | 0.0000 | -0.0000 | -0.0000 |
| 100 | 2.3218 | 2.0788 | 2.5667 |
| 200 | 2.4200 | 2.2388 | 2.5752 |
| 300 | 2.4807 | 2.3040 | 2.6477 |
| 400 | 2.5164 | 2.3761 | 2.6700 |
