# Supplementary material for: β-sitosterol inhibits trimethylamine production by regulating the gut microbiota and attenuates atherosclerosis in ApoE–/– mice
Source: Front Cardiovasc Med. 2022 Nov 1;9:986905. doi: 10.3389/fcvm.2022.986905 (PMC9663806; doi:10.3389/fcvm.2022.986905)
Supplement: Supplementary file 1 [file Data_Sheet_1.ZIP › Source data/gut microbiota diversity analysis/CON-EXP/web/res/04.Beta/Adonis/bray_curtis/adonis_results.html]

|  | Df | SumsOfSqs | MeanSqs | F.Model | R2 | Pr(>F) |  |
| --- | --- | --- | --- | --- | --- | --- | --- |
| qiime.data$map[[opts$category]] | 1 | 1.03237 | 1.03237 | 13.679 | 0.57769 | 0.002 | \*\* |
| Residuals | 10 | 0.75469 | 0.07547 | 0.42231 |  |  |  |
| Total | 11 | 1.78706 | 1.00000 |  |  |  |  |
| --- |  |  |  |  |  |  |  |
| Signif. codes: 0 ‘\*\*\*’ 0.001 ‘\*\*’ 0.01 ‘\*’ 0.05 ‘.’ 0.1 ‘ ’ 1 |  |  |  |  |  |  |  |
