# Supplementary material for: β-sitosterol inhibits trimethylamine production by regulating the gut microbiota and attenuates atherosclerosis in ApoE–/– mice
Source: Front Cardiovasc Med. 2022 Nov 1;9:986905. doi: 10.3389/fcvm.2022.986905 (PMC9663806; doi:10.3389/fcvm.2022.986905)
Supplement: Supplementary file 1 [file Data_Sheet_1.ZIP › Source data/gut microbiota diversity analysis/CON-EXP/web/res/04.Beta/Anosim/bray_curtis/anosim_results.html]

| method name | ANOSIM |
| --- | --- |
| test statistic name | R |
| sample size | 12 |
| number of groups | 2 |
| test statistic | 0.912962962962963 |
| p-value | 0.002 |
| number of permutations | 999 |
