# Supplementary material for: β-sitosterol inhibits trimethylamine production by regulating the gut microbiota and attenuates atherosclerosis in ApoE–/– mice
Source: Front Cardiovasc Med. 2022 Nov 1;9:986905. doi: 10.3389/fcvm.2022.986905 (PMC9663806; doi:10.3389/fcvm.2022.986905)
Supplement: Supplementary file 1 [file Data_Sheet_1.ZIP › Source data/gut microbiota diversity analysis/CON-EXP/web/res/05.Differential_analysis/Anova/anova.html]

| OTU | Test-Statistic | P | FDR\_P | Bonferroni\_P | EXP\_mean | CON\_mean | taxonomy |
| --- | --- | --- | --- | --- | --- | --- | --- |
| OTU92 | 148.6625514403292 | 2.514537418285556e-07 | 0.0006512651913359591 | 0.0006512651913359591 | 2.0 | 87.0 | d\_\_Bacteria; p\_\_Verrucomicrobiota; c\_\_Verrucomicrobiae; o\_\_Verrucomicrobiales; f\_\_Akkermansiaceae; g\_\_Akkermansia; s\_\_uncultured bacterium |
| OTU1 | 105.56152832684971 | 1.2396928432689691e-06 | 0.0011692346419889255 | 0.00321080446406663 | 328.5 | 15007.333333333334 | d\_\_Bacteria; p\_\_Verrucomicrobiota; c\_\_Verrucomicrobiae; o\_\_Verrucomicrobiales; f\_\_Akkermansiaceae; g\_\_Akkermansia; s\_\_uncultured bacterium |
| OTU113 | 103.5511332827242 | 1.3543258401416125e-06 | 0.0011692346419889255 | 0.0035077039259667764 | 1.5 | 74.33333333333333 | d\_\_Bacteria; p\_\_Verrucomicrobiota; c\_\_Verrucomicrobiae; o\_\_Verrucomicrobiales; f\_\_Akkermansiaceae; g\_\_Akkermansia; s\_\_uncultured bacterium |
| OTU5 | 70.97390623062759 | 7.464800028444962e-06 | 0.004833458018418113 | 0.019333832073672452 | 143.16666666666666 | 9470.166666666666 | d\_\_Bacteria; p\_\_Verrucomicrobiota; c\_\_Verrucomicrobiae; o\_\_Verrucomicrobiales; f\_\_Akkermansiaceae; g\_\_Akkermansia |
| OTU159 | 58.55009334163035 | 1.734973544698093e-05 | 0.008987162961536121 | 0.04493581480768061 | 8.833333333333334 | 41.166666666666664 | d\_\_Bacteria; p\_\_Firmicutes; c\_\_Clostridia; o\_\_Lachnospirales; f\_\_Lachnospiraceae; g\_\_Blautia; s\_\_uncultured bacterium |
