# Supplementary material for: β-sitosterol inhibits trimethylamine production by regulating the gut microbiota and attenuates atherosclerosis in ApoE–/– mice
Source: Front Cardiovasc Med. 2022 Nov 1;9:986905. doi: 10.3389/fcvm.2022.986905 (PMC9663806; doi:10.3389/fcvm.2022.986905)
Supplement: Supplementary file 1 [file Data_Sheet_1.ZIP › Source data/gut microbiota diversity analysis/CON-EXP/web/res/05.Differential_analysis/Wilcoxon/wilcoxon.html]

|  | mean(CON) | sd(CON) | mean(EXP) | sd(EXP) | p-value | q-value |
| --- | --- | --- | --- | --- | --- | --- |
| Firmicutes | 0.363059175035273 | 0.130711054097688 | 0.769413782609898 | 0.122899686836412 | 0.00507486809794025 | 0.0320517985133068 |
| Verrucomicrobiota | 0.513465847788198 | 0.128452238876162 | 0.00993166791158326 | 0.00348212531162805 | 0.00507486809794025 | 0.0320517985133068 |
| Patescibacteria | 3.45810164052342e-05 | 3.86318091232162e-05 | 0.000304312944366061 | 0.000273902607319387 | 0.0189454075746441 | 0.0639175912960342 |
| Proteobacteria | 0.0260118405400172 | 0.00323240878452057 | 0.0126566520043157 | 0.00790632577630703 | 0.0202405705770775 | 0.0639175912960342 |
| Bacteroidota | 0.0617236561817025 | 0.0465632763617542 | 0.122828312169751 | 0.0573165727566514 | 0.0453275620779722 | 0.11451173577593 |
