# Supplementary material for: β-sitosterol inhibits trimethylamine production by regulating the gut microbiota and attenuates atherosclerosis in ApoE–/– mice
Source: Front Cardiovasc Med. 2022 Nov 1;9:986905. doi: 10.3389/fcvm.2022.986905 (PMC9663806; doi:10.3389/fcvm.2022.986905)
Supplement: Supplementary file 1 [file Data_Sheet_1.ZIP › Source data/gut microbiota diversity analysis/CON-EXP/web/res/allCleanData.stat.html]

| #Trimed | Samples | Sequences | Bases(bp) | AverageLength(bp) |
| --- | --- | --- | --- | --- |
| 16S | 18 | 1166492 | 489027376 | 419.23 |
