# Supplementary material for: β-sitosterol inhibits trimethylamine production by regulating the gut microbiota and attenuates atherosclerosis in ApoE–/– mice
Source: Front Cardiovasc Med. 2022 Nov 1;9:986905. doi: 10.3389/fcvm.2022.986905 (PMC9663806; doi:10.3389/fcvm.2022.986905)
Supplement: Supplementary file 1 [file Data_Sheet_1.ZIP › Source data/gut microbiota diversity analysis/CON-EXP/web/res/cover.html]

|  |  |  |  |
| --- | --- | --- | --- |
| 分析项目 | 内容 | 分析要求 | 目录 |
| **OTU聚类与物种注释** | 数据统计 |  | Data/ |
|  | OTU聚类 |  | OTU\_Taxa/ |
|  | 分类学分析 |  | OTU\_Taxa/ |
| **alpha多样性** | 多样性指数 |  | Estimators/ |
|  | 稀释曲线 |  | Rarefaction/ |
|  | shannon曲线 |  | Shannon\_rarefac/ |
|  | rank abundance | 样本≥2 | Rank\_Abundance/ |
|  | specaccum物种累积曲线 | 样本≥5 | Specaccum/ |
| **物种组成分析** | venn图(花瓣图) | 样本≥2 | Venn/ |
| 组分图 | 样本≥2 | Community/ |  |
| heatmap图 | 样本≥2 | Heatmap/ |  |
| core microbiome分析 | 样本≥2 | Core/ |  |
| 聚类树与组分图结合分析 | 样本≥2 | Hcluster\_bar/ |  |
| 优势物种群落结构组分图 | 样本≥2 | Dominant\_species/ |  |
| **比较分析(beta多样性)** | 组间距离分析 | 分组≥2，每组样本≥3 | Distance/ |
| PCA分析 | 样本≥3 | PCA/ |  |
| PCOA分析 | 样本≥3 | PCOA/ |  |
| 基于UniFrac的PCOA分析 | 样本≥3 | PCOA/ |  |
| Nmds分析 | 样本≥3 | Numds/ |  |
| 基于UniFrac的Nmds分析 | 样本≥3 | Numds/ |  |
| 多样本相似度树状图 | 样本≥3 | Hcluster\_tree/ |  |
| 基于Unifrac的聚树类分析 | 样本≥3 | Hcluster\_tree/ |  |
| anosim | 分组≥2，每组样本≥3 | Anosim/ |  |
| adonis | 分组≥2，每组样本≥3 | Adonis/ |  |
| **差异分析** | LEFSE分析 | 8≥分组≥2，每组样本≥3 | Lefse/ |
|  | ANOVA方差分析 | 分组≥2，每组样本≥3 | Anova/ |
|  | Wilcoxon秩和检验分析 | 分组=2，每组样本≥3 | Wilcoxon/ |
