# Supplementary material for: β-sitosterol inhibits trimethylamine production by regulating the gut microbiota and attenuates atherosclerosis in ApoE–/– mice
Source: Front Cardiovasc Med. 2022 Nov 1;9:986905. doi: 10.3389/fcvm.2022.986905 (PMC9663806; doi:10.3389/fcvm.2022.986905)
Supplement: Supplementary file 1 [file Data_Sheet_1.ZIP › Source data/gut microbiota diversity analysis/CON-EXP/web/res/cover2.html]

|  |  |
| --- | --- |
| - data/ - rawData - --allRawData.stat.xls - --eachSample/ | - 原始序列数据 （后续发送） - 未拼接的原始数据 - 原始数据统计 - 原始数据各样本文件 |
| - valid - --cleanData.stat.xls - --valid.BIO\*.fq - --valid.BIO\*.fq.fasta - --eachSample/ | - 优化序列 - 优化序列统计 - 优化序列fq文件 - 优化序列文件 - 优化序列各样本文件 |
| - results/ - 00.Data - --cleanData.stat.xls - --clean.data.len.pdf - --clean.data.len.dist.txt | - 分析结果 - 优化序列数据统计 - 优化序列数据统计表 - 优化序列长度分布图 - 优化序列长度分布 |
| - 01.OTU\_Taxa - --normalize - --original - ----tax\_summary\_a - ----tax\_summary\_r - ----otu\_table.xls - ----otu\_rep.fasta - ----otu\_seqids.txt - ----otu\_table.biom - ----otu\_taxa\_table.xls - ----otu\_taxa\_table.biom | - 各分类学水平统计表 - 抽平的结果 - 原始的非抽平结果 - 各样本OTU中序列数统计表 - OTU代表序列fasta文件 - 每个OTU中包含的序列编号列表 - biom格式otu表 - OTU分类学综合信息表 - 各分类学水平样本序列数统计表 - 各分类学水平样本序列数相对丰度百分比统计表 - biom格式otu物种分类表 |
| - 02.Alpha - --Estimators - ----Estimate.html - ----otu.\*.summary.xls - --Rarefaction - ----otu.\*.rarefaction.xls - ----rarefaction.All.pdf - --Shannon\_rarefac - ----otu.\*r.shannon.xls - ----r\_shannon.All.pdf - --Rank\_Abundance - ----rankabundance.pdf - --Specaccum - ----specaccum.all.pdf | - Alpha多样性分析 - 多样性分析指数 - 每个样本的指数值 - 所有指数分析结果列表 - 取样深度曲线 - 每个样本在不同取样值的OTU数目表 - 多样本稀释曲线图 - Shannon-wiener曲线 - 每个样本在不同取样值的shannon指数表 - shannon曲线图 - Rank Abundance分析 - 多样本曲线图 - Specaccum分析 - 物种累积曲线图 |
| - 03.Community\_composition - --Venn - ----Venn.pdf - --Community - ----\*.xls - ----bar.All.\*.xls.pdf - --Hclust\_bar - ----treebar\_phylum.pdf - --Heatmap - ----heatmap\_\*.pdf - --Core - ----core\_table\*.xls - ----core\_table\*.biom - ----core\_otu\_size.pdf - --Dominant\_species - ----\*.taxonomy.top\*.pdf - ----\*.taxonomy.top\*.xls | - 物种组成分析 - Venn图 - 文氏图 - 群落结构组分图 - 用于作图的物种丰度表 - 多样本柱状图 - 样本聚类树与柱状图组合分析 - 样本聚类树与柱状图组合分析图 - 群落Heatmap图 - Heatmap图 - Core microbiome分析 - table格式的core microbiome丰度表 - biom格式的core microbiome丰度表 - core microbiome曲线 - 优势物种群落结构组分图 - 多样本优势物种柱状图 - 多样本优势物种列表 |
| - 04.Beta - --Distance - ----\*\_otu\_table.txt.all.pdf - ----Group\_Distances.pdf - --PCA - ----pc.sites.xls - ----pc.importance.xls - ----pca\_rotation.xls - ----pca\_pc\*.pdf - ----3d-pca\*.pdf - --PCOA - ----pcoa\_\*\_otu\_table.txt\_sites.xls - ----pcoa\_\*\_otu\_table.txt\_importance.xls - ----pcoa\_\*\_otu\_table.txt\_rotation.xls - ----pcoa\_\*\_otu\_table.txt\_pc\*.pdf - ----3d-pcoa\*.pdf - --NMDS - ----nmds\_\*\_otu\_table.txt\_sites.xls - ----nmds\_\*\_otu\_table.txt.pdf - --Hcluster\_tree - ----hcluster\_tree\_\*\_curtis.tre - ----hcluster\_tree\_\*\_curtis.pdf - --Anosim - ----anosim\_results.txt - --Adonis - ----anosim\_results.txt - --phylogeny\_tree - ----phylogeny.tre - ----otu\_rep\_aligned.fasta | - Beta多样性比较分析 - 组件距离分析 - 样本间距离热图 - 组间组内距离箱式图 - PCA分析 - 样本在各个维度上的位置，其中PC1为x轴，PC2为y轴，依此类推 - 各维度解释结果的百分比 - 记录了每个OTU对各主成分的贡献度 - PCA图 - 三维PCA图 - PCOA分析 - 样本在各个维度上的位置，其中PC1为x轴，PC2为y轴，依此类推 - 各维度解释结果的百分比 - 记录了每个OTU对各主成分的贡献度 - PCOA图 - 三维PCOA图 - NMDS分析 - 记录了样本在坐标空间中各个维度上的位置 - NMDS分析图 - 多样本相似度树状图 - newick-formatted树文件 - 多样本相似度树状图 - ANOSIM相似性分析 - 组间相似性分析结果 - Adonis分析结果 - 多因素方差分析结果 - 系统发生进化树 - newick-formatted树文件 - 构建系统进化树的fasta文件 |
| - 05.Differential\_analysis - --Lefse - ----lefse\_LDA.xls - ----lefse\_LDA.pdf - ----lefse\_LDA.cladogram.pdf - --Anova - ----anova.txt - --Wilcoxon - ----wilcox.txt | - 差异分析 - Lefse分析结果 - LDA判别分析结果 - LDA分析柱图 - 进化分枝图 - Anova方差分析 - anova方差分析结果列表 - Wilcoxon秩和检验 - wilcox结果列表 |

（注： 参考文献由于文件压缩包较大，邮件不方便发送，有需要可以来信索取。）
