# Supplementary material for: β-sitosterol inhibits trimethylamine production by regulating the gut microbiota and attenuates atherosclerosis in ApoE–/– mice
Source: Front Cardiovasc Med. 2022 Nov 1;9:986905. doi: 10.3389/fcvm.2022.986905 (PMC9663806; doi:10.3389/fcvm.2022.986905)
Supplement: Supplementary file 1 [file Data_Sheet_1.ZIP › Source data/gut microbiota diversity analysis/NON-CON/02.Alpha/01.Estimators/Shannon.group.boxplot.pdf]

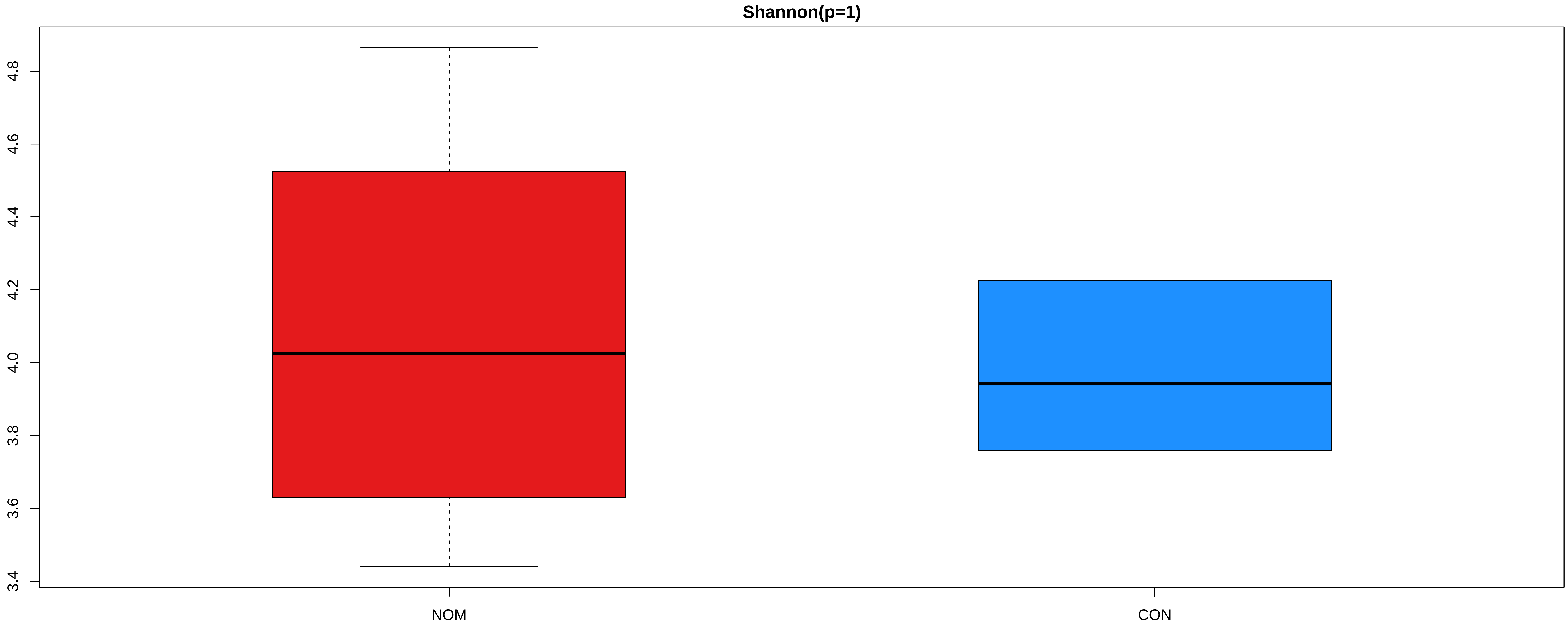

Shannon(p=1)
4.8
4.6
4.4
4.2
4.0
3.8
3.6
3.4
NOM
CON
