# Supplementary material for: β-sitosterol inhibits trimethylamine production by regulating the gut microbiota and attenuates atherosclerosis in ApoE–/– mice
Source: Front Cardiovasc Med. 2022 Nov 1;9:986905. doi: 10.3389/fcvm.2022.986905 (PMC9663806; doi:10.3389/fcvm.2022.986905)
Supplement: Supplementary file 1 [file Data_Sheet_1.ZIP › Source data/gut microbiota diversity analysis/NON-CON/02.Alpha/01.Estimators/coverage.group.boxplot.pdf]

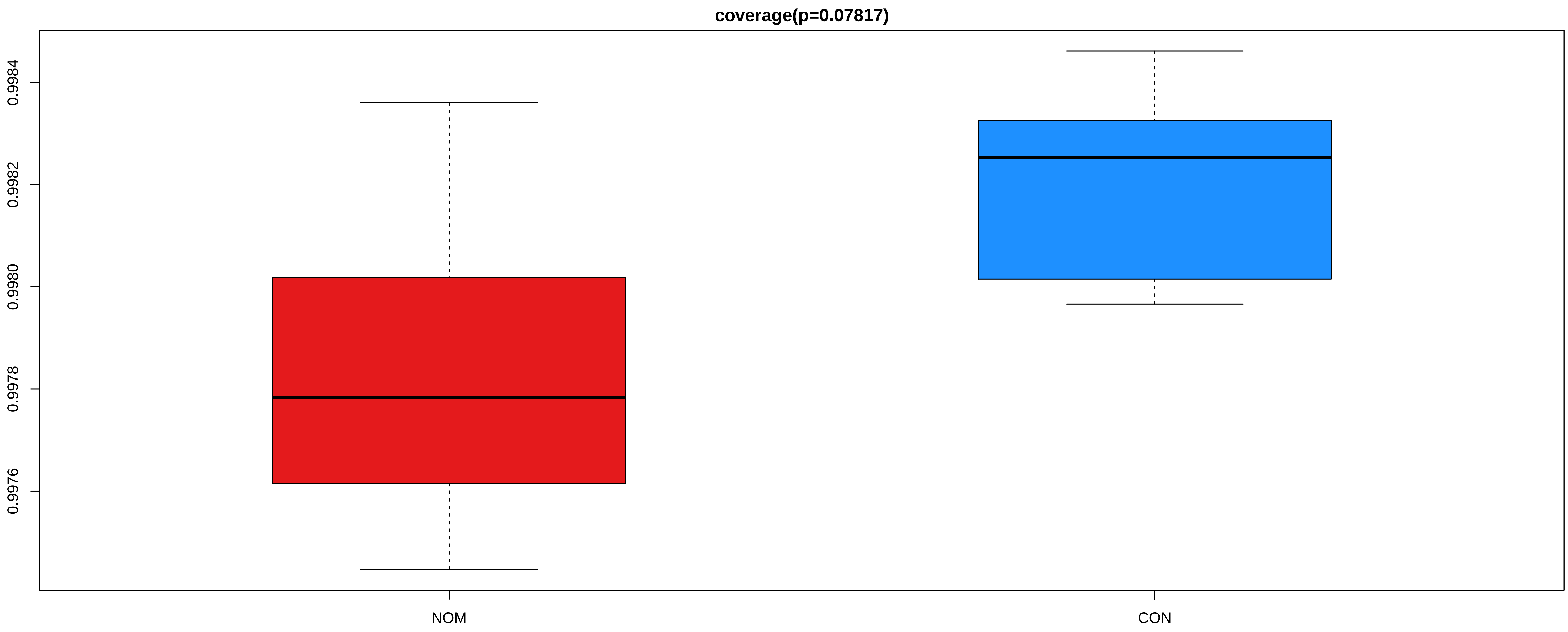

coverage(p=0.07817)
0.9984
0.9982
0.9980
0.9978
0.9976
NOM
CON
