# Supplementary material for: β-sitosterol inhibits trimethylamine production by regulating the gut microbiota and attenuates atherosclerosis in ApoE–/– mice
Source: Front Cardiovasc Med. 2022 Nov 1;9:986905. doi: 10.3389/fcvm.2022.986905 (PMC9663806; doi:10.3389/fcvm.2022.986905)
Supplement: Supplementary file 1 [file Data_Sheet_1.ZIP › Source data/gut microbiota diversity analysis/NON-CON/02.Alpha/02.Rarefaction/rarefaction.All.pdf]

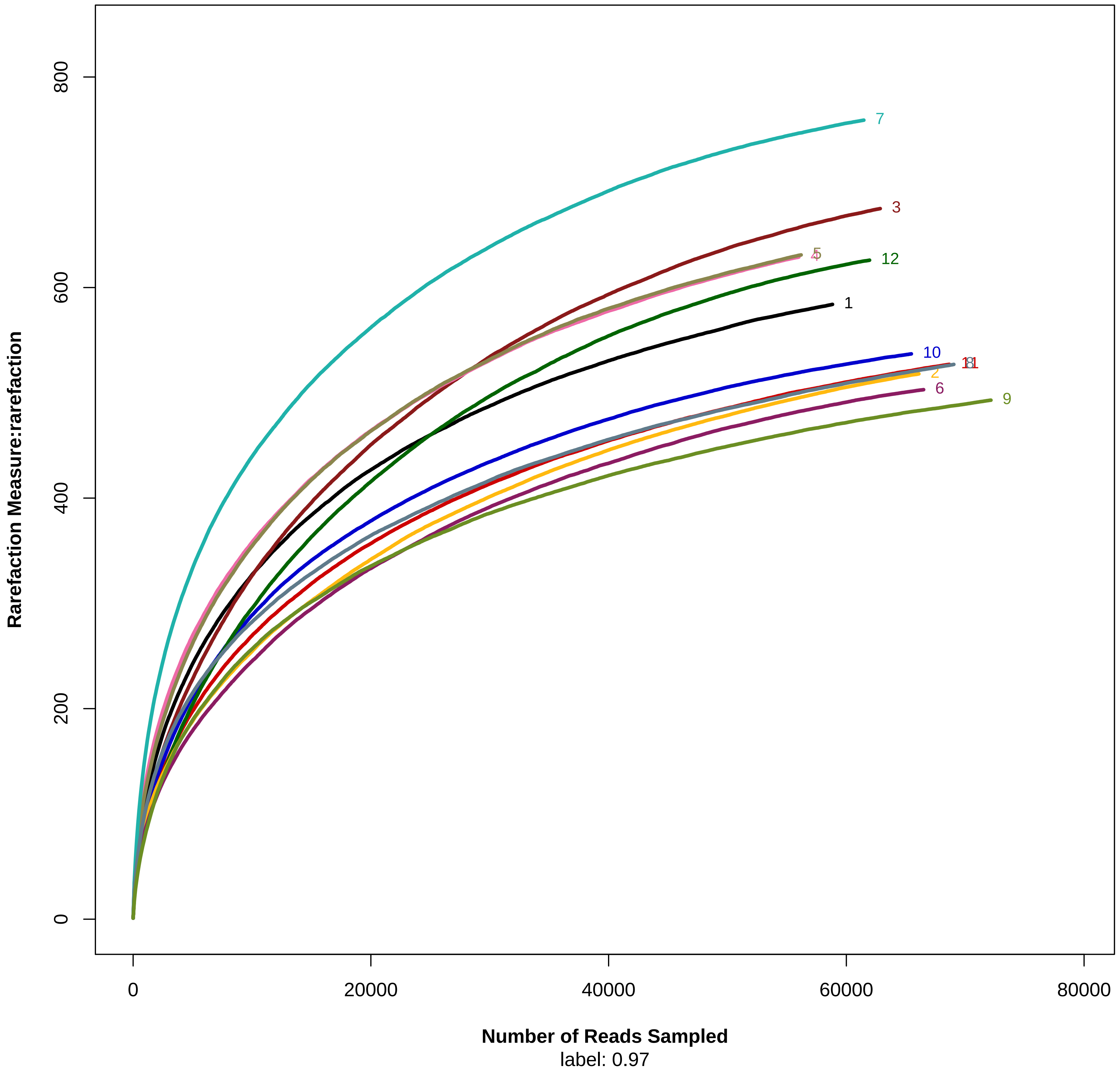

Rarefaction Measure:rarefaction
Number of Reads Sampled
label: 0.97
0
200
400
600
800
0
20000
40000
60000
80000
7
3
5
4
12
1
10
11
8
2
6
9
