# Supplementary material for: β-sitosterol inhibits trimethylamine production by regulating the gut microbiota and attenuates atherosclerosis in ApoE–/– mice
Source: Front Cardiovasc Med. 2022 Nov 1;9:986905. doi: 10.3389/fcvm.2022.986905 (PMC9663806; doi:10.3389/fcvm.2022.986905)
Supplement: Supplementary file 1 [file Data_Sheet_1.ZIP › Source data/gut microbiota diversity analysis/NON-CON/02.Alpha/03.Shannon_rarefac/r_shannon.All.pdf]

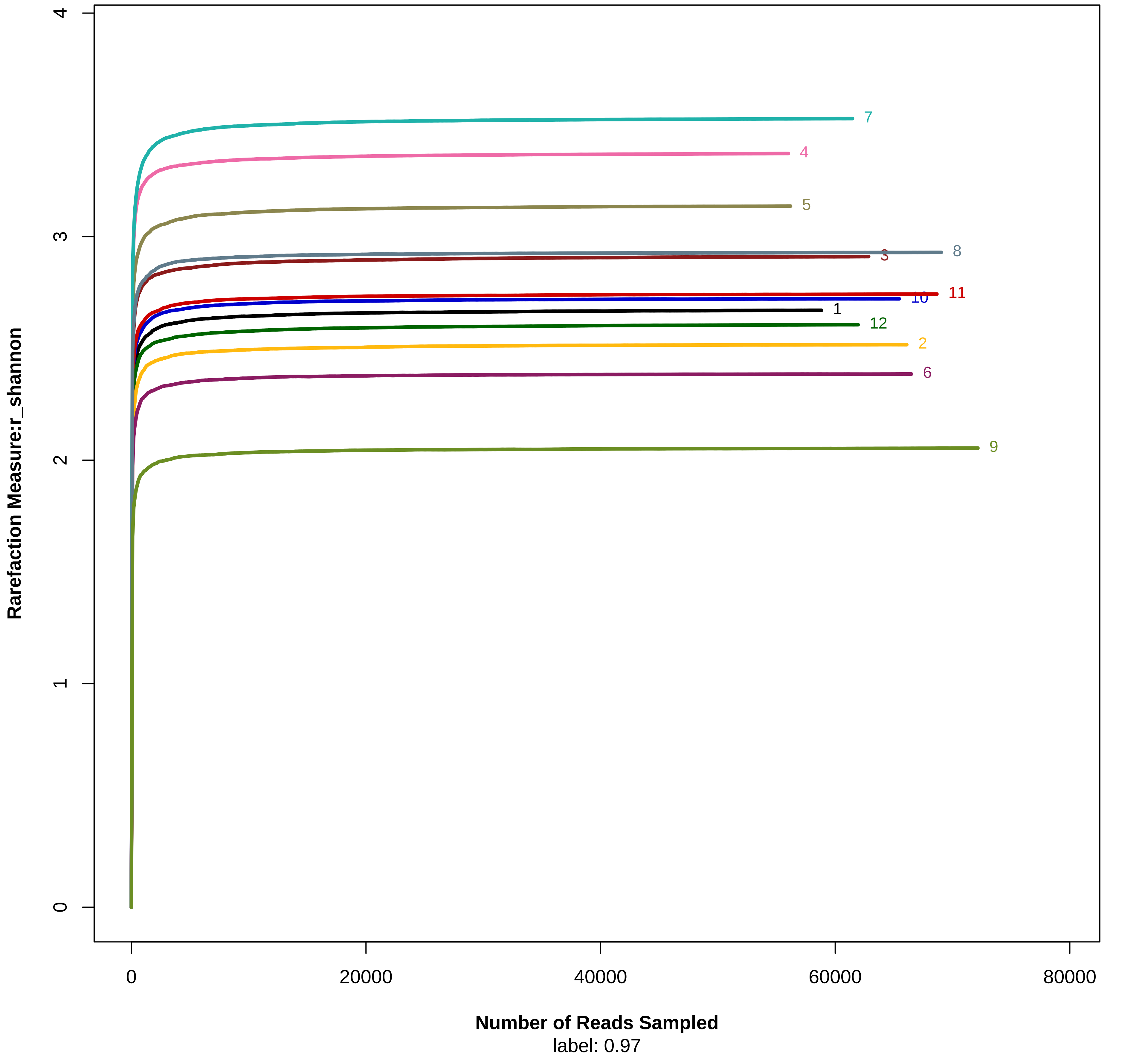
Rarefaction Measure:r_shannon
0
1
2
3
4
7
4
5
8
3
11
10
1
12
2
6
9
0
20000
40000
60000
80000
Number of Reads Sampled
label: 0.97
