# Supplementary material for: β-sitosterol inhibits trimethylamine production by regulating the gut microbiota and attenuates atherosclerosis in ApoE–/– mice
Source: Front Cardiovasc Med. 2022 Nov 1;9:986905. doi: 10.3389/fcvm.2022.986905 (PMC9663806; doi:10.3389/fcvm.2022.986905)
Supplement: Supplementary file 1 [file Data_Sheet_1.ZIP › Source data/gut microbiota diversity analysis/NON-CON/02.Alpha/04.Rank_abundance/rankabundance.pdf]

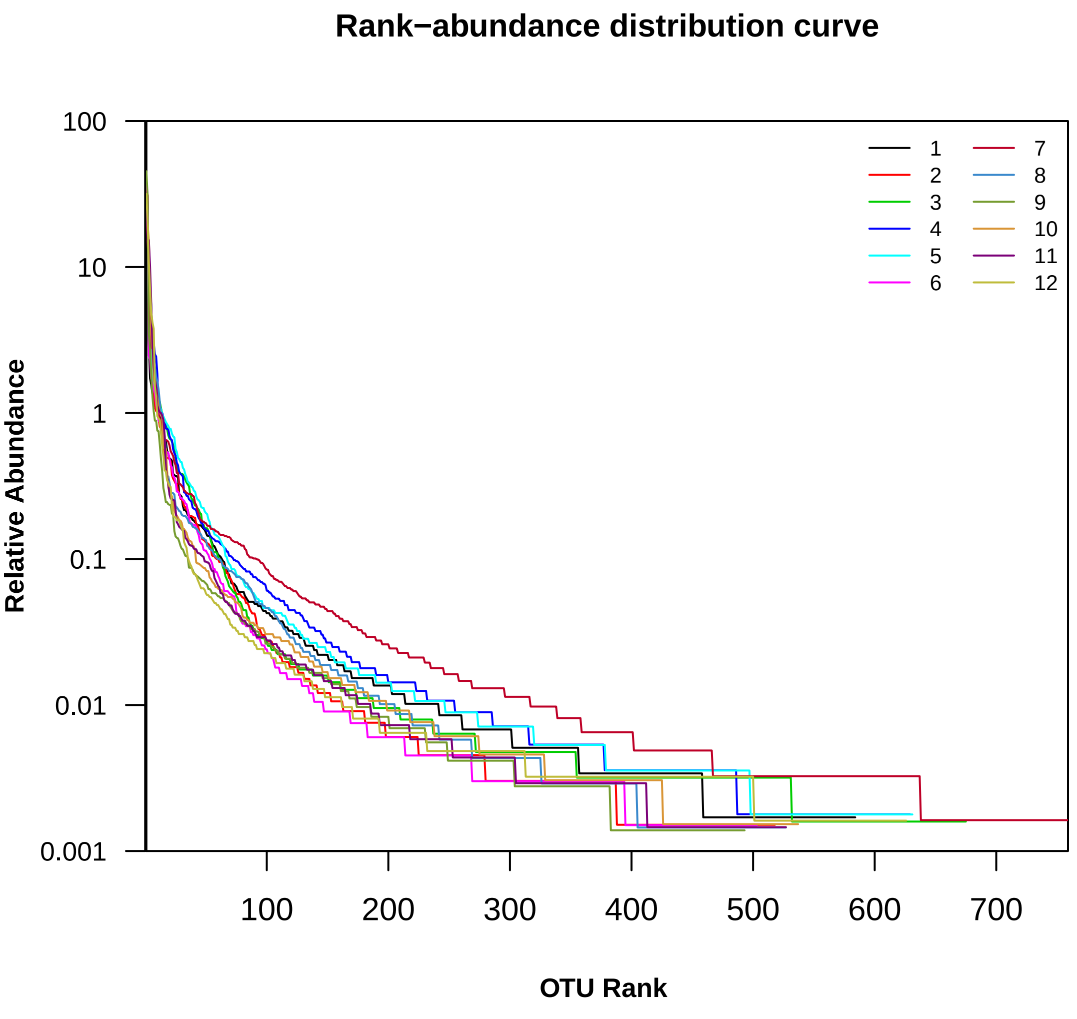
Rank−abundance distribution curve
Relative Abundance
100
10
1
0.1
0.01
0.001
OTU Rank
100
200
300
400
500
600
700
1
2
3
4
5
6
7
8
9
10
11
12
